# Achieving target plasma concentrations of beta-lactam antibiotics in critically ill patients: a retrospective study of full-dose administration in the first 24 hours

**DOI:** 10.1007/s43440-026-00836-8

**Published:** 2026-02-25

**Authors:** Milada Halačová, Marie Mieresová, Jan Kubele, Eva Klapková, Dalibor Černý, Petr Waldauf, František Duška

**Affiliations:** 1 Department of Clinical Pharmacy, Motol and Homolka University Hospital, Prague, Czech Republic; 2 Department of Anaesthesia and Intensive Care Medicine, Third Faculty of Medicine, Charles University, FNKV University Hospital, Prague, Czech Republic; 3 Department of Pharmacology, 2nd Faculty of Medicine, Charles University and Motol and Homolka University Hospital, Prague, Czech Republic; 4 Department of Clinical Microbiology, Motol and Homolka University Hospital, Prague, Czech Republic; 5 Department of Medical Chemistry and Clinical Biochemistry, 2nd Faculty of Medicine, Charles University and Motol and Homolka University Hospital, Prague, Czech Republic; 6 Institute of Pharmacology, 1st Faculty of Medicine, Charles University, Prague, Czech Republic

**Keywords:** Beta-lactams, Critical care, Pharmacokinetics, Pharmacodynamics

## Abstract

**Background:**

Beta-lactams are the cornerstone of therapy for the treatment of the most serious infections in the intensive care unit (ICU) setting. However, individualization of dosing remains challenging because pharmacokinetics are highly variable and difficult to predict in critically ill patients. The objective of this study was to evaluate whether administration of full-dose beta-lactam antibiotics to critically ill subjects during the first 24 h of treatment, irrespective of baseline renal function, achieves predefined target concentration ranges (Cmin [free minimum concentration] 1–10 × MIC [minimum inhibitory concentration] and 4–10 × MIC).

**Methods:**

This trial was a retrospective, observational cohort study. A total of 377 critically ill patients over 18 years admitted to the ICU who received full doses of meropenem, piperacillin–tazobactam, or cefepime were analyzed. Blood sampling for determination of antibiotic trough concentration was performed 24 h after initiation of full-dose therapy.

**Results:**

When targeting a Cmin of 4–10 × MIC, a high proportion of patients remained underdosed (42.7%, *N* = 161). In contrast, when targeting a Cmin of 1–10 × MIC, only 13.3% (*N* = 50) of patients failed to reach the target range. Seventy-four patients (19.6%) were found to be above the defined target range (Cmin > 10 × MIC). Reduced glomerular filtration rate (GFR) was a consistent independent predictor of achieved plasma level across the overall population, as well as within individual subsets. No adverse effects (neurotoxicity, hematotoxicity, hepatotoxicity) were observed.

**Conclusions:**

With full-dose beta-lactam administration, targeting a Cmin of 4–10 × MIC results in a high proportion of patients being underdosed, whereas a target of 1–10 × MIC is achieved in the majority of patients. Our results highlight the importance of therapeutic drug monitoring (TDM) of beta-lactam antibiotics in critically ill patients. Further research in this field is warranted.

**Clinical trial number:**

Not applicable.

**Supplementary Information:**

The online version contains supplementary material available at 10.1007/s43440-026-00836-8.

## Introduction

Beta-lactams are the antibiotics of choice for the treatment of the most serious infections in the intensive care unit (ICU) setting due to their bactericidal activity and the wide range of molecules with the desired spectrum. Despite years of efforts to optimize antibiotic dosing in the ICU setting, there are no clear evidence-based guidelines on how to approach the initial phase of treatment. This is crucial in the treatment of sepsis and severe infections because pathophysiological changes in these conditions can substantially alter drug pharmacokinetics, making standard dosing regimens unreliable [1, 2]. Several factors influence antibiotic exposure during the initial phase of therapy. The volume of distribution fluctuates because of fluid resuscitation and changes in endothelial permeability. Plasma volume is often initially reduced but then increases rapidly after fluid resuscitation. Additionally, plasma protein concentrations decrease, thereby altering the protein dissociation constant (pKa) and binding capacity. Adequate antibiotic concentrations must furthermore be achieved both in the bloodstream and within tissues that are the primary source of infection. Finally, most beta-lactams are eliminated renally in an unchanged form (70–90%), and glomerular filtration rate (GFR) may vary widely in critically ill patients, ranging from severely reduced or absent renal function to augmented renal clearance.

MICs (minimum inhibitory concentration) clinical breakpoints define the antibiotic concentration used to indicate whether an infection caused by a specific bacterial isolate is likely to be treatable in a patient [3]. According to EUCAST, these breakpoints can differ substantially between pathogens. For example, cefepime MIC breakpoints are 0.2 mg/L for *Viridans* group streptococci, 0.25 mg/L for *Haemophilus influenzae*, 2 mg/L for *Streptococcus pneumoniae*, 4 mg/L for Enterobacterales, and 8 mg/L for Pseudomonas species, etc. Consequently, initial empirical therapy should aim to cover the highest expected MIC among likely pathogens. Based on surveillance data, MIC breakpoints for Pseudomonas species are therefore most commonly selected as therapeutic targets in critically ill patients [4, 5].

Selecting the correct dose and dosing intervals can be challenging to individualize, as many of the relevant pharmacokinetic determinants are unknown at treatment initiation. Several studies have concluded that the optimal pharmacokinetic/pharmacodynamic (PK/PD) target for beta-lactams is defined as 100% fT > MIC, i.e., the free (unbound) antibiotic concentration remaining above MIC throughout the entire dosing interval, and even higher targets such as 100% fT > 4 × MIC have been proposed [7, 8]. Concentrations not exceeding 10 times the MIC over the entire dosing interval are generally considered safe [9]. Although therapeutic drug monitoring is useful, it often does not provide results early enough to guide the initial treatment phase. Some authors recommend beta-lactam dose reduction after an initial loading dose in patients with reduced GFR; however, due to the wide therapeutic range of beta-lactams, the risk of underdosing generally outweighs the risk of overdosing [2, 6]. Underdosing can lead to treatment failure, death, and an increased risk of emergent resistant strains.

In light of the above, our antibiotic advisory center has adopted a pragmatic policy of administering full-dose beta-lactam therapy for the first 24 h, in accordance with the summary of product characteristics, to ICU patients with severe infections, regardless of their renal function at presentation. This retrospective observational study investigated the impact of this dosing regimen on achieving predefined PK/PD targets and examined factors contributing to antibiotic underdosing and overdosing. We hypothesized that full-dose beta-lactam administration during the initial 24 h of therapy in critically ill patients, irrespective of renal function status, would result in trough concentrations (Cmin) within the defined target range and a minimal risk of toxicity.

## Materials and methods

### Study design and settings

This retrospective, observational, cohort study was conducted at Na Homolce Hospital in Prague, Czech Republic. Procedures followed in this study adhered to the ethical standards of Na Homolce Hospital and the Declaration of Helsinki (1975), as most recently amended.

Na Homolce Hospital is a tertiary care center with 380 beds, including 75 ICU beds with an established antimicrobial stewardship program, in which each indication for parenteral antibiotic therapy is reviewed and authorized online by a dedicated antibiotic team comprising a clinical microbiologist and a clinical pharmacist. The consultation service is available 24/7, and all antibiotics included in this study are on the hospital’s list of essential medicines. All ICU beds at Na Homolce Hospital provide acute care (39 surgical, 28 medical, and 8 mixed beds). Administration of a full initial beta-lactam dose during the first 24 h of therapy, followed by subsequent plasma sampling, is standard institutional practice.

### Participants

All consecutive critically ill patients admitted to the ICU between January 2019 and December 2022 were eligible for inclusion if they received one of the investigational antibiotics for the treatment of an acute infection, including sepsis, pneumonia, abdominal infection, soft tissue infection, cardiovascular infection with hematological dissemination, central nervous system infection, sinusitis, urological infection, or other infections. Inclusion criteria were age over 18 years and the presence of an acute infection requiring treatment with meropenem, cefepime, or piperacillin–tazobactam (piperacillin component) administered at doses considered to be full for the given indication. Dose adequacy was individually assessed by the treating team in accordance with available clinical evidence and the approved Summary of Product Characteristics (SmPC) [10–12]. All antibiotics were administered as intermittent infusions over 30 min. Exclusion criteria comprised prophylactic antibiotic use, dose reduction within the first 24 h after therapy initiation, pregnancy, and diagnoses of burns or cystic fibrosis. Informed consent for publication of anonymized and aggregated data was obtained from patients or, when applicable, from their family members in cases of patient death. The study protocol was approved by the Na Homolce Hospital Research Ethics Board (approval number: 09_F_NNH_027; registration number: 11).

Patients were analyzed as a single cohort and subsequently in a predefined subgroup analysis, including patients treated with doses generally considered to be the maximum according to the SmPC (“conventional” maximum doses: piperacillin–tazobactam 4.5 g every 6 h, meropenem 2 g every 8 h, and cefepime 2 g every 8 h). Throughout this manuscript, this subgroup is referred to as the “CD patients”, defined as those receiving conventional maximum doses.

### Data processing

We collected data on patient demographics, indications for antibiotic therapy, initial antibiotic dose and dosing frequency, Cmin measured after the first 24 h of treatment, glomerular filtration rate (GFR) calculated using the Jelliffe and Cockcroft-Gault (C-G) equations, the use of continuous renal replacement therapy (CRRT; blood flow 150 mL/min, dialysate flow 2000 mL/h), vasopressor requirements, occurrence of acute kidney injury (AKI), 30-day mortality, and a priori specified adverse events related to antibiotic administration. Plasma beta-lactam concentrations were interpreted in relation to the susceptibility of known or suspected pathogens. Because blood sampling was performed within 24 h of treatment initiation, MIC values for cultured pathogens were not yet available. Therefore, antibiotic concentrations were evaluated using clinical breakpoints defined by the European Committee on Antimicrobial Susceptibility Testing (EUCAST) [13]. The MIC targets applied for analysis were 8 mg/L for meropenem, 8 mg/L for cefepime, and 16 mg/L for piperacillin.

### Data sources/measurement

Blood sampling for the determination of antibiotic trough concentrations was performed after 24 h of full dosing, and immediately before the 4th, 5th, or 7th dose, depending on the antibiotic dosing schedule (every 8, 6, or 4 h). Blood samples were obtained from a peripheral vein into heparin-coated tubes (Sarsted, Nümbrecht, Germany), immediately centrifuged, and the supernatant was separated in a cooled centrifuge and stored at -80 °C. Plasma concentrations of all antibiotics were measured using liquid chromatography coupled with tandem mass spectrometry (LC-MS/MS) on an Agilent Technologies 1290 Infinity II LC system with 6470 Triple Quad (Agilent Technologies, Santa Clara, USA). Mass spectrometric detection was performed on a triple-quadrupole instrument operated in positive electrospray ionization mode using dynamic multiple reaction monitoring (MRM). Chromatographic separation was achieved on an Agilent Eclipse Plus C18 column (1.8 μm, 3.0 × 50 mm) with gradient elution using mobile phases consisting of 0.1% formic acid in water (A) and 0.1% formic acid in 90% acetonitrile (B). For sample preparation, plasma aliquots (50 µL) were precipitated with acetonitrile containing isotopically labelled internal standards, mixed, and then centrifuged.

### Outcomes

To reflect evolving practices in β-lactam therapeutic drug monitoring (TDM), we deliberately present both analytical approaches. The 1–10 × MIC range was used as the primary outcome (i.e., meropenem 8–80 mg/L, cefepime 8–80 mg/L, and piperacillin 16–160 mg/L), and the 4–10 × MIC range (i.e., meropenem 32–80 mg/L, cefepime 32–80 mg/L, and piperacillin 64–160 mg/L), recommended by earlier consensus guidelines, was considered the secondary outcome [7, 8]. Further secondary outcomes were defined as: (i) the proportion of patients with concentrations below the target range and factors associated with underexposure; (ii) the proportion of patients with concentrations above the target range and factors associated with overexposure; and (iii) the proportion of patients experiencing adverse effects. Adverse effects were defined as clinical or laboratory manifestations of organ toxicity, including neurotoxicity (myoclonus and/or seizures, encephalopathy, altered mental status, peripheral neuropathy), hematotoxicity (clinically or laboratory-significant abnormalities in the blood count assessed by the treating physician as potentially causally related to antibiotic treatment), hepatotoxicity (elevation of liver enzyme levels to ≥ 3 times the upper limit of normal [ULN], assessed by the treating physician as potentially causally related to antibiotic treatment), and kidney adverse outcomes. Kidney adverse outcomes were defined as progression to acute kidney injury according to Kidney Disease: Improving Global Outcomes (KDIGO) criteria, including an increase in serum creatinine (SCr) of ≥ 0.3 mg/dL [≥ 26.5 µmol/L] within 48 h, and/or an increase in SCr to ≥ 1.5 times baseline known or presumed to have occurred within the preceding 7 days, and/or urine output < 0.5 mL/kg/h for 6 h. The occurrence of adverse events was assessed throughout the entire course of antibiotic treatment [14].

### Statistical methods

We used standard descriptive statistics, expressing data as mean (standard deviation) or median (interquartile range), depending on the normality of the distribution as determined by the Shapiro-Wilk test. Univariate and multivariate regression analyses, as well as random forest analyses, were performed using R software version 4.2.2 (2022-10-31 ucrt) with R Markdown. Results with *p* < 0.05 were considered statistically significant.

## Results

**Fig. 1:**
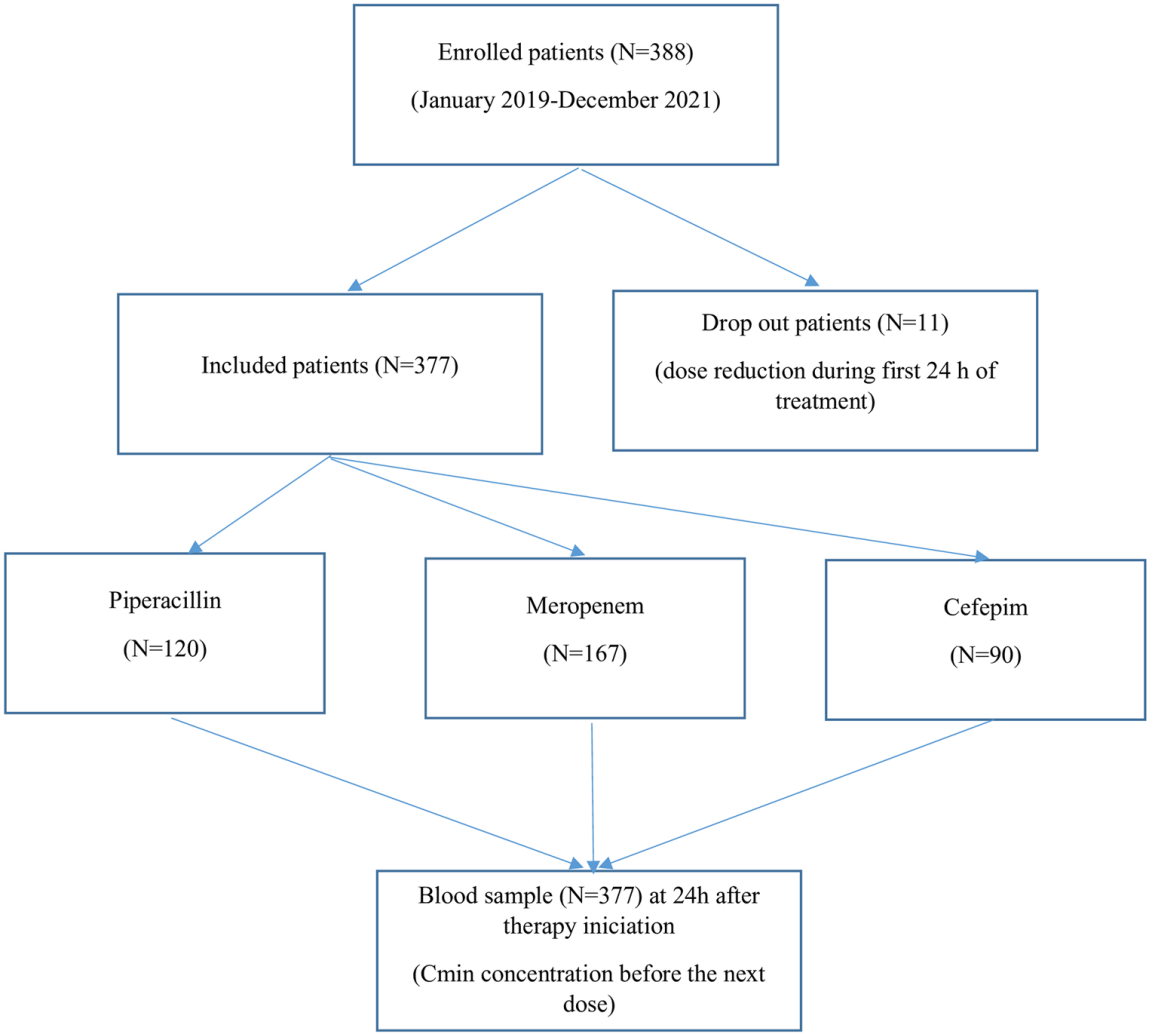
The study flowchart. Cmin – trough concentration, ICU – intensive care unit, MIC – minimum inhibitory concentration, LC–MS/MS – liquid chromatography tandem mass spectrometry

Out of 388 included subjects, we analyzed complete datasets from 377 critically ill adults (Fig. 1). Eleven patients were excluded due to dose reduction within the first 24 h of treatment. Baseline characteristics are summarized in Table S1. The majority of patients were male (71.6%, *N* = 270), with a mean age of 67.1 ± 12.6 years, and a body mass index (BMI) of 29.1 ± 6.0 kg/m^2^. The mean GFR was 43 ± 34.7 mL/min (Jelliffe) and 57.6 ± 43.8 mL/min (C-G). AKI was observed in 60.2% (*N* = 227), and 19.1% (*N* = 72) were receiving CRRT.

The CD subpopulation was predominantly male as well (76.9%, *N* = 176), with a mean age of 66.0 ± 12.1 years and a mean BMI of 29.6 ± 5.4 kg/m^2^. The GFR of the CD subpopulation was 41.3 ± 43.7 mL/min (Jelliffe) and 55.7 ± 48.5 mL/min (C-G), and AKI was present in 57.6% of the subjects (*N* = 132). More detailed characteristics of the CD subpopulation are shown in Table S5 (a, b).

**Fig. 2:**
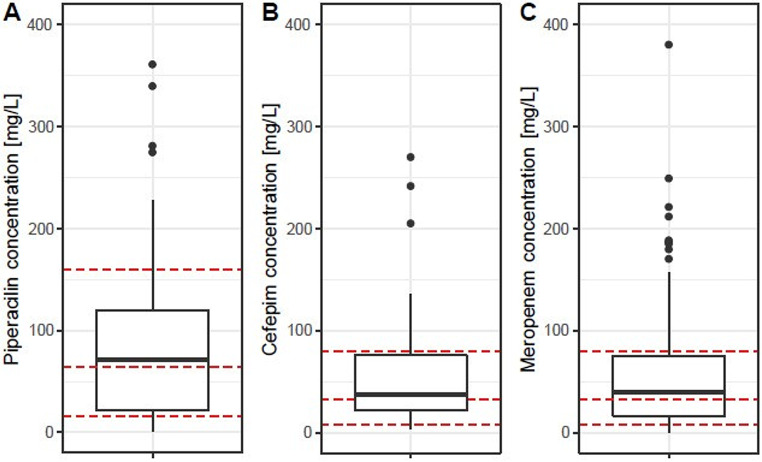
Distribution of beta-lactam trough plasma concentrations (Cmin) in the entire study population of critically ill ICU patients (*N* = 377) treated with beta-lactam doses considered to be full for the present indication: **A** – piperacillin–tazobactam, **B** – cefepime, **C** – meropenem. Red dashed lines correspond to MIC 1, 4, and 10 for individual antibiotics. Retrospective observational cohort study conducted at Na Homolce Hospital, Prague, Czech Republic (January 2019–December 2022) involving critically ill ICU patients (N = 377) treated with piperacillin–tazobactam, cefepime, or meropenem for acute infections. Antibiotic plasma trough concentrations (Cmin) were measured after 24 h of full-dose therapy using LC–MS/MS and compared with EUCAST breakpoints. The MIC values for piperacillin, cefepime, and meropenem were set at 16 mg/L, 8 mg/L, and 8 mg/L, respectively. Cmin – trough concentration, EUCAST – European Committee on Antimicrobial Susceptibility Testing, ICU – intensive care unit, MIC – minimum inhibitory concentration, LC–MS/MS – liquid chromatography tandem mass spectrometry

**Fig. 3:**
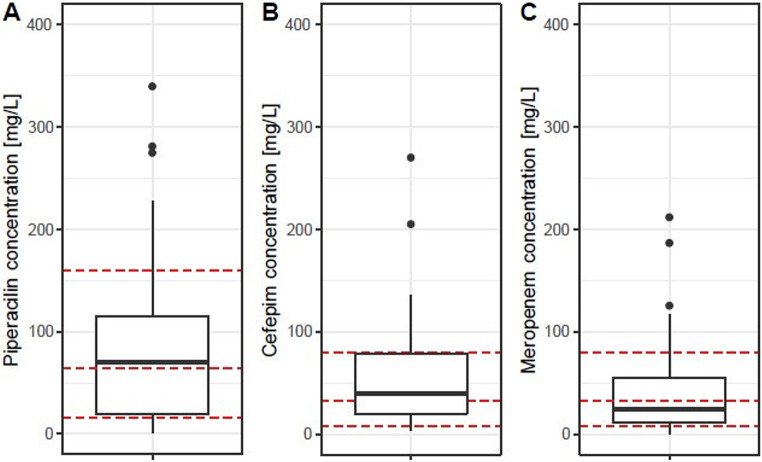
Distribution of beta-lactam trough plasma concentrations (Cmin) measured in the CD subpopulation (*N* = 229) treated with conventional maximum doses of beta-lactams: **A** – piperacillin–tazobactam 4.5 g every 6 h, **B** – cefepime 2 g every 8 h, or **C** – meropenem 2 g every 8 h. The results are reported in relation to the target Cmin range of 1–10× MIC. Red dashed lines correspond to MIC 1, 4, and 10 for individual antibiotics. Retrospective observational cohort study conducted at Na Homolce Hospital, Prague, Czech Republic (January 2019–December 2022) involving critically ill ICU patients (N = 377) treated with piperacillin–tazobactam, cefepime, or meropenem for acute infections. Antibiotic plasma trough concentrations (Cmin) were measured after 24-hour full-dose treatment with LC–MS/MS. Antibiotic plasma trough concentrations (Cmin) were measured after 24 h of full-dose therapy using LC–MS/MS and compared with EUCAST breakpoints. The MIC values for piperacillin, cefepime, and meropenem were set at 16 mg/L, 8 mg/L, and 8 mg/L, respectively. Cmin – trough concentration, EUCAST – European Committee on Antimicrobial Susceptibility Testing, ICU – intensive care unit, MIC – minimum inhibitory concentration, LC–MS/MS - liquid chromatography tandem mass spectrometry, CD subpopulation – patients treated with conventional maximum doses of beta-lactam antibiotics

Plasma concentrations across all patients are shown in Fig. 2, while concentrations within the CD subpopulation are depicted in Fig. 3.

For the target 1–10 × MIC, 13.3% (*N* = 50) of all subjects were found to be below the target range, and 19.6% (*N* = 74) were above. Multivariate analysis revealed GFR (both Jelliffe and C-G) and age (C-G) as independent predictors of subtherapeutic plasma levels, and GFR (Jelliffe, C-G), BMI (Jelliffe), male sex (Jelliffe, C-G), and CRRT (C-G) as independent predictors of supratherapeutic plasma levels (Tables S2, S4).

Among CD patients, 68.1% (*N* = 156) achieved the desired target Cmin 1–10 × MIC, while 17.5% (*N* = 40) were below and 14.4% (*N* = 33) were above the target range. In the subpopulation receiving conventional maximum dosing, GFR (Jelliffe, C-G) remained an independent predictor for both underdosing and overdosing (Tables S6, S8).

For the 4–10 × MIC target, only 37.7% (*N* = 142) of all subjects achieved target concentrations, while 42.7% (*N* = 161) were subtherapeutic, and 19.6% (*N* = 74) were supratherapeutic. Multivariate analysis revealed age (C-G) and GFR (Jelliffe, C-G) as independent predictors of subtherapeutic plasma levels (Tables S3, S4).

Among CD patients, 36.7% (*N* = 84) achieved the desired target Cmin 4–10 × MIC, 36.7% (*N* = 84) remained below the target range, and 14.4% (*N* = 33) exceeded the target. In the subpopulation receiving conventional maximum dosing, GFR (Jelliffe, C-G) and vasopressor dose (C-G) remained independent predictors of underdosing (Table S7, S8).

**Fig. 4:**
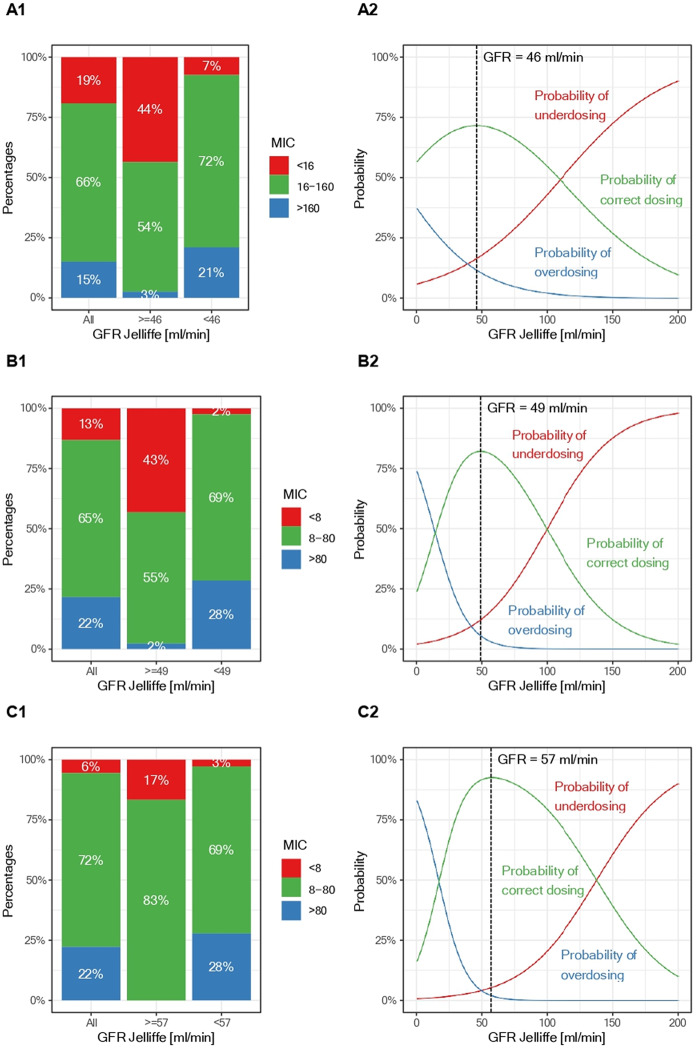
Attainment of therapeutic beta-lactam plasma levels targeting a Cmin of 1–10 × MIC measured in the entire study population of critically ill ICU patients (*N* = 377) treated with piperacillin–tazobactam, meropenem, or cefepime at doses considered to be full for the present indication, using the GFR Jelliffe equation. Exploratory data analysis for: **A1** – piperacillin–tazobactam, **B1** – meropenem, **C1** – cefepime, illustrating the proportion of patients (%) in each MIC-based exposure category (underdosing, target attainment, overdosing) across different categories of the GFR Jelliffe. Univariate logistic regression for: **A2** – piperacillin–tazobactam, **B2** – meropenem, **C2** – cefepime, illustrating the predicted probability of underdosing, correct dosing, and overdosing as a function of the GFR Jelliffe. Dashed vertical lines indicate GFR thresholds associated with optimal target attainment. Retrospective observational cohort study conducted at Na Homolce Hospital, Prague, Czech Republic (January 2019–December 2022) involving critically ill ICU patients (N = 377) treated with piperacillin–tazobactam, meropenem, or cefepime for acute infections. Antibiotic plasma trough concentrations were measured after 24 h of full-dose therapy using LC–MS/MS and compared with EUCAST breakpoints. The MIC values for piperacillin, meropenem, and cefepime were set at 16 mg/L, 8 mg/L, and 8 mg/L, respectively. Cmin – trough concentration, EUCAST – European Committee on Antimicrobial Susceptibility Testing, ICU – intensive care unit, MIC – minimum inhibitory concentration, LC–MS/MS - liquid chromatography tandem mass spectrometry, GFR Jelliffe – glomerular filtration rate estimated using the Jelliffe equation

**Fig. 5:**
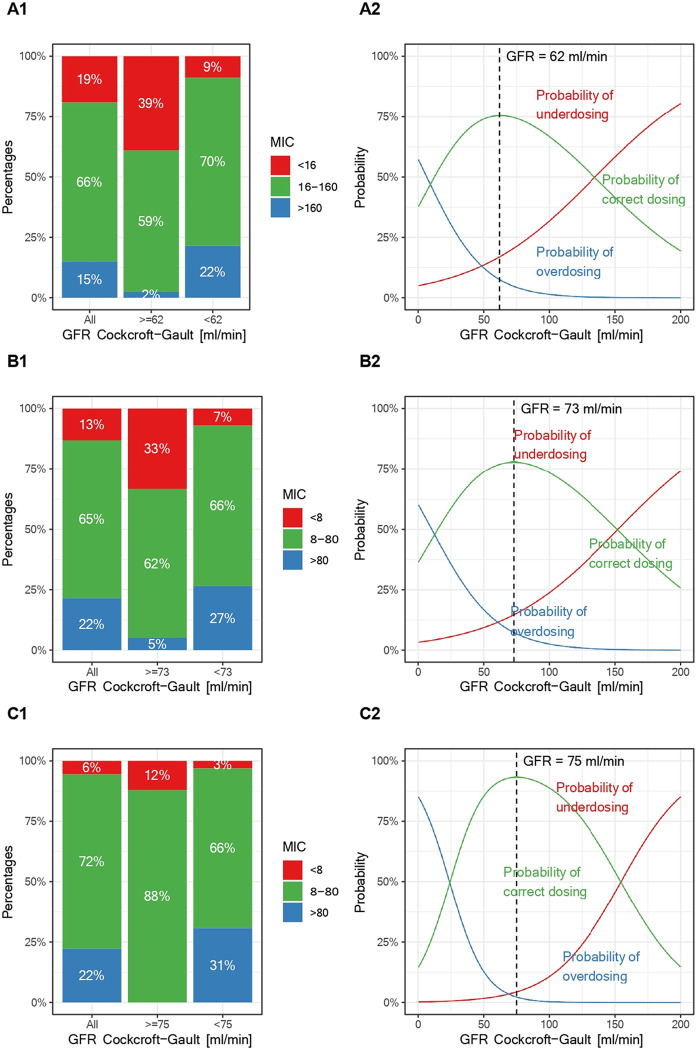
Attainment of therapeutic beta-lactam plasma levels targeting a Cmin of 1–10 × MIC measured in the entire study population of critically ill ICU patients (*N* = 377) treated with piperacillin–tazobactam, meropenem, or cefepime at doses considered to be full for the present indication, using the GFR Cockcroft-Gault equation. Exploratory data analysis for: **A1** – piperacillin–tazobactam, **B1** – meropenem, **C1** – cefepime, illustrating the proportion of patients (%) in each MIC-based exposure category (underdosing, target attainment, overdosing) across different categories of the GFR Cockcroft-Gault. Univariate logistic regression for: **A2** – piperacillin–tazobactam, **B2** – meropenem, **C2** – cefepime, illustrating the predicted probability of underdosing, correct dosing, and overdosing as a function of the GFR Cockcroft-Gault. Dashed vertical lines indicate GFR thresholds associated with optimal target attainment. Retrospective observational cohort study conducted at Na Homolce Hospital, Prague, Czech Republic (January 2019–December 2022) involving critically ill ICU patients (N = 377) treated with piperacillin–tazobactam, meropenem, or cefepime for acute infections. Antibiotic plasma trough concentrations were measured after 24 h of full-dose therapy using LC–MS/MS and compared with EUCAST breakpoints. The MIC values for piperacillin, meropenem, and cefepime were set at 16 mg/L, 8 mg/L, and 8 mg/L, respectively. Cmin – trough concentration, EUCAST – European Committee on Antimicrobial Susceptibility Testing, ICU – intensive care unit, MIC – minimum inhibitory concentration, LC–MS/MS - liquid chromatography tandem mass spectrometry, GFR Cockcroft-Gault – glomerular filtration rate estimated using the Cockcroft-Gault equation

**Fig. 6:**
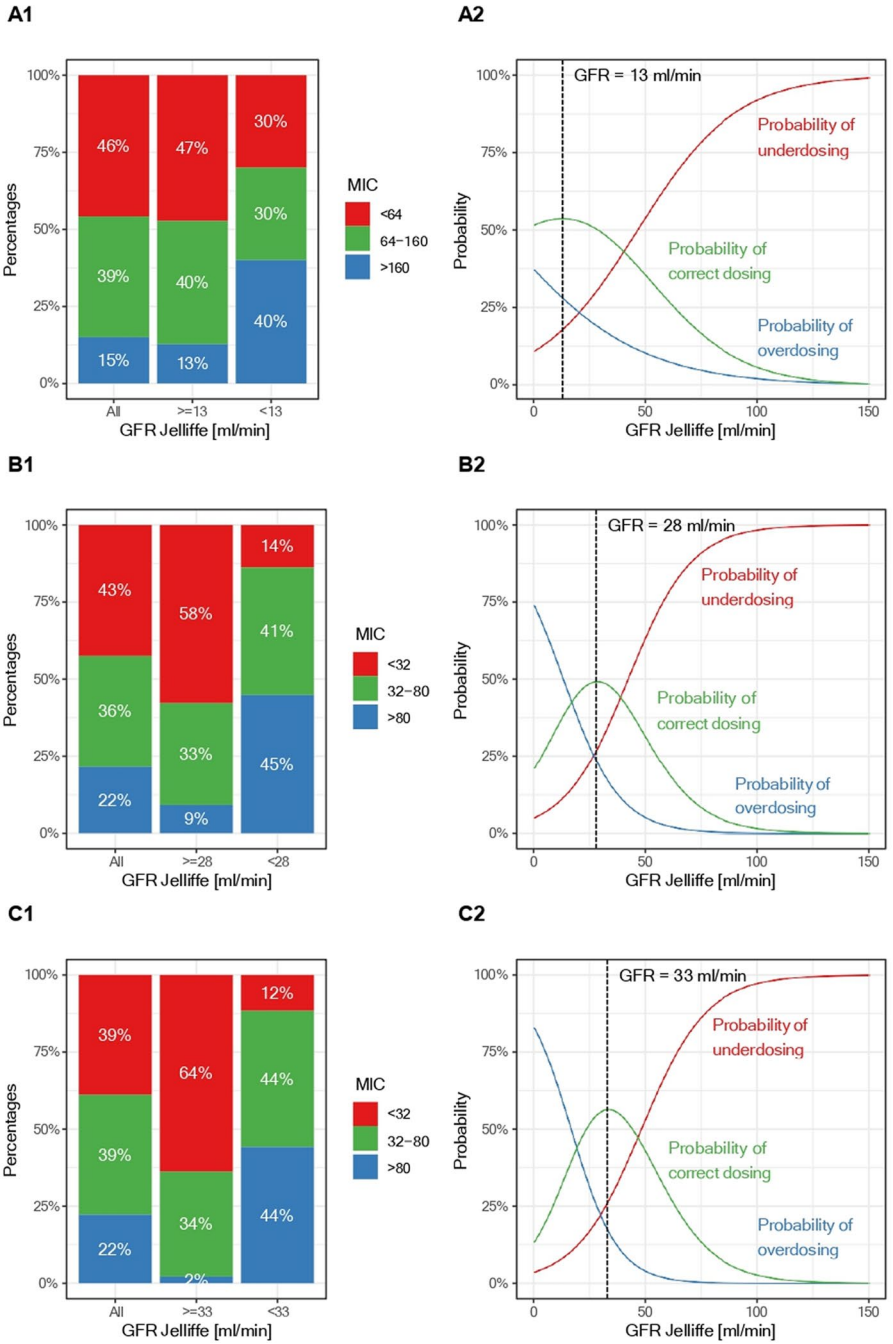
Attainment of therapeutic beta-lactam plasma levels targeting a Cmin of 4–10 × MIC measured in the entire study population of critically ill ICU patients (*N* = 377) treated with piperacillin–tazobactam, meropenem, or cefepime at doses considered to be full for the present indication, using the GFR Jelliffe equation. Exploratory data analysis for: **A1** – piperacillin–tazobactam, **B1** – meropenem, **C1** - cefepime, illustrating the proportion of patients (%) in each MIC-based exposure category (underdosing, target attainment, overdosing) across different categories of the GFR Jelliffe. Univariate logistic regression for: **A2** – piperacillin–tazobactam, **B2** – meropenem, **C2** – cefepime, illustrating the predicted probability of underdosing, correct dosing, and overdosing as a function of the GFR Jelliffe. Dashed vertical lines indicate GFR thresholds associated with optimal target attainment. Retrospective observational cohort study conducted at Na Homolce Hospital, Prague, Czech Republic (January 2019–December 2022) involving critically ill ICU patients (N = 377) treated with piperacillin–tazobactam, meropenem, or cefepime for acute infections. Antibiotic plasma trough concentrations were measured after 24 h of full-dose therapy using LC–MS/MS and compared with EUCAST breakpoints. The MIC values for piperacillin, meropenem, and cefepime were set at 16 mg/L, 8 mg/L, and 8 mg/L, respectively. Cmin – trough concentration, EUCAST – European Committee on Antimicrobial Susceptibility Testing, ICU – intensive care unit, MIC – minimum inhibitory concentration, LC–MS/MS – liquid chromatography tandem mass spectrometry, GFR Jelliffe – glomerular filtration rate estimated using the Jelliffe equation

**Fig. 7:**
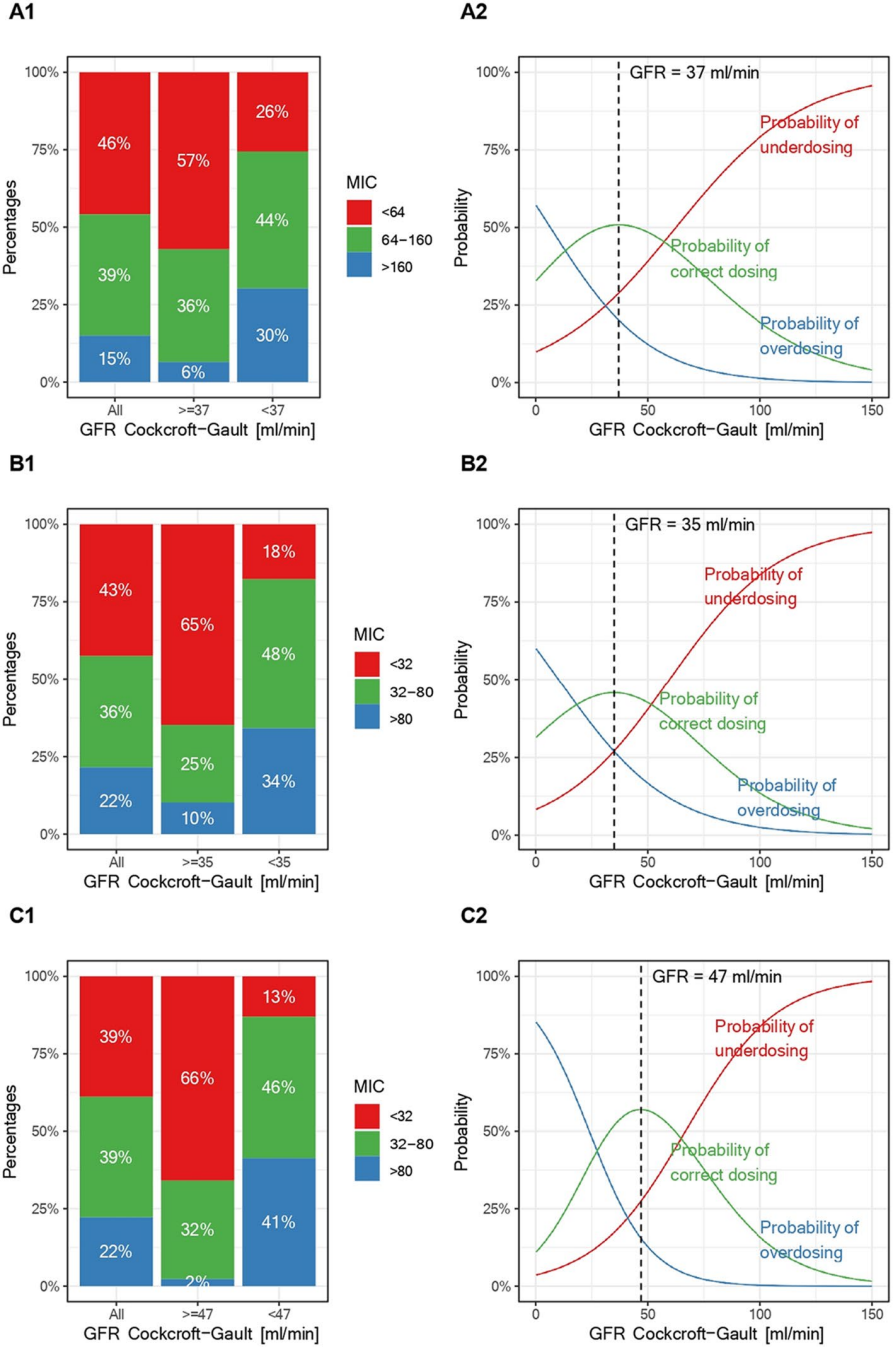
Attainment of therapeutic beta-lactam plasma levels targeting a Cmin of 4–10 × MIC measured in the entire study population of critically ill ICU patients (*N* = 377) treated with piperacillin–tazobactam, meropenem, or cefepime at doses considered to be full for the present indication, using the GFR Cockcroft-Gault equation. Exploratory data analysis for: **A1** – piperacillin–tazobactam, **B1** – meropenem, **C1** – cefepime, illustrating the proportion of patients (%) in each MIC-based exposure category (underdosing, target attainment, overdosing) across different categories of the GFR Cockcroft-Gault. Univariate logistic regression for: **A2** – piperacillin–tazobactam, **B2** – meropenem, **C2** – cefepime, illustrating the predicted probability of underdosing, correct dosing, and overdosing as a function of the GFR Cockcroft-Gault equation. Dashed vertical lines indicate GFR thresholds associated with optimal target attainment. Retrospective observational cohort study conducted at Na Homolce Hospital, Prague, Czech Republic (January 2019–December 2022) involving critically ill ICU patients (N = 377) treated with piperacillin–tazobactam, meropenem, or cefepime for acute infections. Antibiotic plasma trough concentrations were measured after 24 h of full-dose therapy using LC–MS/MS and compared with EUCAST breakpoints. The MIC values for piperacillin, meropenem, and cefepime were set at 16 mg/L, 8 mg/L, and 8 mg/L, respectively. Cmin – trough concentration, EUCAST – European Committee on Antimicrobial Susceptibility Testing, ICU – intensive care unit, MIC – minimum inhibitory concentration, LC–MS/MS - liquid chromatography tandem mass spectrometry, GFR Cockcroft-Gault equation – glomerular filtration rate estimated using the Cockcroft-Gault equation

**Fig. 8:**
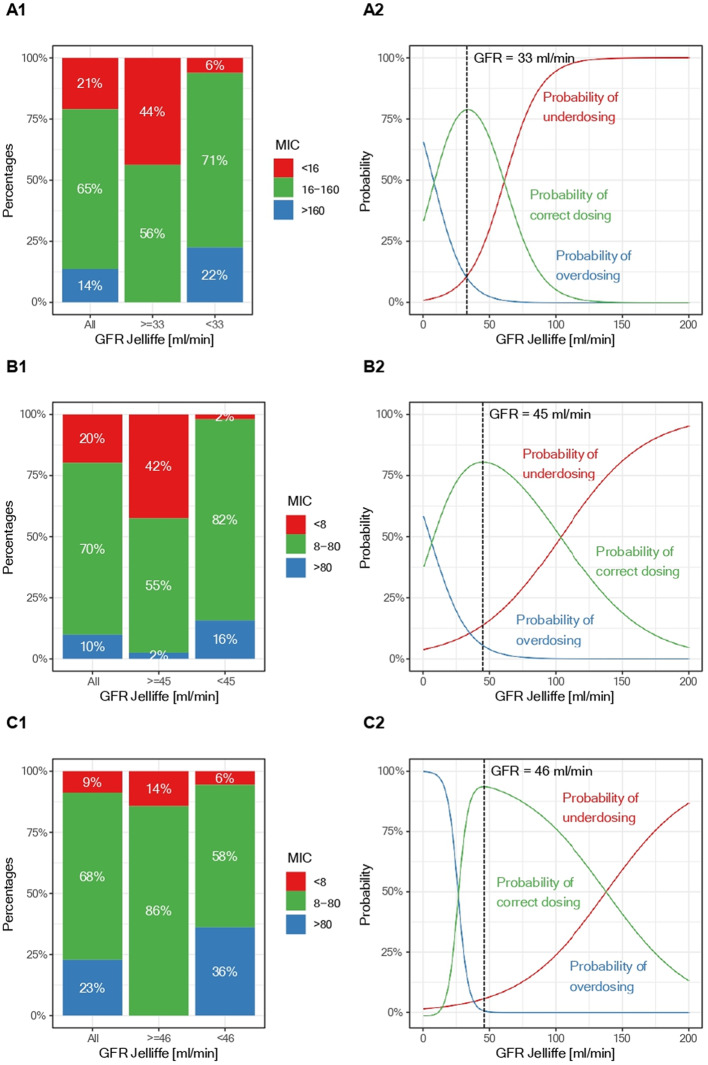
Attainment of therapeutic beta-lactam plasma levels targeting a Cmin of 1–10 × MIC in CD subpopulation treated with conventional maximum doses of beta-lactams: (piperacillin–tazobactam 4.5 g every 6 h, meropenem 2 g every 8 h, or cefepime 2 g every 8 h) (*N* = 229), using the GFR Jelliffe equation. Exploratory data analysis for: **A1** – piperacillin–tazobactam, **B1** – meropenem, **C1** – cefepime, illustrating the proportion of patients (%) in each MIC-based exposure category (underdosing, target attainment, overdosing) across different categories of the GFR Jelliffe. Univariate logistic regression for: **A2** – piperacillin–tazobactam, **B2** – meropenem, **C2** – cefepime, illustrating the predicted probability of underdosing, correct dosing, and overdosing as a function of the GFR Jelliffe. Dashed vertical lines indicate GFR thresholds associated with optimal target attainment. Retrospective observational cohort study conducted at Na Homolce Hospital, Prague, Czech Republic (January 2019–December 2022) involving critically ill ICU patients (N = 377) treated with piperacillin–tazobactam, meropenem, or cefepime for acute infections. Antibiotic plasma trough concentrations were measured after 24 h of full-dose therapy using LC–MS/MS and compared with EUCAST breakpoints. The MIC values for piperacillin, meropenem, and cefepime were set at 16 mg/L, 8 mg/L, and 8 mg/L, respectively. Cmin – trough concentration, EUCAST – European Committee on Antimicrobial Susceptibility Testing, ICU – intensive care unit, MIC – minimum inhibitory concentration, LC–MS/MS – liquid chromatography tandem mass spectrometry, CD patients – patients treated with conventional maximum doses (piperacillin–tazobactam 4.5 g every 6 h, meropenem 2 g every 8 h, or cefepime 2 g every 8 h), GFR Jelliffe – glomerular filtration rate estimated using the Jelliffe equation

**Fig. 9:**
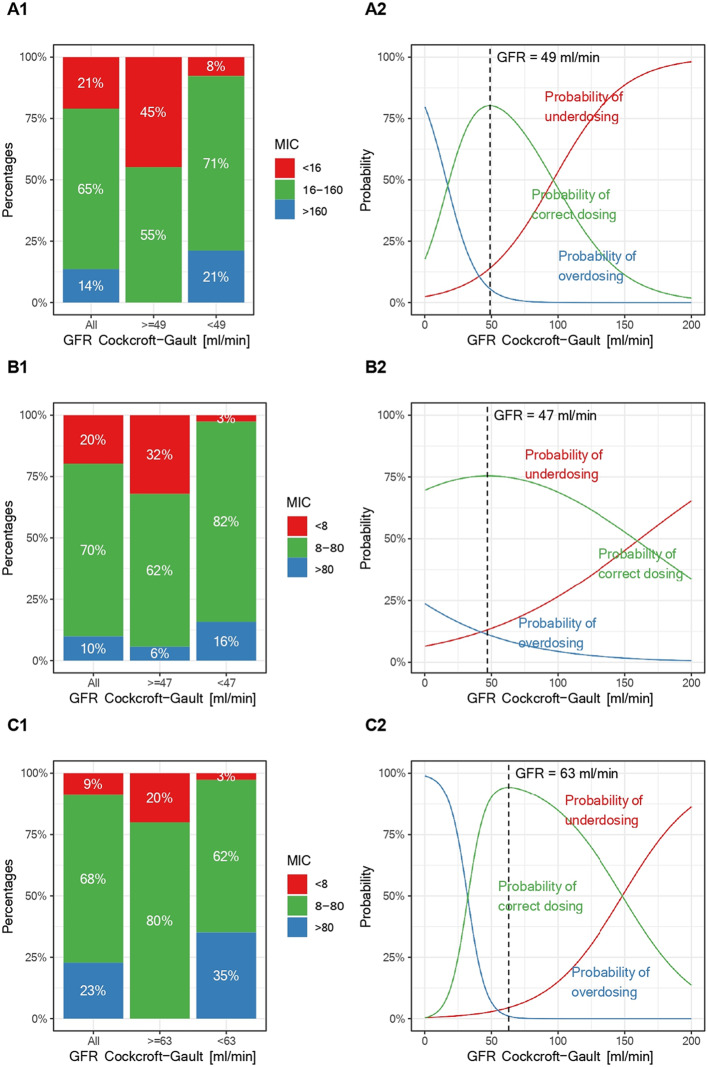
Attainment of therapeutic beta-lactam plasma levels targeting a Cmin of 1–10 × MIC in CD subpopulation treated with conventional maximum doses of beta-lactams: (piperacillin–tazobactam 4.5 g every 6 h, meropenem 2 g every 8 h, or cefepime 2 g every 8 h) (*N* = 229), using the GFR Cockcroft-Gault equation. Exploratory data analysis for: **A1** – piperacillin–tazobactam, **B1** – meropenem, **C1** – cefepime, illustrating the proportion of patients (%) in each MIC-based exposure category (underdosing, target attainment, overdosing) across different categories of the GFR Cockcroft-Gault. Univariate logistic regression for: **A2** – piperacillin–tazobactam, **B2** – meropenem, **C2** – cefepime, illustrating the predicted probability of underdosing, correct dosing, and overdosing as a function of the GFR Cockcroft-Gault. Dashed vertical lines indicate GFR thresholds associated with optimal target attainment. Retrospective observational cohort study conducted at Na Homolce Hospital, Prague, Czech Republic (January 2019–December 2022) involving critically ill ICU patients (N = 377) treated with piperacillin–tazobactam, meropenem, or cefepime for acute infections. Antibiotic plasma trough concentrations were measured after 24 h of full-dose therapy using LC–MS/MS and compared with EUCAST breakpoints. The MIC values for piperacillin, meropenem, and cefepime were set at 16 mg/L, 8 mg/L, and 8 mg/L, respectively. Cmin – trough concentration, EUCAST – European Committee on Antimicrobial Susceptibility Testing, ICU – intensive care unit, MIC – minimum inhibitory concentration, LC–MS/MS - liquid chromatography tandem mass spectrometry, CD patients – patients treated with conventional maximum doses (piperacillin–tazobactam 4.5 g every 6 h, meropenem 2 g every 8 h, or cefepime 2 g every 8 h), GFR Cockcroft-Gault – glomerular filtration rate estimated using the Cockcroft-Gault equation

**Fig. 10:**
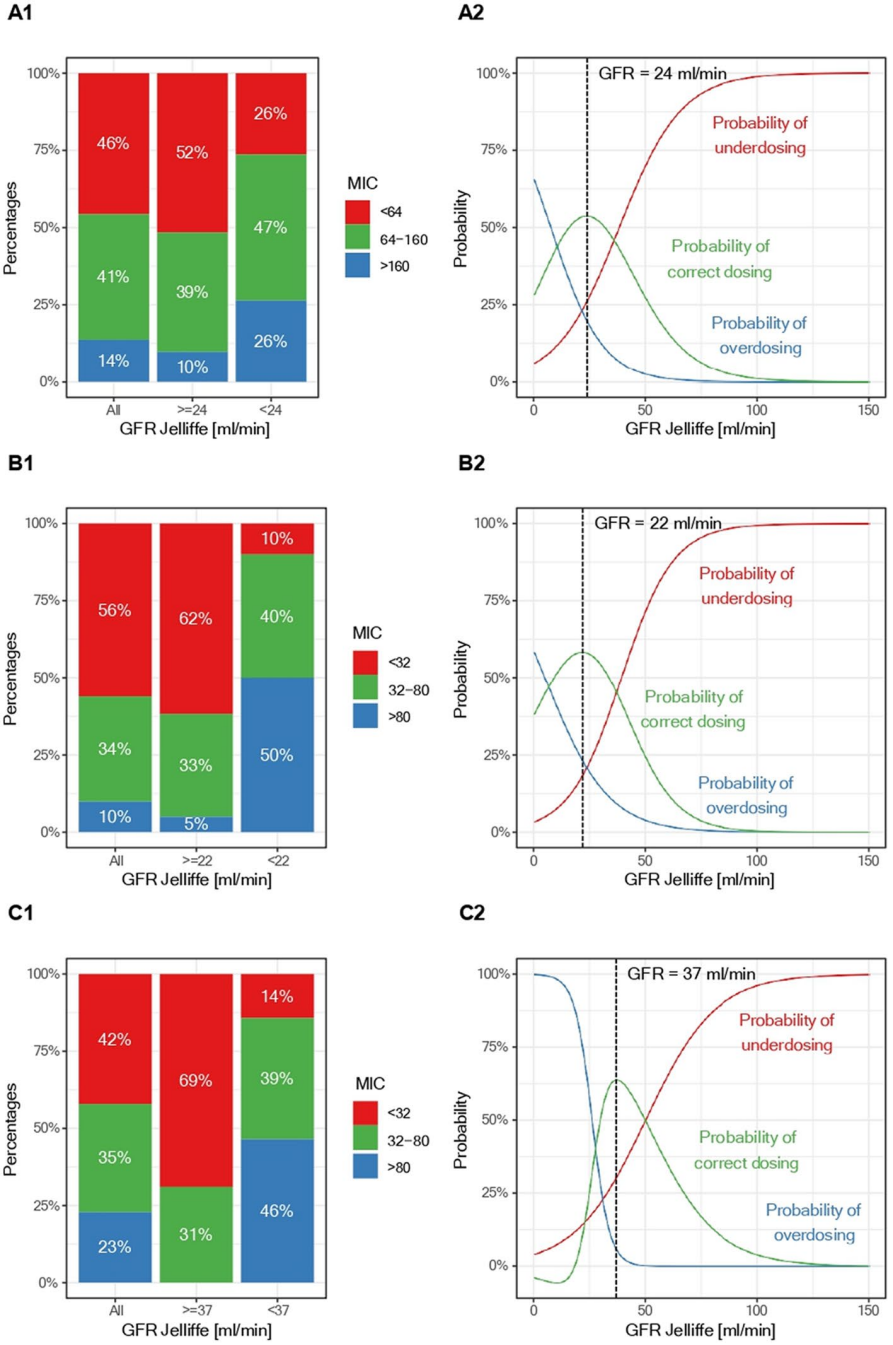
Attainment of therapeutic beta-lactam plasma levels targeting a Cmin of 4–10 × MIC in CD subpopulation treated with conventional maximum doses of beta-lactams: (piperacillin–tazobactam 4.5 g every 6 h, meropenem 2 g every 8 h, or cefepime 2 g every 8 h) (*N* = 229), using the GFR Jelliffe equation. Exploratory data analysis for: **A1** – piperacillin-tazobactam, **B1** – meropenem, **C1** – cefepime, illustrating the proportion of patients (%) in each MIC-based exposure category (underdosing, target attainment, overdosing) across different categories of the GFR Jelliffe. Univariate logistic regression for: **A2** – piperacillin–tazobactam, **B2** – meropenem, **C2** – cefepime, illustrating the predicted probability of underdosing, correct dosing, and overdosing as a function of the GFR Jelliffe. Dashed vertical lines indicate GFR thresholds associated with optimal target attainment. Retrospective observational cohort study conducted at Na Homolce Hospital, Prague, Czech Republic (January 2019–December 2022) involving critically ill ICU patients (N = 377) treated with piperacillin–tazobactam, meropenem, or cefepime for acute infections. Antibiotic plasma trough concentrations were measured after 24 h of full-dose therapy using LC–MS/MS and compared with EUCAST breakpoints. The MIC values for piperacillin, meropenem, and cefepime were set at 16 mg/L, 8 mg/L, and 8 mg/L, respectively. Cmin – trough concentration, EUCAST – European Committee on Antimicrobial Susceptibility Testing, ICU – intensive care unit, MIC – minimum inhibitory concentration, LC–MS/MS - liquid chromatography tandem mass spectrometry, CD patients – patients treated with conventional maximum doses (piperacillin–tazobactam 4.5 g every 6 h, meropenem 2 g every 8 h, or cefepime 2 g every 8 h), GFR Jelliffe – glomerular filtration rate estimated using the Jelliffe equation

**Fig. 11:**
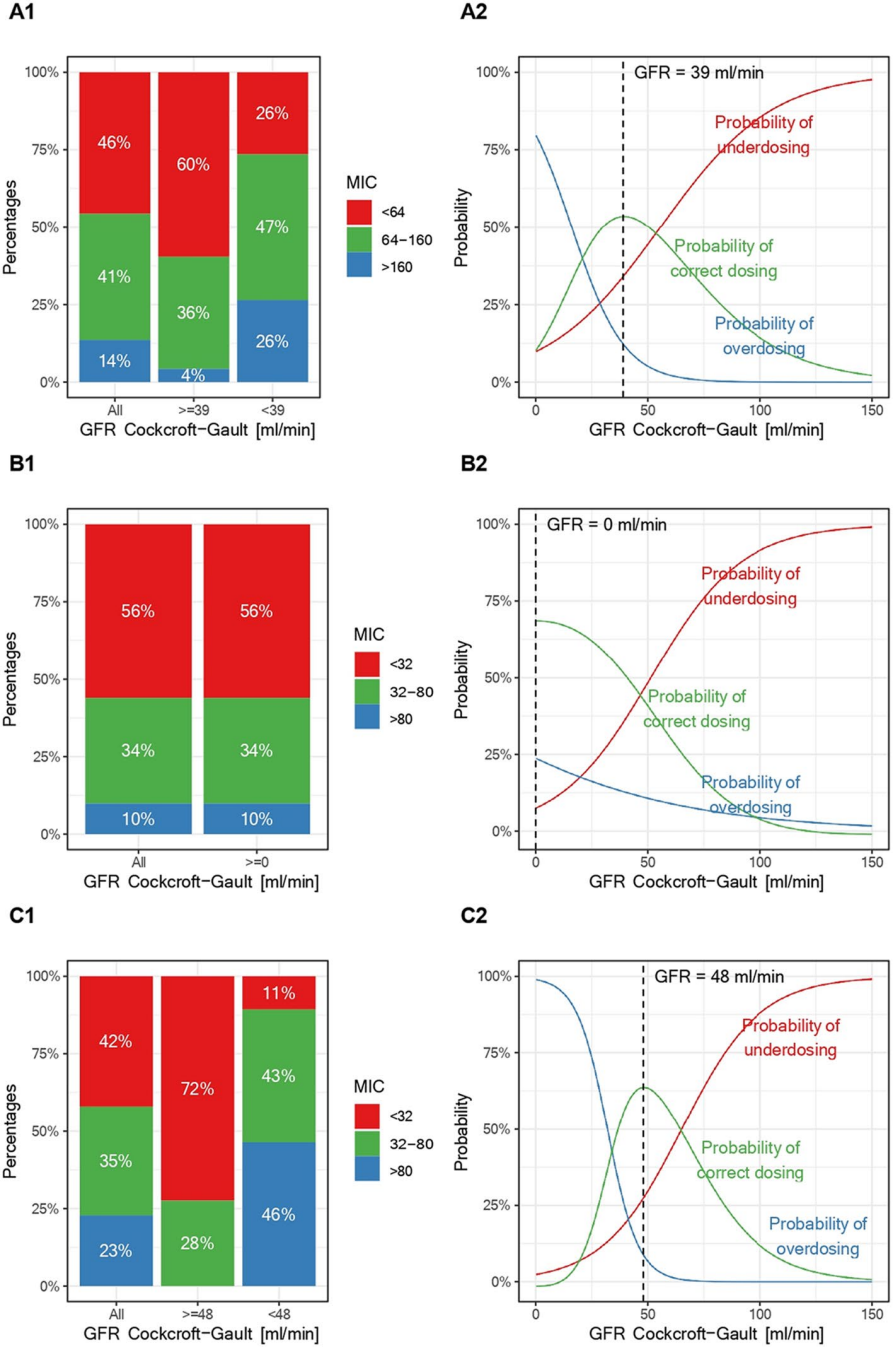
Attainment of therapeutic beta-lactam plasma levels targeting a Cmin of 1–10× MIC in CD subpopulation treated with conventional maximum doses of beta-lactams: (piperacillin–tazobactam 4.5 g every 6 h, meropenem 2 g every 8 h, or cefepime 2 g every 8 h) (*N* = 229), using the GFR Cockcroft-Gault equation. Exploratory data analysis for: **A1** – piperacillin–tazobactam, **B1** – meropenem, **C1** – cefepime, illustrating the proportion of patients (%) in each MIC-based exposure category (underdosing, target attainment, overdosing) across different categories of the GFR Cockcroft-Gault. Univariate logistic regression for: **A2** – piperacillin–tazobactam, **B2** – meropenem, **C2** – cefepime, illustrating the predicted probability of underdosing, correct dosing, and overdosing as a function of the GFR Cockcroft-Gault. Dashed vertical lines indicate GFR thresholds associated with optimal target attainment. Retrospective observational cohort study conducted at Na Homolce Hospital, Prague, Czech Republic (January 2019–December 2022) involving critically ill ICU patients (N = 377) treated with piperacillin–tazobactam, meropenem, or cefepime for acute infections. Antibiotic plasma trough concentrations were measured after 24 h of full-dose therapy using LC–MS/MS and compared with EUCAST breakpoints. The MIC values for piperacillin, meropenem, and cefepime were set at 16 mg/L, 8 mg/L, and 8 mg/L, respectively. Cmin – trough concentration, EUCAST – European Committee on Antimicrobial Susceptibility Testing, ICU – intensive care unit, MIC – minimum inhibitory concentration, LC–MS/MS – liquid chromatography tandem mass spectrometry, CD patients – patients treated with conventional maximum doses (piperacillin–tazobactam 4.5 g every 6 h, meropenem 2 g every 8 h, or cefepime 2 g every 8 h), GFR Cockcroft-Gault – glomerular filtration rate estimated using the Cockcroft-Gault equation

Tables S9, S10, and S11 show the results of stratification by the number of doses received within 24 h (i.e., 3, 4, or 6 doses). Across these analyses, GFR consistently emerged as the only independent predictor of achieved plasma concentrations, with the exception of the every-4-hour dosing regimen in patients with supratherapeutic plasma levels; however, this subgroup was limited in size (*N* = 35). Figures 4, 5, 6, 7, 8, 9, 10 and 11 demonstrate the achieved plasma levels for the different molecules and targets.

No adverse cases of organ toxicity, including neurotoxicity, hematotoxicity, or hepatotoxicity, were observed during the entire course of antibiotic treatment. The overall incidence of acute kidney injury (AKI) decreased over the treatment period, from 60.2% at initiation to 49% at the end of therapy. At no point was AKI considered by the treating clinicians to be potentially attributable to antibiotic therapy.

## Discussion

Our results show substantial differences in the proportion of critically ill patients achieving the required beta-lactam trough concentration targets (meropenem, piperacillin–tazobactam, or cefepime), depending on the selected pharmacokinetic/pharmacodynamic threshold. When targeting a Cmin of 4–10 × MIC, 42.7% of patients (36.7% in the CD subpopulation) failed to reach the target range, whereas only 13% of patients (17.5% in the CD subpopulation) were below target when aiming for a Cmin of 1–10 × MIC. The high proportion of underdosed patients, despite the administration of full beta-lactam doses, suggests that the more stringent target of 4–10 × MIC may be too unrealistic to achieve. This observation is consistent with other authors who generally accept 100% fT > MIC as a prudent PK/PD target in critically ill ICU patients [15]. This target appears to be achievable in most ICU patients treated with full beta-lactam doses, and some authors have suggested its clinical non-inferiority and possibly even safety superiority compared with Cmin = 4–10 × MIC [8]. However, robust prospective data from randomized controlled trials evaluating clinical outcomes associated with different target attainment strategies are lacking, and further clinical research in this field is therefore warranted.

In the overall population, 19.6% of patients (*N* = 74) and 14.4% (*N* = 33) in the CD subpopulation were identified as having supratherapeutic plasma concentrations. The large interindividual variability in plasma concentrations observed in this study is consistent with previous reports [16–18], in which a “first dose fits all” approach similarly resulted in suboptimal pharmacokinetic outcomes. Although the proportion of overdosed patients in our study was not negligible, and some authors have proposed even lower thresholds as maximum safe concentrations, we did not observe any clinically significant adverse events, including neurotoxicity, hematotoxicity, or hepatotoxicity, assessed by the treating physician as potentially causally related to the antibiotic therapy. This finding is likely attributable to subsequent dose adjustment based on renal function [19, 20, 21]. Regarding renal outcomes, a high proportion of patients (over 50%) presented with acute kidney injury (AKI) at the initiation of antibiotic therapy, consistent with previously reported AKI rates in ICU populations [22, 23]. The etiology of AKI in critically ill patients is usually considered multifactorial, including underlying sepsis, hemodynamic instability, and exposure to various nephrotoxic insults. Importantly, in none of the cases was β-lactam therapy considered a potential causal factor by the treating clinicians. On the contrary, the overall incidence of AKI decreased slightly over the treatment course, most likely reflecting resolution of sepsis and overall clinical improvement. These observations support the interpretation that the high prevalence of AKI in our cohort reflects the underlying ICU setting rather than β-lactam–associated nephrotoxicity. Therefore, we believe that our approach—treating with full therapeutic doses for the first 24 h despite renal impairment, followed by subsequent dose adjustment—can be considered prudent and is also supported by other authors [24, 25].

Of all the parameters analyzed, the only consistent and independent predictor across both the entire population and the CD subpopulation was GFR. These findings are consistent with previous reports identifying renal impairment as a crucial covariate influencing beta-lactam plasma levels in critically ill patients [26]. Interestingly, despite more severe renal impairment (defined as GFR < 0.8 mL/s, i.e., 48 mL/min), this subgroup still included approximately 20% of patients—both in the entire population and the CD subpopulation—who failed to achieve the 1–10 × MIC target, and approximately 30% patients who remained underdosed when applying the 4–10 × MIC threshold. A plausible explanation for these findings is the pharmacokinetic alterations associated with AKI, especially reduced drug clearance and prolonged half-life, which may delay attainment of steady-state concentrations [27]. However, a higher proportion of patients with renal impairment were overexposed after 24 h of full therapy, highlighting the difficulty of predicting β-lactam pharmacokinetics in ICU patients and the importance of TDM of β-lactam antibiotics in this setting. A small proportion of patients continued to be underdosed even after adjustment for a less stringent target, suggesting that in some critically ill patients (e.g., those with hyperfiltration), it may be reasonable to increase the initial dose above the normally recommended maximum. However, this strategy requires further investigation.

Our study has several limitations. First, due to the unavailability of MIC values during the initial hours of treatment, we related plasma concentrations to EUCAST MIC values [13]. Our assumptions describe a worst-case scenario, but this approach is appropriate for empirical dose selection. If more susceptible bacteria had been causing the infection, the PK/PD ratios would have been higher than those reported here. Second, the measured plasma concentrations may not fully reflect drug levels at the site of infection. However, blood is generally a suitable sample, as it is expected to reflect concentrations similar to those at the target site of infection in most subjects, except when the causative agent is an intracellular pathogen or the infectious focus is behind an impenetrable barrier (e.g., the central nervous system or the prostate). Third, diagnosing neurotoxicity in the intensive care unit is challenging due to the use of sedatives and antipsychotics, and electroencephalogram monitoring was not routinely available. Nonetheless, clinically significant manifestations of neurotoxicity (tonic-clonic spasms, myoclonus, and orofacial dyskinesia) can still be identified despite the effect of analgesia, which is usually light in contemporary ICU practice. Fourth, the correlation of PK/PD target attainment with clinical outcomes was not an objective of the present study due to its retrospective nature and difficulty in obtaining complete outcome data. For example, commonly used scoring systems to assess disease severity and estimate ICU mortality (SOFA, APACHE II) rely on multiple laboratory values and patient signs, many of which are not routinely recorded and thus cannot be extracted retrospectively from medical records. Fifth, increasing evidence suggests that prolonged or continuous infusion of beta-lactams may improve PK/PD target attainment and clinical outcomes, potentially reducing the proportion of patients with subtherapeutic plasma concentrations. However, at the time this study was initiated, prolonged or continuous infusion regimens were used only in select patients on an individualized basis, rather than universally; therefore, we cannot provide comparative data between these regimens. Nonetheless, we believe that a prospective, head-to-head comparison of intermittent versus prolonged/continuous infusion would be of high clinical importance. Finally, the single-center design of the present study may limit the generalizability of our findings, highlighting the need for further prospective, multicenter validation.

In conclusion, targeting a Cmin = 4–10 × MIC results in a high proportion of patients remaining underdosed despite receiving full (maximum) beta-lactam doses, whereas the 1–10 × MIC target is achieved in the vast majority of patients. GFR is an independent predictor of beta-lactam plasma concentration attainment. Our results highlight the importance of therapeutic drug monitoring of beta-lactam antibiotics in critically ill patients and support the need for further research in this field.

## Supplementary Information

Below is the link to the electronic supplementary material.

Supplementary Material 1

## Data Availability

The datasets are available from the corresponding author upon reasonable request.
