# Supplementary material for: Achieving target plasma concentrations of beta-lactam antibiotics in critically ill patients: a retrospective study of full-dose administration in the first 24 hours
Source: Pharmacol Rep. 2026 Feb 25;78(3):929–44. doi: 10.1007/s43440-026-00836-8 (PMC13275785; doi:10.1007/s43440-026-00836-8)
Supplement: Supplementary file 1 — Supplementary Material 1 [file 43440_2026_836_MOESM1_ESM.docx]

**Achieving Target Plasma Concentrations of Beta-Lactam Antibiotics in Critically Ill Patients: A Retrospective Analysis of Full-Dose Administration for first 24 hours.**

**Supplemental Appendix**

**Content:**

**All patients**

**Table S1** (a-c): Baseline characteristics and dosage regimen analysis………….….……..2

**Table S2** (a-e): The achieved plasma concentrations and factors associated with underdosing for Cmin = 1-10x MIC…………………………………………………….6

**Table S3** (a-e): The achieved plasma concentrations and factors associated with underdosing for Cmin = 4-10x MIC…………………………………………………………..9

**Table S4** (a-d): Factors associated with overdosing (Cmin > 10x MIC)…………………13

**Subanalysis of patients treated with conventional doses (CD patients)**

**Table S5** (a-b)**:** Baseline characteristics ……………………………………………….16

**Table S6** (a-e): The achieved plasma concentrations and factors associated with underdosing for Cmin = 1-10x MIC……………………………………………..…………18

**Table S7** (a-e): The achieved plasma concentrations and factors associated with underdosing and for Cmin = 4-10x MIC………………………………………………..…23

**Table S8** (a-d): Factors associated with overdosing (Cmin > 10x MIC)………………..28

**Results stratification according to dose number at time of level collection**

**Table S9** (a-j) Dose regimen every 8 hours (i.e. 3 doses/24 hours)……………………..…31

**Table S10** (a-j) Dose regimen every 6 hours (i.e. 4 doses/24 hours)……………………..40

**Table S11** (a-j) Dose regimen every 4 hours (i.e. 6 doses/24 hours)………………………..48

**1 All patients**

**Table S1 (a-c):** Baseline characteristics and dosage regimen analysis

1a Categorical variables

| **Characteristic** | **N = 377** |  |
| --- | --- | --- |
| Antibiotic |  |  |
| Cefepime | 90.0 (23.9%) |  |
| Meropenem | 167.0 (44.3%) |  |
| Piperacillin/ tazobactam | 120.0 (31.8%) |  |
| Sex |  |  |
| F | 107.0 (28.4%) |  |
| M | 270.0 (71.6%) |  |
| Vasopressor  none | 172.0 (45.6%) |  |
| low | 98.0 (26.0%) |  |
| medium | 66.0 (17.5%) |  |
| high | 41.0 (10.9%) |  |
| AKI |  |  |
| No | 150.0 (39.8%) |  |
| Yes | 227.0 (60.2%) |  |
| CRRT |  |  |
| No | 305.0 (80.9%) |  |
| Yes | 72.0 (19.1%) |  |
| GFR_Jelliffe |  |  |
| >120 | 15.0 (4.0%) |  |
| 48–120 | 97.0 (25.7%) |  |
| 12–48 | 166.0 (44.0%) |  |
| <12 | 27.0 (7.2%) |  |
| CRRT | 72.0 (19.1%) |  |
| GFR_CG |  |  |
| >120 | 25.0 (6.6%) |  |
| 48–120 | 143.0 (37.9%) |  |
| 12–48 | 131.0 (34.7%) |  |
| <12 | 6.0 (1.6%) |  |
| CRRT | 72.0 (19.1%) |  |
| ECMO  No | 368.0 (97.6%) |  |
| Yes | 9.0 (2.4%) |  |
| 30 day mortality |  |  |
| No | 298.0 (79.0%) |  |
| Yes | 79.0 (21.0%) |  |
| Indication  CNS | 20.0 (5.3%) |  |
| DMT | 39.0 (10.3%) |  |
| GIT | 33.0 (8.8%) |  |
| HCD | 7.0 (1.9%) |  |
| CVS | 54.0 (14.3%) |  |
| OTH | 9.0 (2.4%) |  |
| PNE | 115.0 (30.5%) |  |
| SEPSIS un | 54.0 (14.3%) |  |
| TRB | 37.0 (9.8%) |  |
| URO | 9.0 (2.4%) |  |
| Sepsis |  |  |
| No | 280.0 (74.3%) |  |
| Yes | 97.0 (25.7%) |  |
| This retrospective observational cohort study was conducted at Na Homolce Hospital, Prague, Czech Republic (January 2019–December 2022). We included consecutive critically ill ICU patients (N = 377) treated with meropenem, cefepime, or piperacillin–tazobactam for acute infections. Antibiotic plasma trough concentrations (Cmin) were measured after 24 h of full dosing using LC–MS/MS. Antibiotic concentrations were related to breakpoints obtained from the European Committee on Antimicrobial Susceptibility Testing (EUCAST). The MIC values for meropenem, cefepime and piperacillin were set at 8 mg/l, 8 mg/l, and 16 mg/l, respectively. Data are presented as absolute and relative frequencies. BMI – Body mass index; GFR Jelliffe – glomerular filtration rate estimated using the Jelliffe equation, GFR_CG – glomerular filtration rate estimated using Cockcroft-Gault equation, AKI –a acute kidney injury, ECMO - Extracorporeal membrane oxygenation, CRRT – continuous renal replacement therapy, CNS- central nervous infection, SIN-sinusitis, DMT-soft tissue infections, GIT-abdominal infections, CVS-cardiovascular infections, PNE-pneumonias, TRB-tracheobronchitis, URO-uroinfections, SEPSIS un-sepsis of unknown origin, OTH-other | | |

1b Continuous variables

| variable | mean | sd | p25 | p50 | p75 | min | max |
| --- | --- | --- | --- | --- | --- | --- | --- |
| Age | 67.1 | 12.6 | 62.0 | 70.0 | 74.0 | 22.0 | 92.0 |
| Weight | 87.5 | 19.4 | 75.0 | 87.0 | 97.0 | 36.0 | 180.0 |
| Height | 173.4 | 9.9 | 167.0 | 173.0 | 180.0 | 149.0 | 205.0 |
| BMI | 29.1 | 6.0 | 24.9 | 28.4 | 32.4 | 15.4 | 62.3 |
| GFR_Jelliffe | 43.0 | 34.7 | 23.8 | 30.0 | 52.8 | 3.5 | 372.4 |
| lnGFR_Jelliffe | 3.5 | 0.7 | 3.2 | 3.4 | 4.0 | 1.3 | 5.9 |
| GFR_CG | 57.6 | 43.8 | 30.0 | 43.5 | 76.6 | 4.9 | 404.2 |
| lnGFR_CG | 3.8 | 0.6 | 3.4 | 3.8 | 4.3 | 1.6 | 6.0 |
| This retrospective observational cohort study was conducted at Na Homolce Hospital, Prague, Czech Republic (January 2019–December 2022). We included consecutive critically ill ICU patients (N = 377) treated with meropenem, cefepime, or piperacillin–tazobactam for acute infections. Antibiotic plasma trough concentrations (Cmin) were measured after 24 h of full dosing using LC–MS/MS. Antibiotic concentrations were related to breakpoints obtained from the European Committee on Antimicrobial Susceptibility Testing (EUCAST). The MIC values for meropenem, cefepime and piperacillin were set at 8 mg/l, 8 mg/l, and 16 mg/l, respectively. Data are presented as mean, standard deviation (SD), 25th percentile (P25), median (P50), 75th percentile (P75), minimum, and maximum values. Sample size N= 377. BMI - Body mass index; GFR Jelliffe – glomerular filtration rate estimated using the Jelliffe equation, GFR_CG – glomerular filtration rate estimated using Cockcroft-Gault equation, sd- standard deviation, p - percentil | | | | | | | |

1c Distribution of beta-lactams doses in the study (N=377)

Piperacillin Tazobactam doses (N= 120)

| Characteristic | N = 120 (%) |
| --- | --- |
| Dose | N = 120 (%) |
| 4.5 | 120.0 (100.0%) |
| Interval |  |
| 4 | 39.0 (32.5%) |
| 6 | 77.0 (64.1%) |
| 8 | 4.0 (3.3%) |
| Dose_daily |  |
| 13.5 | 4.0 (3.3%) |
| 18 | 77.0 (64.1%) |
| 27 | 39.0 (32.5%) |
| This retrospective observational cohort study was conducted at Na Homolce Hospital, Prague, Czech Republic (January 2019–December 2022). We included consecutive critically ill ICU patients (N = 377) treated with meropenem, cefepime, or piperacillin–tazobactam for acute infections. Antibiotic plasma trough concentrations (Cmin) were measured after 24 h of full dosing using LC–MS/MS. Antibiotic concentrations were related to breakpoints obtained from the European Committee on Antimicrobial Susceptibility Testing (EUCAST). The MIC values for meropenem, cefepime and piperacillin were set at 8 mg/l, 8 mg/l, and 16 mg/l, respectively.Data are presented as absolute and relative frequencies. | |

Meropenem doses (N = 167)

| Characteristic | N = 167 (%) |
| --- | --- |
| Dose |  |
| 1 | 71.0 (42.5%) |
| 2 | 96.0 (57.5%) |
| Interval |  |
| 6 | 62.0 (38.3%) |
| 8 | 105.0 (61.7%) |
| Dose_daily |  |
| 3 | 9.0 (5.4%) |
| 4 | 62.0 (37.1%) |
| 6 | 96.0 (56.3%) |
| 8 | 2.0 (1.2%) |
| This retrospective observational cohort study was conducted at Na Homolce Hospital, Prague, Czech Republic (January 2019–December 2022). We included consecutive critically ill ICU patients (N = 377) treated with meropenem, cefepime, or piperacillin–tazobactam for acute infections. Antibiotic plasma trough concentrations (Cmin) were measured after 24 h of full dosing using LC–MS/MS. Antibiotic concentrations were related to breakpoints obtained from the European Committee on Antimicrobial Susceptibility Testing (EUCAST). The MIC values for meropenem, cefepime and piperacillin were set at 8 mg/l, 8 mg/l, and 16 mg/l, respectively.Data are presented as absolute and relative frequencies. | |

Cefepime doses (N = 90)

| Characteristic | N = 90 (%) |
| --- | --- |
| Dose |  |
| 1 | 29.0 (32.2%) |
| 2 | 58.0 (64.4%) |
| 3 | 3.0 (3.3%) |
| Interval |  |
| 6 | 14.0 (15.6%) |
| 8 | 76.0 (84.4%) |
| Dose_daily |  |
| 3 | 16.0 (17.8%) |
| 4 | 13.0 (14.4%) |
| 6 | 56.0 (62.2%) |
| 8 | 5.0 (5.5%) |
| This retrospective observational cohort study was conducted at Na Homolce Hospital, Prague, Czech Republic (January 2019–December 2022). We included consecutive critically ill ICU patients (N = 377) treated with meropenem, cefepime, or piperacillin–tazobactam for acute infections. Antibiotic plasma trough concentrations (Cmin) were measured after 24 h of full dosing using LC–MS/MS. Antibiotic concentrations were related to breakpoints obtained from the European Committee on Antimicrobial Susceptibility Testing (EUCAST). The MIC values for meropenem, cefepime and piperacillin were set at 8 mg/l, 8 mg/l, and 16 mg/l, respectively. Data are presented as absolute and relative frequencies. | |

**Table S2 (a-e):** The achieved plasma concentrations and factors associated with underdosing for Cmin = 1-10x MIC

2a Achieved plasma concentrations (Cmin = 1-10x MIC): All patients

| MIC | N | (%) |
| --- | --- | --- |
| 1-10 | 253 | 67.1% |
| >10 | 74 | 19.6% |
| <1 | 50 | 13.3% |
| This retrospective observational cohort study was conducted at Na Homolce Hospital, Prague, Czech Republic (January 2019–December 2022). We included consecutive critically ill ICU patients (N = 377) treated with meropenem, cefepime, or piperacillin–tazobactam for acute infections. Antibiotic plasma trough concentrations (Cmin) were measured after 24 h of full dosing using LC–MS/MS. Antibiotic concentrations were related to breakpoints obtained from the European Committee on Antimicrobial Susceptibility Testing (EUCAST). The MIC values for meropenem, cefepime and piperacillin were set at 8 mg/l, 8 mg/l, and 16 mg/l, respectively.Data are presented as absolute and relative frequencies. Sample size N= 377. MIC – minimum inhibitory concentration | | |

2b Factors associated with underdosing (Cmin < 1x MIC): All patients. Exploratory analysis

| **Characteristic** | | **N** | **<1**  N = 50 | **>=1**  N = 327 | | **Test statistic (df/N)** | **p-value** | |
| --- | --- | --- | --- | --- | --- | --- | --- | --- |
| Age | | 377 | 61 (53, 68) | 71 (64, 75) | | U=4286.5; N=50/327 | <0.001^1^ | |
| Sex | | 377 |  |  | | χ²=0.16; df=1 | 0.739^2^ | |
| F | |  | 13.0 (26.0%) | 94.0 (28.7%) | |  |  | |
| M | |  | 37.0 (74.0%) | 233.0 (71.3%) | |  |  | |
| Weight | | 377 | 85 (75, 95) | 87 (75, 97) | | U=8134.5; N=50/327 | 0.956^1^ | |
| Height | | 377 | 180 (168, 185) | 172 (167, 180) | | U=9730; N=50/327 | 0.030^1^ | |
| BMI | | 377 | 27.8 (24.7, 32.1) | 28.6 (25.3, 32.4) | | U=7135; N=50/327 | 0.147^1^ | |
| Vasopressor | | 377 |  |  | | χ²=13.15; df=3 | 0.004^2^ | |
| none | | 34.0 (68.0%) | |  | 138.0 (42.2%) | | | |
| low | | 10.0 (20.0%) | |  | 88.0 (26.9%) | | | |
| medium | | 5.0 (10.0%) | |  | 61.0 (18.7%) | | | |
| high | | 1.0 (2.0%) | |  | 40.0 (12.2%) | | | |
| GFR_Jelliffe | | 377 | 67 (50, 95) | 30 (21, 45) | | U=13647; N=50/327 | | <0.001^1^ |
| GFR_Jelliffe | | 377 |  |  | |  | | <0.001^3^ |
| >120 | |  | 8.0 (16.0%) | 7.0 (2.1%) | |  | |  |
| 48–120 | |  | 30.0 (60.0%) | 67.0 (20.5%) | |  | |  |
| 12–48 | |  | 10.0 (20.0%) | 156.0 (47.7%) | |  | |  |
| <12 | |  | 0.0 (0.0%) | 27.0 (8.3%) | |  | |  |
| GFR_CG | | 377 | 91 (61, 115) | 39 (30, 65) | | U=13156.5; N=50/327 | | <0.001^1^ |
| GFR_CG | | 377 |  |  | |  | | <0.001^3^ |
| >120 | |  | 12.0 (24.0%) | 13.0 (4.0%) | |  | |  |
| 48–120 | |  | 31.0 (62.0%) | 112.0 (34.3%) | |  | |  |
| 12–48 | |  | 5.0 (10.0%) | 126.0 (38.5%) | |  | |  |
| <12 | |  | 0.0 (0.0%) | 6.0 (1.8%) | |  | |  |
| CRRT | | 377 |  |  | | χ²=8.5; df=1 | | 0.002^2^ |
| No | | 48.0 (96.0%) | |  | 257.0 (78.6%) | | | |
| Yes | | 2.0 (4.0%) | |  | 70.0 (21.4%) | | | |
| AKI | | 377 | 8.0 (16.0%) | 219.0 (67.0%) | | χ²=47.03; df=1 | <0.001^2^ | |
|  | This retrospective observational cohort study was conducted at Na Homolce Hospital, Prague, Czech Republic (January 2019–December 2022). We included consecutive critically ill ICU patients (N = 377) treated with meropenem, cefepime, or piperacillin–tazobactam for acute infections. Antibiotic plasma trough concentrations (Cmin) were measured after 24 h of full dosing using LC–MS/MS. Antibiotic concentrations were related to breakpoints obtained from the European Committee on Antimicrobial Susceptibility Testing (EUCAST). The MIC values for meropenem, cefepime and piperacillin were set at 8 mg/l, 8 mg/l, and 16 mg/l, respectively. Categorical variables presented as N (%). Continuous variables presented as median (Q1, Q3). BMI - Body mass index; GFR Jelliffe – glomerular filtration rate estimated using the Jelliffe equation, GFR_CG – glomerular filtration rate estimated using Cockcroft-Gault equation, CRRT – continuous renal replacement therapy, AKI - acute kidney injury  1 Mann–Whitney U test; ^2^ Chi-squared test; ^3^Fisher’s exact test | | | | | | | |

2c Factors associated with underdosing (Cmin < 1x MIC): All patients. Univariate logistic regression

| **Characteristic** | **OR** | **95% CI** | **p-value** |
| --- | --- | --- | --- |
| Age | 1.06 | 1.03, 1.08 | <0.001 |
| Sex |  |  |  |
| F | — | — |  |
| M | 0.87 | 0.43, 1.67 | 0.688 |
| Weight | 1.00 | 0.99, 1.02 | 0.841 |
| Height | 0.96 | 0.93, 0.99 | 0.008 |
| BMI | 1.05 | 0.99, 1.11 | 0.101 |
| Vasopressor |  |  |  |
| none low | — 2.17 | — 1.05, 4.83 | 0.044 |
| medium | 3.01 | 1.22, 9.09 | 0.029 |
| high | 9.86 | 2.02, 178 | 0.026 |
| GFR_Jelliffe | 0.97 | 0.96, 0.98 | <0.001 |
| GFR_CG | 0.98 | 0.97, 0.98 | <0.001 |
| CRRT |  |  |  |
| No | — | — |  |
| Yes | 6.54 | 1.96, 40.6 | 0.011 |
| AKI |  |  |  |
| No | — | — |  |
| Yes | 10.6 | 5.08, 25.2 | <0.001 |
| This retrospective observational cohort study was conducted at Na Homolce Hospital, Prague, Czech Republic (January 2019–December 2022). We included consecutive critically ill ICU patients (N = 377) treated with meropenem, cefepime, or piperacillin–tazobactam for acute infections. Antibiotic plasma trough concentrations (Cmin) were measured after 24 h of full dosing using LC–MS/MS. Antibiotic concentrations were related to breakpoints obtained from the European Committee on Antimicrobial Susceptibility Testing (EUCAST). The MIC values for meropenem, cefepime and piperacillin were set at 8 mg/l, 8 mg/l, and 16 mg/l, respectively. Sample size N= 377. Univariate logistic regression. Data are presented as OR (odds ratio), 95 % CI (confidence interval) and p-value. BMI - Body mass index; GFR Jelliffe – glomerular filtration rate estimated using the Jelliffe equation, GFR_CG – glomerular filtration rate estimated using Cockcroft-Gault equation, CRRT – continuous renal replacement therapy, AKI - acute kidney injury, CI = Confidence Interval, OR = Odds Ratio | | | |

2d Factors associated with underdosing (Cmin < 1x MIC), GFR estimated using the Jelliffe equation: All patients. Multivariate logistic regression^1^

| Characteristic | OR | Conf.low | Conf.high | p-value |
| --- | --- | --- | --- | --- |
| Age | 1.025 | 0.992 | 1.057 | 0.134 |
| Sex M | 0.835 | 0.355 | 1.838 | 0.663 |
| BMI | 1.059 | 0.995 | 1.132 | 0.073 |
| Vasopressor |  |  |  | 0.109 |
| Vasopressor none | - | - | - |  |
| Vasopressor low | 1.858 | 0.798 | 4.723 |  |
| Vasopressor medium | 1.229 | 0.435 | 4.083 |  |
| Vasopressor high | 7.388 | 1.214 | 147.856 |  |
| GFR_Jelliffe | 0.974 | 0.961 | 0.986 | < 0.001 |
| CRRT | 2.597 | 0.653 | 17.474 | 0.191 |
| This retrospective observational cohort study was conducted at Na Homolce Hospital, Prague, Czech Republic (January 2019–December 2022). We included consecutive critically ill ICU patients (N = 377) treated with meropenem, cefepime, or piperacillin–tazobactam for acute infections. Antibiotic plasma trough concentrations (Cmin) were measured after 24 h of full dosing using LC–MS/MS. Antibiotic concentrations were related to breakpoints obtained from the European Committee on Antimicrobial Susceptibility Testing (EUCAST). The MIC values for meropenem, cefepime and piperacillin were set at 8 mg/l, 8 mg/l, and 16 mg/l, respectively. Sample size N= 377. BMI – body mass index, CRRT - continuous renal replacement therapy, GFR_Jelliffe – glomerular filtration rate estimated using the Jelliffe equation, OR = Odds Ratio  **1 Overall model fit: LR χ (-8) = 70.95, p = 3.18e-12; Nagelkerke R² = 0.316; AUC = 0.837 (95% CI 0.776–0.898); Hosmer–Lemeshow**  chi**) = 5.01, p = 0.757.** | | | | |

2e Factors associated with underdosing (Cmin < 1x MIC), GFR estimated using the Cockcroft-Gault equation: All patients. Multivariate logistic regression **^1^**

| Characteristic | OR | Conf.low | Conf.high | p-value |
| --- | --- | --- | --- | --- |
| Age | 1.040 | 1.010 | 1.071 | 0.008 |
| Sex M | 0.792 | 0.342 | 1.711 | 0.562 |
| BMI | 1.026 | 0.968 | 1.092 | 0.396 |
| Vasopressor |  |  |  | 0.396 |
| Vasopressor low | 1.961 | 0.854 | 4.932 |  |
| Vasopressor medium | 1.359 | 0.490 | 4.445 |  |
| Vasopressor high | 8.029 | 1.423 | 153.898 |  |
| GFR_CG | 0.985 | 0.976 | 0.994 | 0.001 |
| CRRT | 2.610 | 0.631 | 17.992 | 0.201 |
| This retrospective observational cohort study was conducted at Na Homolce Hospital, Prague, Czech Republic (January 2019–December 2022). We included consecutive critically ill ICU patients (N = 377) treated with meropenem, cefepime, or piperacillin–tazobactam for acute infections. Antibiotic plasma trough concentrations (Cmin) were measured after 24 h of full dosing using LC–MS/MS. Antibiotic concentrations were related to breakpoints obtained from the European Committee on Antimicrobial Susceptibility Testing (EUCAST). The MIC values for meropenem, cefepime and piperacillin were set at 8 mg/l, 8 mg/l, and 16 mg/l, respectively. Sample size N= 377. BMI – body mass index, CRRT - continuous renal replacement therapy, GFR_CG – glomerular filtration rate estimated using the Cockcroft-Gault equation, OR = Odds Ratio  **^1^Overall model fit: LR χ²(-8) = 63.16, p = 1.12e-10; Nagelkerke R² = 0.284; AUC = 0.815 (95% CI 0.749–0.881); Hosmer–Lemeshow χ²(8) = 13.43, p = 0.0978** | | | | |

**Table S3 (a-e):** The achieved plasma concentrations and factors associated with underdosing for Cmin = 4x-10x MIC

3a Achieved plasma concentrations (Cmin = 4-10x MIC): All patients

| MIC | N | (%) |
| --- | --- | --- |
| <4 | 161 | 42.7% |
| 4-10 | 142 | 37.7% |
| >10 | 74 | 19.6% |
| This retrospective observational cohort study was conducted at Na Homolce Hospital, Prague, Czech Republic (January 2019–December 2022). We included consecutive critically ill ICU patients (N = 377) treated with meropenem, cefepime, or piperacillin–tazobactam for acute infections. Antibiotic plasma trough concentrations (Cmin) were measured after 24 h of full dosing using LC–MS/MS. Antibiotic concentrations were related to breakpoints obtained from the European Committee on Antimicrobial Susceptibility Testing (EUCAST). The MIC values for meropenem, cefepime and piperacillin were set at 8 mg/l, 8 mg/l, and 16 mg/l, respectively. Data are presented as absolute and relative frequencies | | |

3b Factors associated with underdosing (Cmin < 4x MIC): All patients. Exploratory analysis

| **Characteristic** | | **N** | **<4**  N = 161 | **>=4**  N = 216 | | **Test statistic (df/N)** | **p-value^1^** |
| --- | --- | --- | --- | --- | --- | --- | --- |
| Age | | 377 | 66 (56, 72) | 72 (67, 77) | | U=11353; N=161/216 | <0.001^1^ |
| Sex | | 377 |  |  | | χ² =0.58; df=1 | 0.489^2^ |
| F | |  | 49.0 (30.4%) | 58.0 (26.9%) | |  |  |
| M | |  | 112.0 (69.6%) | 158.0 (73.1%) | |  |  |
| Weight | | 377 | 85 (75, 100) | 88 (75, 96) | | U=17786; N=161/216 | 0.704^1^ |
| 1Height | | 377 | 178 (165, 182) | 171 (168, 178) | | U=20143.5; N=161/216 | 0.008^1^ |
| BMI | | 377 | 27.8 (24.8, 32.4) | 28.7 (25.4, 32.4) | | N=161/216 U=16707.5 | 0.516^1^ |
| Vasopressor | | 377 |  |  | | χ² =17.9; df=3 | <0.001^2^ |
| none | | 92.0 (57.1%) | |  | 80.0 (37.0%) | | |
| low | | 36.0 (22.4%) | |  | 62.0 (28.7%) | | |
| medium | | 24.0 (14.9%) | |  | 42.0 (19.4%) | | |
| high | | 9.0 (5.6%) | |  | 32.0 (14.8%) | | |
| GFR_Jelliffe | | 377 | 53 (32, 81) | 30 (18, 33) | | U=28413.5; N=161/216 | <0.001^1^ |
| GFR_Jelliffe | | 377 |  |  | |  | <0.001^3^ |
| >120 | |  | 13.0 (8.1%) | 2.0 (0.9%) | |  |  |
| 48–120 | |  | 79.0 (49.1%) | 18.0 (8.3%) | |  |  |
| 12–48 | |  | 47.0 (29.2%) | 119.0 (55.1%) | |  |  |
| <12 | |  | 3.0 (1.9%) | 24.0 (11.1%) | |  |  |
| GFR_CG | |  | 75 (47, 106) | 30 (30, 47) | | U=27799.5; N=161/216 | <0.001^1^ |
| GFR_CG | |  |  |  | |  | <0.001^1^ |
| >120 | |  | 23.0 (14.3%) | 2.0 (0.9%) | |  |  |
| 48–120 | |  | 94.0 (58.4%) | 49.0 (22.7%) | |  |  |
| 12–48 | |  | 25.0 (15.5%) | 106.0 (49.1%) | |  |  |
| <12 | |  | 0.0 (0.0%) | 6.0 (2.8%) | |  |  |
| CRRT | |  | 19.0 (11.8%) | 53.0 (24.5%) | |  |  |
| CRRT | | 377 |  |  | | χ² =9.68; df=1 | 0.002^2^ |
| No | | 142.0 (88.2%) | |  | 163.0 (75.5%) | | |
| Yes | | 19.0 (11.8%) | |  | 53.0 (24.5%) | | |
| AKI | | 377 | 54.0 (33.5%) | 173.0 (80.1%) | | χ² =83.44; df=1 | <0.001^2^ |
|  | This retrospective observational cohort study was conducted at Na Homolce Hospital, Prague, Czech Republic (January 2019–December 2022). We included consecutive critically ill ICU patients (N = 377) treated with meropenem, cefepime, or piperacillin–tazobactam for acute infections. Antibiotic plasma trough concentrations (Cmin) were measured after 24 h of full dosing using LC–MS/MS. Antibiotic concentrations were related to breakpoints obtained from the European Committee on Antimicrobial Susceptibility Testing (EUCAST). The MIC values for meropenem, cefepime and piperacillin were set at 8 mg/l, 8 mg/l, and 16 mg/l, respectively. Categorical variables presented as N (%). Continuous variables presented as median (Q1, Q3). BMI - Body mass index; GFR Jelliffe – glomerular filtration rate estimated using the Jelliffe equation, GFR_CG – glomerular filtration rate estimated using Cockcroft-Gault equation, CRRT – continuous renal replacement therapy, AKI - acute kidney injury  1 Mann–Whitney U test; ^2^ Pearson’s Chi-squared test, ^3^ Fisher´s exact test | | | | | | |

3c Factors associated with underdosing (Cmin < 4x MIC): All patients. Univariate logistic regression analysis

| **Characteristic** | **OR** | **95% CI** | **p-value** |
| --- | --- | --- | --- |
| Age | 1.06 | 1.03, 1.08 | <0.001 |
| Sex |  |  |  |
| F | — | — |  |
| M | 0.87 | 0.43, 1.67 | 0.7 |
| Weight | 1.00 | 0.98, 1.01 | 0.6 |
| Height | 0.96 | 0.93, 0.99 | 0.008 |
| BMI | 1.05 | 0.99, 1.11 | 0.10 |
| Vasopressor |  |  |  |
| none low | — 2.17 | — 1.05, 4.83 | 0.044 |
| medium | 3.01 | 1.22, 9.09 | 0.029 |
| high | 9.86 | 2.02, 178 | 0.026 |
| GFR_Jelliffe | 0.97 | 0.96, 0.98 | <0.001 |
| GFR_CG | 0.98 | 0.97, 0.98 | <0.001 |
| CRRT |  |  |  |
| No | — | — |  |
| Yes | 6.54 | 1.96, 40.6 | 0.011 |
| AKI |  |  |  |
| No | — | — |  |
| Yes | 10.6 | 5.08, 25.2 | <0.001 |
| This retrospective observational cohort study was conducted at Na Homolce Hospital, Prague, Czech Republic (January 2019–December 2022). We included consecutive critically ill ICU patients (N = 377) treated with meropenem, cefepime, or piperacillin–tazobactam for acute infections. Antibiotic plasma trough concentrations (Cmin) were measured after 24 h of full dosing using LC–MS/MS. Antibiotic concentrations were related to breakpoints obtained from the European Committee on Antimicrobial Susceptibility Testing (EUCAST). The MIC values for meropenem, cefepime and piperacillin were set at 8 mg/l, 8 mg/l, and 16 mg/l, respectively.Sample size N= 377. Univariate logistic regression. Data are presented as OR (odds ratio), 95 % CI (confidence interval) and p-value . BMI - Body mass index; GFR Jelliffe – glomerular filtration rate estimated using the Jelliffe equation, GFR_CG – glomerular filtration rate estimated using Cockcroft-Gault equation, CRRT – continuous renal replacement therapy, AKI - acute kidney injury, CI = Confidence Interval, OR = Odds Ratio | | | |

3d Factors associated with underdosing (Cmin < 4x MIC), GFR estimated using the Jelliffe equation: All patients. Multivariate analysis^1^

| Characteristic | OR | Conf.low | Conf.high | p-value |
| --- | --- | --- | --- | --- |
| Age | 1.017 | 0.992 | 1.042 | 0.187 |
| Sex M | 1.673 | 0.959 | 2.926 | 0.070 |
| BMI | 1.055 | 1.007 | 1.108 | 0.123 |
| Vasopressor |  |  |  | 0.050 |
| Vasopressor low | 1.940 | 1.024 | 3.738 |  |
| Vasopressor medium | 1.023 | 0.500 | 2.108 |  |
| Vasopressor high | 2.805 | 1.038 | 8.598 |  |
| GFR_Jelliffe | 0.942 | 0.926 | 0.956 | <0.001 |
| CRRT | 1.038 | 0.524 | 2.096 | 0.917 |
| This retrospective observational cohort study was conducted at Na Homolce Hospital, Prague, Czech Republic (January 2019–December 2022). We included consecutive critically ill ICU patients (N = 377) treated with meropenem, cefepime, or piperacillin–tazobactam for acute infections. Antibiotic plasma trough concentrations (Cmin) were measured after 24 h of full dosing using LC–MS/MS. Antibiotic concentrations were related to breakpoints obtained from the European Committee on Antimicrobial Susceptibility Testing (EUCAST). The MIC values for meropenem, cefepime and piperacillin were set at 8 mg/l, 8 mg/l, and 16 mg/l, respectively.Sample size N= 377. BMI – body mass index, CRRT - continuous renal replacement therapy, GFR Jelliffe – glomerular filtration rate estimated using the Jelliffe equation, OR = Odds Ratio  **^1^ Overall model fit: LR**  **χ² (-8) = 146.56, p = <2e-16; Nagelkerke R² = 0.433; AUC = 0.848 (95% CI 0.809–0.886); Hosmer–Lemeshow**  **χ² (8) = 7.5, p = 0.484.** | | | | |

3e Factors associated with underdosing (Cmin < 4x MIC), GFR estimated using the Cockcroft-Gault equation: All patients. Multivariate analysis^1^

| Characteristic | OR | Conf.low | Conf.high | p-value |
| --- | --- | --- | --- | --- |
| Age | 1.028 | 1.004 | 1.052 | 0.021 |
| Sex M | 1.631 | 0.936 | 2.851 | 0.084 |
| BMI | 1.010 | 0.966 | 1.057 | 0.673 |
| Vasopressor |  |  |  | 0.008 |
| Vasopressor low | 2.152 | 1.139 | 4.149 |  |
| Vasopressor medium | 1.081 | 0.530 | 2.226 |  |
| Vasopressor high | 3.825 | 1.443 | 11.253 |  |
| GFR_C-G | 0.960 | 0.949 | 0.970 | 0.000 |
| CRRT | 0.612 | 0.291 | 1.297 | 0.198 |
| This retrospective observational cohort study was conducted at Na Homolce Hospital, Prague, Czech Republic (January 2019–December 2022). We included consecutive critically ill ICU patients (N = 377) treated with meropenem, cefepime, or piperacillin–tazobactam for acute infections. Antibiotic plasma trough concentrations (Cmin) were measured after 24 h of full dosing using LC–MS/MS. Antibiotic concentrations were related to breakpoints obtained from the European Committee on Antimicrobial Susceptibility Testing (EUCAST). The MIC values for meropenem, cefepime and piperacillin were set at 8 mg/l, 8 mg/l, and 16 mg/l, respectively. Sample size N= 377. BMI – body mass index, CRRT - continuous renal replacement therapy, GFR_CG – glomerular filtration rate estimated using Cockcroft-Gault equation, OR = Odds Ratio  **^1^ Overall model fit: LR χ² (-8) = 138.61, p = <2e-16; Nagelkerke R² = 0.413; AUC = 0.834 (95% CI 0.793–0.874); Hosmer–Lemeshow χ² (8) = 13.74, p = 0.0888.** | | | | |

**Table S4 (a-c):** Factors associated with overdosing (Cmin > 10x MIC): All patients.

4a Factors associated with overdosing (Cmin > 10x MIC): All patients. Exploratory analysis

| **Characteristic** | | | **N** | **<=10**  N = 303 | **>10**  N = 74 | | **Test statistic (df/N)** | **p-value^1^** | |
| --- | --- | --- | --- | --- | --- | --- | --- | --- | --- |
| Age | | | 377 | 69 (61, 74) | 72 (68, 78) | | U=8263.5; N=303/74 | <0.001^1^ | |
| Sex | | | 377 |  |  | | χ² =1.33; df=1 | 0.314^2^ | |
| F | | |  | 90.0 (29.7%) | 17.0 (23.0%) | |  |  | |
| M | | |  | 213.0 (70.3%) | 57.0 (77.0%) | |  |  | |
| Weight | | | 377 | 85 (75, 96) | 88 (75, 99) | | U=10716; N=303/74 | 0.556^1^ | |
| Height | | | 377 | 174 (166, 180) | 172 (168, 178) | | U=12276; N=303/74 | 0.204^1^ | |
| BMI | | | 377 | 28.3 (24.9, 32.4) | 29.7 (25.6, 32.8) | | U=10326; N=303/74 | 0.293^1^ | |
| Vasopressor | | | 377 |  |  | | χ² =2.16; df=3 | 0.525^2^ | |
| none | | | 142.0 (46.9%) | |  | 30.0 (40.5%) | | | |
| low | | | 77.0 (25.4%) | |  | 21.0 (28.4%) | | | |
| medium | | | 54.0 (17.8%) | |  | 12.0 (16.2%) | | | |
| high | | | 30.0 (9.9%) | |  | 11.0 (14.9%) | | | |
| GFR_Jelliffe | | | 377 | 36 (30, 58.4) | 20 (12.4, 30) | | U=17997; N=303/74 | <0.001^1^ | |
| GFR_Jelliffe | | | 377 |  |  | |  | <0.001^3^ | |
| >120 | | |  | 14.0 (4.6%) | 1.0 (1.4%) | |  |  | |
| 48–120 | | |  | 96.0 (31.7%) | 1.0 (1.4%) | |  |  | |
| 12–48 | | |  | 125.0 (41.3%) | 41.0 (55.4%) | |  |  | |
| <12 | | |  | 10.0 (3.3%) | 17.0 (23.0%) | |  |  | |
| GFR_CG | | |  | 51 (30, 84) | 30 (20, 37) | | U=17608.5; N=303/74 | <0.001^1^ | |
| GFR_CG | | |  |  |  | |  | <0.001^3^ | |
| >120 | | |  | 24.0 (7.9%) | 1.0 (1.4%) | |  |  | |
| 48–120 | | |  | 136.0 (44.9%) | 7.0 (9.5%) | |  |  | |
| 12–48 | | |  | 84.0 (27.7%) | 47.0 (63.5%) | |  |  | |
| <12 | | |  | 1.0 (0.3%) | 5.0 (6.8%) | |  |  | |
| CRRT | | |  | 58.0 (19.1%) | 14.0 (18.9%) | | χ² =0; df=1 |  | |
| CRRT | | | 377 |  |  | |  | >0.999^2^ | |
| No | | | 245.0 (80.9%) | |  | 60.0 (81.1%) | | | |
| Yes | | | 58.0 (19.1%) | |  | 14.0 (18.9%) | | | |
| AKI | | | 377 | 158.0 (52.1%) | 69.0 (93.2%) | | χ² =41.93; df=1 | <0.001^2^ | |
|  | This retrospective observational cohort study was conducted at Na Homolce Hospital, Prague, Czech Republic (January 2019–December 2022). We included consecutive critically ill ICU patients (N = 377) treated with meropenem, cefepime, or piperacillin–tazobactam for acute infections. Antibiotic plasma trough concentrations (Cmin) were measured after 24 h of full dosing using LC–MS/MS. Antibiotic concentrations were related to breakpoints obtained from the European Committee on Antimicrobial Susceptibility Testing (EUCAST). The MIC values for meropenem, cefepime and piperacillin were set at 8 mg/l, 8 mg/l, and 16 mg/l, respectively. Categorical variables presented as N (%). Categorical variables presented as N (%). Continuous variables presented as median (Q1, Q3). BMI - Body mass index; GFR Jelliffe – glomerular filtration rate estimated using the Jelliffe equation, GFR_CG – glomerular filtration rate estimated using Cockcroft-Gault equation, CRRT – continuous renal replacement therapy, AKI - acute kidney injury  1 Mann–Whitney U test; Pearson’s Chi-squared test; Fisher’s exact test | | | | | | |  |  |

4b Factors associated with overdosing (Cmin > 10x MIC): All patients. Univariate logistic regression analysis

| **Characteristic** | **OR** | **95% CI** | **p-value** |
| --- | --- | --- | --- |
| Age | 1.05 | 1.02, 1.08 | <0.001 |
| Sex |  |  |  |
| F | — | — |  |
| M | 1.42 | 0.80, 2.63 | 0.251 |
| Weight | 1.00 | 0.99, 1.02 | 0.786 |
| Height | 0.98 | 0.96, 1.01 | 0.233 |
| BMI | 1.02 | 0.97, 1.06 | 0.414 |
| Vasopressor |  |  |  |
| none low | — 1.29 | — 0.69, 2.40 | 0.422 |
| medium | 1.05 | 0.49, 2.16 | 0.893 |
| high | 1.74 | 0.76, 3.78 | 0.174 |
| GFR_Jelliffe | 0.93 | 0.91, 0.95 | <0.001 |
| GFR_Jelliffe |  |  |  |
| >120  48–120 | — 0.15 | — 0.01, 3.83 | 0.182 |
| 12–48 | 4.59 | 0.88, 84.5 | 0.147 |
| <12 | 23.8 | 3.90, 465 | 0.004 |
| GFR_CG | 0.95 | 0.93, 0.97 | <0.001 |
| GFR_CG |  |  |  |
| >120  48–120 | — 1.24 | — 0.21, 23.6 | 0.8 |
| 12–48 | 13.4 | 2.70, 244 | 0.012 |
| <12 | 120 | 9.72, 4,780 | 0.001 |
| CRRT | 3.38 | 0.60, 63.8 | 0.258 |
| CRRT |  |  |  |
| No | — | — |  |
| Yes | 0.99 | 0.50, 1.84 | 0.965 |
| AKI |  |  |  |
| No | — | — |  |
| Yes | 12.7 | 5.46, 36.9 | <0.001 |
| This retrospective observational cohort study was conducted at Na Homolce Hospital, Prague, Czech Republic (January 2019–December 2022). We included consecutive critically ill ICU patients (N = 377) treated with meropenem, cefepime, or piperacillin–tazobactam for acute infections. Antibiotic plasma trough concentrations (Cmin) were measured after 24 h of full dosing using LC–MS/MS. Antibiotic concentrations were related to breakpoints obtained from the European Committee on Antimicrobial Susceptibility Testing (EUCAST). The MIC values for meropenem, cefepime and piperacillin were set at 8 mg/l, 8 mg/l, and 16 mg/l, respectively. Sample size N= 377. Univariate logistic regression. Data are presented as OR (odds ratio), 95 % CI (confidence interval) and p-value . BMI - Body mass index; GFR Jelliffe – glomerular filtration rate estimated using the Jelliffe equation, GFR_CG – glomerular filtration rate estimated using Cockcroft-Gault equation, CRRT – continuous renal replacement therapy, AKI - acute kidney injury, CI = Confidence Interval, OR = Odds Ratio | | | |

4c Factors associated with overdosing (Cmin > 10x MIC), GFR estimated using the Jelliffe equation: All patients. Multivariate logistic regression analysis^1^

| Characteristic | OR | Conf.low | Conf.high | p-value |
| --- | --- | --- | --- | --- |
| Age | 1.007 | 0.978 | 1.039 | 0.646 |
| Sex M | 2.086 | 1.084 | 4.205 | 0.027 |
| BMI | 1.073 | 1.019 | 1.131 | 0.007 |
| Vasopressor |  |  |  | 0.516 |
| Vasopressor no |  |  |  |  |
| Vasopressor low | 1.302 | 0.624 | 2.705 |  |
| Vasopressor medium | 0.673 | 0.282 | 1.541 |  |
| Vasopressor high | 0.965 | 0.374 | 2.402 |  |
| GFR_Jelliffe | 0.924 | 0.898 | 0.947 | <0.001 |
| CRRT | 0.906 | 0.417 | 1.908 | 0.797 |
| This retrospective observational cohort study was conducted at Na Homolce Hospital, Prague, Czech Republic (January 2019–December 2022). We included consecutive critically ill ICU patients (N = 377) treated with meropenem, cefepime, or piperacillin–tazobactam for acute infections. Antibiotic plasma trough concentrations (Cmin) were measured after 24 h of full dosing using LC–MS/MS. Antibiotic concentrations were related to breakpoints obtained from the European Committee on Antimicrobial Susceptibility Testing (EUCAST). The MIC values for meropenem, cefepime and piperacillin were set at 8 mg/l, 8 mg/l, and 16 mg/l, respectively. Sample size N= 377. BMI – body mass index, CRRT - continuous renal replacement therapy, GFR Jelliffe – glomerular filtration rate estimated using the Jelliffe equation, OR = Odds Ratio  **^1^ Overall model fit: LR χ² (-8) = 79.12, p = 7.35e-14; Nagelkerke R² = 0.301; AUC = 0.832 (95% CI 0.78–0.884); Hosmer–Lemeshow χ² (8) = 25.41, p = 0.0013** | | | | |

4d Factors associated with overdosing (Cmin > 10x MIC), GFR estimated using the Cockcroft-Gault equation: All patients. Multivariate logistic regression analysis**^1^**

| Characteristic | OR | Conf.low | Conf.high | p-value |
| --- | --- | --- | --- | --- |
| Age | 1.015 | 0.987 | 1.045 | 0.305 |
| Sex M | 2.185 | 1.128 | 4.437 | 0.020 |
| BMI | 1.039 | 0.990 | 1.093 | 0.117 |
| Vasopressor |  |  |  | 0.403 |
| Vasopressor low | 1.418 | 0.677 | 2.960 |  |
| Vasopressor medium | 0.686 | 0.288 | 1.565 |  |
| Vasopressor high | 1.174 | 0.455 | 2.938 |  |
| GFR_C-G | 0.944 | 0.924 | 0.961 | 0.000 |
| CRRT | 0.416 | 0.190 | 0.873 | 0.023 |
| This retrospective observational cohort study was conducted at Na Homolce Hospital, Prague, Czech Republic (January 2019–December 2022). We included consecutive critically ill ICU patients (N = 377) treated with meropenem, cefepime, or piperacillin–tazobactam for acute infections. Antibiotic plasma trough concentrations (Cmin) were measured after 24 h of full dosing using LC–MS/MS. Antibiotic concentrations were related to breakpoints obtained from the European Committee on Antimicrobial Susceptibility Testing (EUCAST). The MIC values for meropenem, cefepime and piperacillin were set at 8 mg/l, 8 mg/l, and 16 mg/l, respectively.Sample size N= 377. BMI – body mass index, CRRT - continuous renal replacement therapy, GFR_CG – glomerular filtration rate estimated using Cockcroft-Gault equation , OR = Odds Ratio  **^1^ Overall model fit: LR χ²(-8) = 79.23, p = 7e-14; Nagelkerke R² = 0.302; AUC = 0.826 (95% CI 0.772–0.88); Hosmer–Lemeshow χ² (8) = 20.74, p = 0.00788.** | | | | |

**2 Subanalysis of patients treated with conventional doses (CD patients)**

**Table S5 (a-b**): Baseline characteristics

5a Baseline characteristics: Categorical variables (Subanalysis of patients treated with conventional maximum doses, i.e. meropenem 2 g every 8 hours, cefepime 2 g every 8 hours, or piperacillin–tazobactam 4.5 g every 6 hours)

| **Characteristic** |  | **N = 229** |
| --- | --- | --- |
| Antibiotic |  | |
| Cefepime |  | 57.0 (24.9%) |
| Meropenem |  | 91.0 (39.7%) |
| Piperacillin/ tazobactam |  | 81.0 (35.4%) |
| Sex |  |  |
| F |  | 53.0 (23.1%) |
| M |  | 176.0 (76.9%) |
| Vasopressor  none |  | 108.0 (47.2%) |
| low |  | 53.0 (23.1%) |
| medium |  | 41.0 (17.9%) |
| high |  | 27.0 (11.8%) |
| AKI |  |  |
| No |  | 97.0 (42.4%) |
| Yes |  | 132.0 (57.6%) |
| CRRT |  |  |
| No |  | 180.0 (78.6%) |
| Yes |  | 49.0 (21.4%) |
| GFR_Jelliffe |  |  |
| >120 |  | 2.0 (2.5%) |
| 48–120 |  | 15.0 (18.5%) |
| 12–48 |  | 40.0 (49.4%) |
| <12 |  | 4.0 (4.9%) |
| GFR_CG |  |  |
| >120 |  | 2.0 (2.5%) |
| 48–120 |  | 29.0 (35.8%) |
| 12–48 |  | 30.0 (37.0%) |
| <12 |  | 0.0 (0.0%) |
| CRRT |  | 49.0 (21.4%) |
| ECMO  No |  | 224.0 (97.8%) |
| Yes |  | 5.0 (2.2%) |
| 30 day mortality |  |  |
| No |  | 182.0 (79.5%) |
| Yes |  | 47.0 (20.5%) |
| Indication  CNS |  | 19.0 (8.3%) |
| DMT |  | 22.0 (9.6%) |
| GIT |  | 17.0 (7.4%) |
| HCD |  | 6.0 (2.6%) |
| KVS |  | 35.0 (15.3%) |
| OTH |  | 7.0 (3.1%) |
| PNE |  | 60.0 (26.2%) |
| SEPSIS un |  | 36.0 (15.7%) |
| TRB |  | 21.0 (9.2%) |
| URO |  | 6.0 (2.6%) |
| Sepsis |  |  |
| No |  | 168.0 (73.4%) |
| Yes |  | 61.0 (26.6%) |
| This retrospective observational cohort study was conducted at Na Homolce Hospital, Prague, Czech Republic (January 2019–December 2022). We included consecutive critically ill ICU patients (N = 377) treated with meropenem, cefepime, or piperacillin–tazobactam for acute infections. Patients treated with meropenem 2 g every 8 hours, cefepime 2 g every 8 hours, or piperacillin–tazobactam 4.5 g every 6 hours were included in subanalysis (“CD patients” – patients treated with conventional maximum doses). Sample size N = 229. Antibiotic plasma trough concentrations (Cmin) were measured after 24 h of full dosing using LC–MS/MS. Antibiotic concentrations were related to breakpoints obtained from the European Committee on Antimicrobial Susceptibility Testing (EUCAST). The MIC values for meropenem, cefepime and piperacillin were set at 8 mg/l, 8 mg/l, and 16 mg/l, respectively. Data are presented as absolute and relative frequencies. BMI – Body mass index; GFR Jelliffe – glomerular filtration rate estimated using the Jelliffe equation, GFR_CG – glomerular filtration rate estimated using Cockcroft-Gault equation, AKI –acute kidney injury, ECMO - Extracorporeal membrane oxygenation, CRRT – continuous renal replacement therapy, CNS- central nervous infection, SIN-sinusitis, DMT-soft tissue infections, GIT-abdominal infections, CVS-cardiovascular infections, PNE-pneumonias, TRB-tracheobronchitis, URO-uroinfections, SEPSIS un-sepsis of unknown origin, OTH-other | | |

5b Baseline characteristics: Continuous variables (Subanalysis of patients treated with conventional maximum doses, i.e. meropenem 2 g every 8 hours, cefepime 2 g every 8 hours, or piperacillin–tazobactam 4.5 g every 6 hours)

| variable | mean | sd | p25 | p50 | p75 | min | max |
| --- | --- | --- | --- | --- | --- | --- | --- |
| Age | 66.0 | 12.1 | 61.0 | 68.0 | 74.0 | 22.0 | 89.0 |
| Weight | 89.4 | 16.3 | 80.0 | 89.0 | 99.0 | 49.0 | 172.0 |
| Height | 174.1 | 9.4 | 168.0 | 175.0 | 180.0 | 150.0 | 205.0 |
| BMI | 29.6 | 5.4 | 26.1 | 28.9 | 32.8 | 15.4 | 60.2 |
| GFR_Jelliffe | 41.3 | 43.7 | 25.2 | 30.0 | 46.4 | 7.6 | 372.4 |
| lnGFR_Jelliffe | 3.5 | 0.6 | 3.2 | 3.4 | 3.8 | 2.0 | 5.9 |
| GFR_CG | 55.7 | 48.5 | 30.0 | 41.4 | 60.6 | 12.2 | 404.2 |
| lnGFR_CG | 3.8 | 0.6 | 3.4 | 3.7 | 4.1 | 2.5 | 6.0 |
| This retrospective observational cohort study was conducted at Na Homolce Hospital, Prague, Czech Republic (January 2019–December 2022). We included consecutive critically ill ICU patients (N = 377) treated with meropenem, cefepime, or piperacillin–tazobactam for acute infections. Patients treated with meropenem 2 g every 8 hours, cefepime 2 g every 8 hours, or piperacillin–tazobactam 4.5 g every 6 hours were included in subanalysis (“CD patients” – patients treated with conventional maximum doses). Sample size N = 229. Antibiotic plasma trough concentrations (Cmin) were measured after 24 h of full dosing using LC–MS/MS. Antibiotic concentrations were related to breakpoints obtained from the European Committee on Antimicrobial Susceptibility Testing (EUCAST). The MIC values for meropenem, cefepime and piperacillin were set at 8 mg/l, 8 mg/l, and 16 mg/l, respectively. Data are presented as mean, standard deviation (SD), 25th percentile (P25), median (P50), 75th percentile (P75), minimum, and maximum values. Sample size N= 229. BMI - Body mass index; GFR Jelliffe – glomerular filtration rate estimated using the Jelliffe equation, GFR_CG – glomerular filtration rate estimated using Cockcroft-Gault equation, sd- standard deviation, p – percentile | | | | | | | |

**Table S6(a-e):** The achieved plasma concentrations and factors associated with underdosing for Cmin = 1-10x MIC; subanalysis of patients treated with conventional maximum doses, i.e. meropenem 2 g every 8 hours, cefepime 2 g every 8 hours, or piperacillin–tazobactam 4.5 g every 6 hours

6a Achieved plasma concentrations in patients treated with conventional maximum doses, i.e. i.e. meropenem 2 g every 8 hours, cefepime 2 g every 8 hours, or piperacillin–tazobactam 4.5 g every 6 hours (Cmin = 1-10x MIC)

| MIC | N | (%) |
| --- | --- | --- |
| 1-10 | 156 | 68.1% |
| <1 | 40 | 17.5% |
| >10 | 33 | 14.4% |
| This retrospective observational cohort study was conducted at Na Homolce Hospital, Prague, Czech Republic (January 2019–December 2022). We included consecutive critically ill ICU patients (N = 377) treated with meropenem, cefepime, or piperacillin–tazobactam for acute infections. Patients treated with meropenem 2 g every 8 hours, cefepime 2 g every 8 hours, or piperacillin–tazobactam 4.5 g every 6 hours were included in subanalysis (“CD patients” – patients treated with conventional maximum doses). Sample size N = 229. Antibiotic plasma trough concentrations (Cmin) were measured after 24 h of full dosing using LC–MS/MS. Antibiotic concentrations were related to breakpoints obtained from the European Committee on Antimicrobial Susceptibility Testing (EUCAST). The MIC values for meropenem, cefepime and piperacillin were set at 8 mg/l, 8 mg/l, and 16 mg/l, respectively. Data are presented as absolute and relative frequencies | | |

6b Factors associated with underdosing in patients treated with conventional maximum doses (Cmin < 1x MIC): subanalysis of patients treated with conventional maximum doses, i.e. meropenem 2 g every 8 hours, cefepime 2 g every 8 hours, or piperacillin–tazobactam 4.5 g every 6 hour; Exploratory analysis

| **Characteristic** | | **N** | **<1**  N = 40 | **>=1**  N = 189 | | | Test statistic (df/N) | **p-value^1^** |
| --- | --- | --- | --- | --- | --- | --- | --- | --- |
| Age | | 229 | 59 (49, 68) | 70 (63, 74) | | | U=2050; N=40/189 | <0.001^1^ |
| Sex | | 229 |  |  | | | χ²=0.09; df=1 | 0.837^2^ |
| F | |  | 10.0 (25.0%) | 43.0 (22.8%) | | |  |  |
| M | |  | 30.0 (75.0%) | 146.0 (77.2%) | | |  |  |
| Weight | | 229 | 85 (78, 94) | 89 (80, 99) | | | U=3273.5; N=40/189 | 0.183^1^ |
| Height | | 229 | 180 (167, 182) | 174 (168, 180) | | | U=4289.5; N=40/189 | 0.180^1^ |
| BMI | | 229 | 27.8 (23.4, 33.2) | 29.3 (26.8, 32.4) | | | U=2984.5; N=40/189 | 0.037^1^ |
| Vasopressor | | 229 |  |  | | | χ² =0.09; df=1 | 0.0173^2^ |
| none | | 26.0 (65.0%) | |  | | 82.0 (43.4%) | | |
| low | | 10.0 (25.0%) | |  | | 43.0 (22.8%) | | |
| medium | | 3.0 (7.5%) | |  | | 38.0 (20.1%) | | |
| high | | 1.0 (2.5%) | |  | | 26.0 (13.8%) | | |
| GFR_Jelliffe | | 229 | 65 (48, 101) | 30 (26, 47) | | | U=6274; N=40/189 | <0.001^1^ |
| GFR_Jelliffe | | 229 |  |  | | |  | <0.001^3^ |
| >120 | |  | 8.0 (20.0%) | 4.0 (2.1%) | | |  |  |
| 48–120 | |  | 22.0 (55.0%) | 42.0 (22.2%) | | |  |  |
| 12–48 | |  | 9.0 (22.5%) | 88.0 (46.6%) | | |  |  |
| <12 | |  | 0.0 (0.0%) | 7.0 (3.7%) | | |  |  |
| GFR_CG | |  | 83.5 (60, 126) | 41.1 (30, 65) | | | U=6045; N=40/189 | <0.001^1^ |
| GFR_CG | |  |  |  | | |  | <0.001^3^ |
| >120 | |  | 11.0 (27.5%) | 8.0 (4.2%) | | |  |  |
| 48–120 | |  | 24.0 (60.0%) | 68.0 (36.0%) | | |  |  |
| 12–48 | |  | 4.0 (10.0%) | 64.0 (33.9%) | | |  |  |
| <12 | |  | 0.0 (0.0%) | 1.0 (0.5%) | | |  |  |
| CRRT | |  | 1.0 (2.5%) | 48.0 (25.4%) | | |  |  |
| CRRT | | 229 |  |  | | | χ²=10.29; df=1 | 0.001^2^ |
| No | | 39.0 (97.5%) | |  | 141.0 (74.6%) | | | |
| Yes | | 1.0 (2.5%) | |  | 48.0 (25.4%) | | | |
| AKI | | 229 | 6.0 (15.0%) | 126.0 (66.7%) | | | χ²=36.09; df=1 | <0.001^2^ |
|  | This retrospective observational cohort study was conducted at Na Homolce Hospital, Prague, Czech Republic (January 2019–December 2022). We included consecutive critically ill ICU patients (N = 377) treated with meropenem, cefepime, or piperacillin–tazobactam for acute infections. Patients treated with meropenem 2 g every 8 hours, cefepime 2 g every 8 hours, or piperacillin–tazobactam 4.5 g every 6 hours were included in subanalysis (“CD patients” – patients treated with conventional maximum doses). Sample size N = 229. Antibiotic plasma trough concentrations (Cmin) were measured after 24 h of full dosing using LC–MS/MS. Antibiotic concentrations were related to breakpoints obtained from the European Committee on Antimicrobial Susceptibility Testing (EUCAST). The MIC values for meropenem, cefepime and piperacillin were set at 8 mg/l, 8 mg/l, and 16 mg/l, respectively. Sample size N = 229. Categorical variables presented as N (%). Categorical variables presented as N (%). Continuous variables presented as median (Q1, Q3). BMI - Body mass index; GFR Jelliffe – glomerular filtration rate estimated using the Jelliffe equation, GFR_CG – glomerular filtration rate estimated using Cockcroft-Gault equation, CRRT – continuous renal replacement therapy, AKI - acute kidney injury  ^1^ Mann–Whitney U test; ^2^ Pearson’s Chi-squared test; ^3^ Fisher’s exact test | | | | | | | |

6c Factors associated with underdosing in patients treated with conventional maximum doses (Cmin < 1x MIC): subanalysis of patients treated with conventional maximum doses, i.e. meropenem 2 g every 8 hours, cefepime 2 g every 8 hours, or piperacillin–tazobactam 4.5 g every 6 hour; Univariate logistic regression analysis

| **Characteristic** | **N** | **OR** | **95% CI** | **p-value** |
| --- | --- | --- | --- | --- |
| Age | 229 | 1.07 | 1.04, 1.10 | <0.001 |
| Sex | 229 |  |  |  |
| F |  | — | — |  |
| M |  | 1.13 | 0.49, 2.43 | 0.759 |
| Weight | 229 | 1.02 | 1.00, 1.04 | 0.131 |
| Height | 229 | 0.97 | 0.94, 1.01 | 0.151 |
| BMI | 229 | 1.09 | 1.02, 1.18 | 0.019 |
| Vasopressor | 229 |  |  |  |
| none low |  | — 1.36 | — 0.62, 3.21 | 0.457 |
| medium |  | 4.02 | 1.31, 17.6 | 0.030 |
| high |  | 8.24 | 1.62, 151 | 0.043 |
| GFR_Jelliffe | 229 | 0.97 | 0.96, 0.98 | <0.001 |
| GFR_Jelliffe | 229 |  |  |  |
| >120  48–120 |  | — 3.82 | — 1.08, 15.6 | 0.044 |
| 12–48 |  | 19.6 | 5.17, 86.5 | <0.001 |
| <12 |  | 31,302,722 | 0.00, | 0.985 |
| GFR_CG |  | 0.98 | 0.97, 0.99 | <0.001 |
| GFR_CG5 |  |  |  |  |
| >120 |  | — 3.90 | — 1.41, 11.2 | 0.009 |
| 48–120 |  | 22.0 | 6.07, 96.4 | <0.001 |
| 12–48 |  | 2,912,497 | 0.00, | >0.9 |
| <12 |  | 96.0 | 13.3, 2,042 | <0.001 |
| CRRT | 229 |  |  |  |
| No |  | — | — |  |
| Yes |  | 13.3 | 2.76, 239 | 0.012 |
| AKI | 229 |  |  |  |
| No |  | — | — |  |
| Yes |  | 11.3 | 4.83, 31.3 | <0.001 |
| This retrospective observational cohort study was conducted at Na Homolce Hospital, Prague, Czech Republic (January 2019–December 2022). We included consecutive critically ill ICU patients (N = 377) treated with meropenem, cefepime, or piperacillin–tazobactam for acute infections. Patients treated with meropenem 2 g every 8 hours, cefepime 2 g every 8 hours, or piperacillin–tazobactam 4.5 g every 6 hours were included in subanalysis (“CD patients” – patients treated with conventional maximum doses). Sample size N = 229. Antibiotic plasma trough concentrations (Cmin) were measured after 24 h of full dosing using LC–MS/MS. Antibiotic concentrations were related to breakpoints obtained from the European Committee on Antimicrobial Susceptibility Testing (EUCAST). The MIC values for meropenem, cefepime and piperacillin were set at 8 mg/l, 8 mg/l, and 16 mg/l, respectively. Sample size N= 229. Univariate logistic regression. Data are presented as OR (odds ratio), 95 % CI (confidence interval) and p-value. BMI - Body mass index; GFR Jelliffe – glomerular filtration rate estimated using the Jelliffe equation, GFR_CG – glomerular filtration rate estimated using Cockcroft-Gault equation, CRRT – continuous renal replacement therapy, AKI - acute kidney injury, CI = Confidence Interval, OR = Odds Ratio | | | | |

6d Factors associated with underdosing (Cmin < 1x MIC) in patients treated with conventional maximum doses: subanalysis of patients treated with conventional maximum doses, i.e. meropenem 2 g every 8 hours, cefepime 2 g every 8 hours, or piperacillin–tazobactam 4.5 g every 6 hour; Mutivariate logistic regression analysis^1^, GFR estimated using the Jelliffe equation

| Characteristic | OR | Conf.low | Conf.high | p-value |
| --- | --- | --- | --- | --- |
| Age | 1.031 | 0.992 | 1.072 | 0.120 |
| Sex M | 1.099 | 0.384 | 2.912 | 0.854 |
| BMI | 1.056 | 0.974 | 1.151 | 0.190 |
| Vasopressor |  |  |  | 0.491 |
| Vasopressor low | 1.060 | 0.402 | 2.993 |  |
| Vasopressor medium | 1.260 | 0.353 | 5.983 |  |
| Vasopressor high | 4.387 | 0.706 | 86.835 |  |
| GFR_Jelliffe | 0.976 | 0.961 | 0.990 | 0.002 |
| CRRT | 5.753 | 0.994 | 110.012 | 0.051 |
| This retrospective observational cohort study was conducted at Na Homolce Hospital, Prague, Czech Republic (January 2019–December 2022). We included consecutive critically ill ICU patients (N = 377) treated with meropenem, cefepime, or piperacillin–tazobactam for acute infections. Patients treated with meropenem 2 g every 8 hours, cefepime 2 g every 8 hours, or piperacillin–tazobactam 4.5 g every 6 hours were included in subanalysis (“CD patients” – patients treated with conventional maximum doses). Sample size N = 229. Antibiotic plasma trough concentrations (Cmin) were measured after 24 h of full dosing using LC–MS/MS. Antibiotic concentrations were related to breakpoints obtained from the European Committee on Antimicrobial Susceptibility Testing (EUCAST). The MIC values for meropenem, cefepime and piperacillin were set at 8 mg/l, 8 mg/l, and 16 mg/l, respectively. Sample size N= 229. BMI – body mass index, CRRT - continuous renal replacement therapy, GFR Jelliffe – glomerular filtration rate estimated using the Jelliffe equation, OR = Odds Ratio  **^1^ Overall model fit: LR**  **χ² (-8) = 54.51, p = 5.5e-09; Nagelkerke R² = 0.351; AUC = 0.842 (95% CI 0.777– 0.908); Hosmer–Lemeshow**  **χ² (8) = 4.02, p = 0.855.** | | | | |

6e Factors associated with underdosing (Cmin < 1x MIC) in patients treated with conventional maximum doses: subanalysis of patients treated with conventional maximum doses, i.e. meropenem 2 g every 8 hours, cefepime 2 g every 8 hours, or piperacillin–tazobactam 4.5 g every 6 hour; Mutivariate logistic regression analysis^1^, GFR estimated using the the Cockcroft-Gault equation

| Characteristic | OR | Conf.low | Conf.high | p-value |
| --- | --- | --- | --- | --- |
| Age | 1.048 | 1.012 | 1.085 | 0.009 |
| Sex M | 0.941 | 0.338 | 2.401 | 0.902 |
| BMI | 1.023 | 0.946 | 1.108 | 0.576 |
| Vasopressor |  |  |  | 0.281 |
| Vasopressor low | 1.122 | 0.437 | 3.093 |  |
| Vasopressor medium | 1.442 | 0.410 | 6.796 |  |
| Vasopressor high | 5.715 | 0.975 | 110.581 |  |
| GFR_C-G | 0.988 | 0.977 | 0.998 | 0.027 |
| CRRT | 6.318 | 1.033 | 124.196 | 0.097 |
| This retrospective observational cohort study was conducted at Na Homolce Hospital, Prague, Czech Republic (January 2019–December 2022). We included consecutive critically ill ICU patients (N = 377) treated with meropenem, cefepime, or piperacillin–tazobactam for acute infections. Patients treated with meropenem 2 g every 8 hours, cefepime 2 g every 8 hours, or piperacillin–tazobactam 4.5 g every 6 hours were included in subanalysis (“CD patients” – patients treated with conventional maximum doses). Sample size N = 229. Antibiotic plasma trough concentrations (Cmin) were measured after 24 h of full dosing using LC–MS/MS. Antibiotic concentrations were related to breakpoints obtained from the European Committee on Antimicrobial Susceptibility Testing (EUCAST). The MIC values for meropenem, cefepime and piperacillin were set at 8 mg/l, 8 mg/l, and 16 mg/l, respectively. Sample size N= 229. BMI – body mass index, CRRT - continuous renal replacement therapy, GFR Jelliffe – glomerular filtration rate estimated using the Jelliffe equation, GFR_CG – glomerular filtration rate estimated using Cockcroft-Gault equation , OR = Odds Ratio  **^1^ Overall model fit: LR χ²(-8) = 48.4, p = 8.29e-08; Nagelkerke R² = 0.315; AUC = 0.819 (95% CI 0.747– 0.89); Hosmer–Lemeshow χ²(8) = 4.72, p = 0.787.** | | | | |

**Table S7(a-e):** The achieved plasma concentrations and factors associated with underdosing for Cmin = 4-10x MIC; subanalysis of patients treated with conventional maximum doses, i.e. meropenem 2 g every 8 hours, cefepime 2 g every 8 hours, or piperacillin–tazobactam 4.5 g every 6 hours

7a Achieved plasma concentrations in patients treated with conventional maximum doses, i.e. meropenem 2 g every 8 hours, cefepime 2 g every 8 hours, or piperacillin–tazobactam 4.5 g every 6 hours (Cmin = 4-10x MIC)

| MIC | N | (%) |
| --- | --- | --- |
| <4 | 112 | 48.9% |
| 4-10 | 84 | 36.7% |
| >10 | 33 | 14.4% |
| This retrospective observational cohort study was conducted at Na Homolce Hospital, Prague, Czech Republic (January 2019–December 2022). We included consecutive critically ill ICU patients (N = 377) treated with meropenem, cefepime, or piperacillin–tazobactam for acute infections. Patients treated with meropenem 2 g every 8 hours, cefepime 2 g every 8 hours, or piperacillin–tazobactam 4.5 g every 6 hours were included in subanalysis (“CD patients” – patients treated with conventional maximum doses). Sample size N = 229. Antibiotic plasma trough concentrations (Cmin) were measured after 24 h of full dosing using LC–MS/MS. Antibiotic concentrations were related to breakpoints obtained from the European Committee on Antimicrobial Susceptibility Testing (EUCAST). The MIC values for meropenem, cefepime and piperacillin were set at 8 mg/l, 8 mg/l, and 16 mg/l, respectively. Data are presented as absolute and relative frequencies. | | |

7b Factors associated with underdosing (Cmin < 4x MIC) in patients treated with conventional maximum doses: subanalysis of patients treated with conventional maximum doses, i.e. meropenem 2 g every 8 hours, cefepime 2 g every 8 hours, or piperacillin–tazobactam 4.5 g every 6 hour: Exploratory analysis

| **Characteristic** | | **N** | **<4**  N = 112 | **>=4**  N = 117 | | | **Test statistic (df/N)** | | **p-value^1^** |
| --- | --- | --- | --- | --- | --- | --- | --- | --- | --- |
| Age | | 229 | 66 (55, 72) | 71 (65, 76) | | | U=4484.5; N=112/117 | | <0.001^1^ |
| Sex | | 229 |  |  | | | χ² =0.11; df=1 | | 0.756^2^ |
| F | |  | 27.0 (24.1%) | 26.0 (22.2%) | | |  | |  |
| M | |  | 85.0 (75.9%) | 91.0 (77.8%) | | |  | |  |
| Weight | | 229 | 87 (80, 100) | 90 (80, 98) | | | U=6021; N=112/117 | | 0.290^1^ |
| Height | | 229 | 178 (170, 182) | 172 (168, 179) | | | U=7854.5; N=112/117 | | 0.009^1^ |
| BMI | | 229 | 27.8 (25.2, 32.2) | 29.7 (27.5, 33.2) | | | U=5447;  N=112/117 | | 0.028^1^ |
| Vasopressor | | 229 |  |  | | | χ² =20.7; df=3 | | <0.001^2^ |
| none | | 68.0 (60.7%) | |  | 40.0 (34.2%) | | | | |
| low | | 23.0 (20.5%) | |  | 30.0 (25.6%) | | | | |
| medium | | 16.0 (14.3%) | |  | 25.0 (21.4%) | | | | |
| high | | 5.0 (4.5%) | |  | 22.0 (18.8%) | | | | |
| GFR_Jelliffe | | 229 | 53 (36, 78) | 30 (23, 32) | | | | U=10884; N=112/117 | <0.001^1^ |
| GFR_Jelliffe | | 229 |  |  | | | |  | <0.001^3^ |
| >120 | |  | 11.0 (9.8%) | 1.0 (0.9%) | | | |  |  |
| 48–120 | |  | 56.0 (50.0%) | 8.0 (6.8%) | | | |  |  |
| 12–48 | |  | 33.0 (29.5%) | 64.0 (54.7%) | | | |  |  |
| <12 | |  | 1.0 (0.9%) | 6.0 (5.1%) | | | |  |  |
| GFR_CG | |  | 76 (49, 105) | 31 (30, 47) | | | | U=10764; N=112/117 | <0.001^1^ |
| GFR_CG | |  |  |  | | | |  | <0.001^3^ |
| >120 | |  | 18.0 (16.1%) | 1.0 (0.9%) | | | |  |  |
| 48–120 | |  | 67.0 (59.8%) | 25.0 (21.4%) | | | |  |  |
| 12–48 | |  | 16.0 (14.3%) | 52.0 (44.4%) | | | |  |  |
| <12 | |  | 0.0 (0.0%) | 1.0 (0.9%) | | | |  |  |
| CRRT | |  | 11.0 (9.8%) | 38.0 (32.5%) | | | |  |  |
| CRRT | | 229 |  |  | | | | χ² =17.47; df=1 | <0.001^2^ |
| No | | 101.0 (90.2%) | |  | | 79.0 (67.5%) | | | |
| Yes | | 11.0 (9.8%) | |  | | 38.0 (32.5%) | | | |
| AKI | | 229 | 34.0 (30.4%) | 98.0 (83.8%) | | | χ² =66.84; df=1 | | <0.001^2^ |
|  | This retrospective observational cohort study was conducted at Na Homolce Hospital, Prague, Czech Republic (January 2019–December 2022). We included consecutive critically ill ICU patients (N = 377) treated with meropenem, cefepime, or piperacillin–tazobactam for acute infections. Patients treated with meropenem 2 g every 8 hours, cefepime 2 g every 8 hours, or piperacillin–tazobactam 4.5 g every 6 hours were included in subanalysis (“CD patients” – patients treated with conventional maximum doses). Sample size N = 229. Antibiotic plasma trough concentrations (Cmin) were measured after 24 h of full dosing using LC–MS/MS. Antibiotic concentrations were related to breakpoints obtained from the European Committee on Antimicrobial Susceptibility Testing (EUCAST). The MIC values for meropenem, cefepime and piperacillin were set at 8 mg/l, 8 mg/l, and 16 mg/l, respectively. Categorical variables presented as N (%). Categorical variables presented as N (%). Continuous variables presented as median (Q1, Q3). BMI - Body mass index; GFR Jelliffe – glomerular filtration rate estimated using the Jelliffe equation, GFR_CG – glomerular filtration rate estimated using Cockcroft-Gault equation, CRRT – continuous renal replacement therapy, AKI - acute kidney injury  1 Mann–Whitney U test; ^2^Pearson’s Chi-squared test; ^3^Fisher’s exact test | | | | | | | | |

7c Factors associated with underdosing (Cmin < 4x MIC) in patients treated with conventional maximum doses: subanalysis of patients treated with conventional maximum doses, i.e. meropenem 2 g every 8 hours, cefepime 2 g every 8 hours, or piperacillin–tazobactam 4.5 g every 6 hour: Univariate logistic regression analysis

| **Characteristic** | **OR** | **95% CI** | **p-value** |
| --- | --- | --- | --- |
| Age | 1.05 | 1.02, 1.08 | <0.001 |
| Sex |  |  |  |
| F | — | — |  |
| M | 1.11 | 0.60, 2.06 | 0.735 |
| Weight | 1.01 | 0.99, 1.02 | 0.488 |
| Height | 0.97 | 0.94, 1.00 | 0.031 |
| BMI | 1.06 | 1.00, 1.12 | 0.042 |
| Vasopressor |  |  |  |
| none low | — 2.22 | — 0.62, 3.21 | 0.5 |
| medium | 2.66 | 1.28, 5.65 | 0.010 |
| high | 7.48 | 2.81, 23.7 | <0.001 |
| GFR_Jelliffe | 0.93 | 0.91, 0.95 | <0.001 |
| GFR_Jelliffe |  |  |  |
| >120  48–120 | — 1.57 | — 0.25, 30.7 | 0.684 |
| 12–48 | 21.3 | 3.90, 398 | <0.004 |
| <12 | 66.0 | 5.29, 2,648 | 0.005 |
| GFR_CG | 0.98 | 0.97, 0.99 | <0.001 |
| GFR_CG |  |  |  |
| >120  48–120 | — 3.90 | — 1.41, 11.2 | 0.009 |
| 12–48 | 22.0 | 6.07, 96.4 | <0.001 |
| <12 | 2,912,497 | 0.00, | >0.9 |
| CRRT | 38.0 | 6.38,735 | <0.001 |
| CRRT |  |  |  |
| No | — | — |  |
| Yes | 4.42 | 2.19, 9.58 | <0.001 |
| AKI |  |  |  |
| No | — | — |  |
| Yes | 11.8 | 6.39, 22.8 | <0.001 |
| This retrospective observational cohort study was conducted at Na Homolce Hospital, Prague, Czech Republic (January 2019–December 2022). We included consecutive critically ill ICU patients (N = 377) treated with meropenem, cefepime, or piperacillin–tazobactam for acute infections. Patients treated with meropenem 2 g every 8 hours, cefepime 2 g every 8 hours, or piperacillin–tazobactam 4.5 g every 6 hours were included in subanalysis (“CD patients” – patients treated with conventional maximum doses). Sample size N = 229. Antibiotic plasma trough concentrations (Cmin) were measured after 24 h of full dosing using LC–MS/MS. Antibiotic concentrations were related to breakpoints obtained from the European Committee on Antimicrobial Susceptibility Testing (EUCAST). The MIC values for meropenem, cefepime and piperacillin were set at 8 mg/l, 8 mg/l, and 16 mg/l, respectively. Sample size N= 229. Univariate logistic regression. Univariate logistic regression. Data are presented as OR (odds ratio), 95 % CI (confidence interval) and p-value. BMI - Body mass index; GFR Jelliffe – glomerular filtration rate estimated using the Jelliffe equation, GFR_CG – glomerular filtration rate estimated using Cockcroft-Gault equation, CRRT – continuous renal replacement therapy, AKI - acute kidney injury, CI = Confidence Interval, OR = Odds Ratio | | | |

7d Factors associated with underdosing (Cmin < 4x MIC) in patients treated with conventional maximum doses: subanalysis of patients treated with conventional maximum doses, i.e. meropenem 2 g every 8 hours, cefepime 2 g every 8 hours, or piperacillin–tazobactam 4.5 g every 6 hour, GFR estimated using the Jelliffe equation, Multivariate logistic regression analysis^1^

| Characteristic | OR | Conf.low | Conf.high | p-value |
| --- | --- | --- | --- | --- |
| Age | 1.006 | 0.972 | 1.040 | 0.741 |
| Sex M | 1.529 | 0.689 | 3.409 | 0.295 |
| BMI | 1.104 | 1.022 | 1.198 | 0.111 |
| Vasopressor |  |  |  | 0.121 |
| Vasopressor low | 1.992 | 0.833 | 4.889 |  |
| Vasopressor medium | 1.170 | 0.457 | 2.987 |  |
| Vasopressor high | 3.682 | 1.064 | 16.094 |  |
| GFR_Jelliffe | 0.933 | 0.910 | 0.954 | <0.001 |
| CRRT | 1.467 | 0.601 | 3.736 | 0.403 |
| This retrospective observational cohort study was conducted at Na Homolce Hospital, Prague, Czech Republic (January 2019–December 2022). We included consecutive critically ill ICU patients (N = 377) treated with meropenem, cefepime, or piperacillin–tazobactam for acute infections. Patients treated with meropenem 2 g every 8 hours, cefepime 2 g every 8 hours, or piperacillin–tazobactam 4.5 g every 6 hours were included in subanalysis (“CD patients” – patients treated with conventional maximum doses). Sample size N = 229. Antibiotic plasma trough concentrations (Cmin) were measured after 24 h of full dosing using LC–MS/MS. Antibiotic concentrations were related to breakpoints obtained from the European Committee on Antimicrobial Susceptibility Testing (EUCAST). The MIC values for meropenem, cefepime and piperacillin were set at 8 mg/l, 8 mg/l, and 16 mg/l, respectively. Sample size N= 229. BMI – body mass index, CRRT - continuous renal replacement therapy, GFR Jelliffe – glomerular filtration rate estimated using the Jelliffe equation, OR = Odds Ratio  **^1^Overall model fit: LR**  **χ² (-8) = 103.05, p = <2e-16; Nagelkerke R² = 0.483; AUC = 0.859 (95% CI 0.812–0.906); Hosmer–Lemeshow**  **χ² (8) = 11.03, p = 0.2** | | | | |

7e Factors associated with underdosing (Cmin < 4x MIC) in patients treated with conventional maximum doses: subanalysis of patients treated with conventional maximum doses, i.e. meropenem 2 g every 8 hours, cefepime 2 g every 8 hours, or piperacillin–tazobactam 4.5 g every 6 hour, GFR estimated using the Cockcroft-Gault equation, Multivariate logistic regression analysis^1^

| Characteristic | OR | Conf.low | Conf.high | p-value |
| --- | --- | --- | --- | --- |
| Age | 1.024 | 0.992 | 1.057 | 0.144 |
| Sex M | 1.296 | 0.589 | 2.856 | 0.517 |
| BMI | 1.037 | 0.966 | 1.114 | 0.317 |
| Vasopressor |  |  |  | 0.011 |
| Vasopressor low | 2.358 | 0.995 | 5.786 |  |
| Vasopressor medium | 1.265 | 0.497 | 3.242 |  |
| Vasopressor high | 6.303 | 1.824 | 26.639 |  |
| GFR_C-G | 0.955 | 0.939 | 0.969 | 0.000 |
| CRRT | 0.834 | 0.311 | 2.283 | 0.720 |
| This retrospective observational cohort study was conducted at Na Homolce Hospital, Prague, Czech Republic (January 2019–December 2022). We included consecutive critically ill ICU patients (N = 377) treated with meropenem, cefepime, or piperacillin–tazobactam for acute infections. Patients treated with meropenem 2 g every 8 hours, cefepime 2 g every 8 hours, or piperacillin–tazobactam 4.5 g every 6 hours were included in subanalysis (“CD patients” – patients treated with conventional maximum doses). Sample size N = 229. Antibiotic plasma trough concentrations (Cmin) were measured after 24 h of full dosing using LC–MS/MS. Antibiotic concentrations were related to breakpoints obtained from the European Committee on Antimicrobial Susceptibility Testing (EUCAST). The MIC values for meropenem, cefepime and piperacillin were set at 8 mg/l, 8 mg/l, and 16 mg/l, respectively. Sample size N= 229. BMI – body mass index, CRRT - continuous renal replacement therapy, GFR_CG – glomerular filtration rate estimated using Cockcroft-Gault equation, OR = Odds Ratio  **^1^ Overall model fit: LR**  **χ²(-8) = 96.45, p = <2e-16; Nagelkerke R² = 0.458; AUC = 0.843 (95% CI 0.793– 0.893); Hosmer–Lemeshow χ² (8) = 10.02, p = 0.263.** | | | | |

**Table S8 (a-d):** Factors associated with overdosing (Cmin > 10x MIC)

8a: Factors associated with overdosing (Cmin > 10x MIC) in patients treated with conventional maximum doses: subanalysis of patients treated with conventional maximum doses, i.e. meropenem 2 g every 8 hours, cefepime 2 g every 8 hours, or piperacillin–tazobactam 4.5 g every 6 hour: Exploratory analysis

| **Characteristic** | | **N** | **<=10**  N = 196 | **>10**  N = 33 | | **Test statistic (df/N**) | **p-value** |
| --- | --- | --- | --- | --- | --- | --- | --- |
| Age | | 229 | 67 (59, 73) | 73 (68, 79) | | U=1874; N=196/33 | <0.001^1^ |
| Sex | | 229 |  |  | |  | 0.122^2^ |
| F | |  | 49.0 (25.0%) | 4.0 (12.1%) | | χ² =2.63; df=1 |  |
| M | |  | 147.0 (75.0%) | 29.0 (87.9%) | |  |  |
| Weight | | 229 | 88.4 (80, 99) | 89 (84, 103) | | U=2911; N=196/33 | 0.359^1^ |
| Height | | 229 | 175 (169, 181) | 172 (168, 180) | | U=3514.5; N=196/33 | 0.425^1^ |
| BMI | | 229 | 28.7 (26.1, 32.8) | 29.7 (27.6, 32.8) | | U=2862; N=196/33 | 0.291^1^ |
| Vasopressor | | 229 |  |  | |  | 0.125^3^ |
| none | | 96.0 (49.0%) | | 12 (36.4%) |  | | |
| low | | 45.0 (23.0%) | | 8.0 (24.2%) |  | | |
| medium | | 36.0 (18.4%) | | 5.0 (15.2%) |  | | |
| high | | 19.0 (9.7%) | | 8.0 (24.2%) |  | | |
| GFR_Jelliffe | | 229 | 37 (30, 58) | 23 (14, 30) | | U=5462; N=196/33 | <0.001^1^ |
| GFR_Jelliffe | | 229 |  |  | |  | <0.001^3^ |
| >120 | |  | 12.0 (6.1%) | 0.0 (0.0%) | |  |  |
| 48–120 | |  | 63.0 (32.1%) | 1.0 (3.0%) | |  |  |
| 12–48 | |  | 76.0 (38.8%) | 21.0 (63.6%) | |  |  |
| <12 | |  | 3.0 (1.5%) | 4.0 (12.1%) | |  |  |
| GFR_CG | |  | 53 (30, 83) | 30 (23, 37) | |  | <0.001^1^ |
| GFR_CG5 | |  |  |  | |  | <0.001^3^ |
| >120 | |  | 19.0 (9.7%) | 0.0 (0.0%) | |  |  |
| 48–120 | |  | 89.0 (45.4%) | 3.0 (9.1%) | |  |  |
| 12–48 | |  | 46.0 (23.5%) | 22.0 (66.7%) | |  |  |
| <12 | |  | 0.0 (0.0%) | 1.0 (3.0%) | |  |  |
| CRRT | |  | 42.0 (21.4%) | 7.0 (21.2%) | | χ² =0; df=1 |  |
| CRRT | | 229 |  |  | |  | >0.999^2^ |
| No | | 154.0 (78.6%) | | 26.0 (78.8%) |  | | |
| Yes | | 42.0 (21.4%) | | 7.0 (21.2%) |  | | |
| AKI | | 229 | 100.0 (51.0%) | 32.0 (97.0%) | | χ² =24.42; df=1 | <0.001^2^ |
|  | This retrospective observational cohort study was conducted at Na Homolce Hospital, Prague, Czech Republic (January 2019–December 2022). We included consecutive critically ill ICU patients (N = 377) treated with meropenem, cefepime, or piperacillin–tazobactam for acute infections. Patients treated with meropenem 2 g every 8 hours, cefepime 2 g every 8 hours, or piperacillin–tazobactam 4.5 g every 6 hours were included in subanalysis (“CD patients” – patients treated with conventional maximum doses). Antibiotic concentrations were related to breakpoints obtained from the European Committee on Antimicrobial Susceptibility Testing (EUCAST). The MIC values for meropenem, cefepime and piperacillin were set at 8 mg/l, 8 mg/l, and 16 mg/l, respectively. Categorical variables presented as N (%). Categorical variables presented as N (%). Continuous variables presented as median (Q1, Q3). BMI - Body mass index; GFR Jelliffe – glomerular filtration rate estimated using the Jelliffe equation, GFR_CG – glomerular filtration rate estimated using Cockcroft-Gault equation, CRRT – continuous renal replacement therapy, AKI - acute kidney injury  1 Mann–Whitney U test; ^2^ Pearson’s Chi-squared test; ^3^ Fisher’s exact test | | | | | | |

8b: Factors associated with overdosing (Cmin > 10x MIC) in patients treated with conventional maximum doses, i.e. meropenem 2 g every 8 hours, cefepime 2 g every 8 hours, or piperacillin–tazobactam 4.5 g every 6 hour: Univariate logistic regression analysis

| **Characteristic** | **OR** | **95% CI** | **p-value** |
| --- | --- | --- | --- |
| Age | 1.09 | 1.04, 1.15 | <0.001 |
| Sex |  |  |  |
| F | — | — |  |
| M | 2.42 | 0.90, 8.45 | 0.114 |
| Weight | 1.01 | 0.98, 1.03 | 0.423 |
| Height | 0.99 | 0.95, 1.03 | 0.564 |
| BMI | 1.03 | 0.97, 1.10 | 0.222 |
| Vasopressor |  |  |  |
| none low | — 1.42 | — 0.52, 3.68 | 0.473 |
| medium | 1.11 | 0.33, 3.23 | 0.853 |
| high | 3.37 | 1.18, 9.32 | 0.020 |
| GFR_Jelliffe | 0.90 | 0.86, 0.94 | <0.001 |
| GFR_CG | 0.95 | 0.92, 0.97 | <0.001 |
| CRRT |  |  |  |
| No | — | — |  |
| Yes | 0.99 | 0.37, 2.33 | 0.978 |
| AKI |  |  |  |
| No | — | — |  |
| Yes | 30.7 | 6.41, 552 | <0.001 |
| This retrospective observational cohort study was conducted at Na Homolce Hospital, Prague, Czech Republic (January 2019–December 2022). We included consecutive critically ill ICU patients (N = 377) treated with meropenem, cefepime, or piperacillin–tazobactam for acute infections. Patients treated with meropenem 2 g every 8 hours, cefepime 2 g every 8 hours, or piperacillin–tazobactam 4.5 g every 6 hours were included in subanalysis (“CD patients” – patients treated with conventional maximum doses). Antibiotic concentrations were related to breakpoints obtained from the European Committee on Antimicrobial Susceptibility Testing (EUCAST). The MIC values for meropenem, cefepime and piperacillin were set at 8 mg/l, 8 mg/l, and 16 mg/l, respectively. Sample size N= 229. Univariate logistic regression. Univariate logistic regression. Data are presented as OR (odds ratio), 95 % CI (confidence interval) and p-value. BMI - Body mass index; GFR Jelliffe – glomerular filtration rate estimated using the Jelliffe equation, GFR-CG – glomerular filtration rate estimated using Cockcroft-Gault equation, CRRT – continuous renal replacement therapy, AKI - acute kidney injury, CI = Confidence Interval, OR = Odds Ratio | | | |

8c Factors associated with overdosing (Cmin > 10x MIC) in patients treated with conventional maximum doses, i.e. meropenem 2 g every 8 hours, cefepime 2 g every 8 hours, or piperacillin–tazobactam 4.5 g every 6 hour, GFR estimated using the Jelliffe equation: Multivariate logistic regression analysis^1^

| Characteristic | OR | Conf.low | Conf.high | p-value |
| --- | --- | --- | --- | --- |
| Age | 1.053 | 0.999 | 1.116 | 0.067 |
| Sex M | 3.692 | 1.096 | 16.701 | 0.054 |
| BMI | 1.095 | 0.985 | 1.227 | 0.093 |
| Vasopressor |  |  |  | 0.624 |
| Vasopressor low | 1.166 | 0.353 | 3.722 |  |
| Vasopressor medium | 0.777 | 0.195 | 2.810 |  |
| Vasopressor high | 1.975 | 0.520 | 7.388 |  |
| GFR_Jelliffe | 0.907 | 0.860 | 0.947 | <0.001 |
| CRRT | 0.822 | 0.261 | 2.470 | 0.728 |
| This retrospective observational cohort study was conducted at Na Homolce Hospital, Prague, Czech Republic (January 2019–December 2022). We included consecutive critically ill ICU patients (N = 377) treated with meropenem, cefepime, or piperacillin–tazobactam for acute infections. Patients treated with meropenem 2 g every 8 hours, cefepime 2 g every 8 hours, or piperacillin–tazobactam 4.5 g every 6 hours were included in subanalysis (“CD patients” – patients treated with conventional maximum doses). Antibiotic concentrations were related to breakpoints obtained from the European Committee on Antimicrobial Susceptibility Testing (EUCAST). The MIC values for meropenem, cefepime and piperacillin were set at 8 mg/l, 8 mg/l, and 16 mg/l, respectively. Sample size N= 299. BMI – body mass index, CRRT - continuous renal replacement therapy, , GFR Jelliffe – glomerular filtration rate estimated using the Jelliffe equation, , OR = Odds Ratio  **^1^Overall model fit: LR**  **χ² (-8) = 57.78, p = 1.27e-09; Nagelkerke R² = 0.397; AUC = 0.867 (95% CI 0.8–0.934); Hosmer–Lemeshow**  **χ² (8) = 17.48, p = 0.0255.** | | | | |

8d Factors associated with overdosing (Cmin > 10x MIC), in patients treated with conventional maximum doses, i.e. meropenem 2 g every 8 hours, cefepime 2 g every 8 hours, or piperacillin–tazobactam 4.5 g every 6 hour, GFR estimated using the Cockcroft-Gault equation: Multivariate logistic regression analysis**^1^**

| Characteristic | OR | Conf.low | Conf.high | p-value |
| --- | --- | --- | --- | --- |
| Age | 1.065 | 1.015 | 1.126 | 0.016 |
| Sex M | 3.601 | 1.082 | 15.820 | 0.056 |
| BMI | 1.042 | 0.945 | 1.155 | 0.414 |
| Vasopressor |  |  |  | 0.388 |
| Vasopressor low | 1.299 | 0.402 | 4.050 |  |
| Vasopressor medium | 0.741 | 0.189 | 2.609 |  |
| Vasopressor high | 2.472 | 0.651 | 9.288 |  |
| GFR_C-G | 0.941 | 0.908 | 0.968 | 0.000 |
| CRRT | 0.326 | 0.102 | 0.957 | 0.047 |
| This retrospective observational cohort study was conducted at Na Homolce Hospital, Prague, Czech Republic (January 2019–December 2022). We included consecutive critically ill ICU patients (N = 377) treated with meropenem, cefepime, or piperacillin–tazobactam for acute infections. Patients treated with meropenem 2 g every 8 hours, cefepime 2 g every 8 hours, or piperacillin–tazobactam 4.5 g every 6 hours were included in subanalysis (“CD patients” – patients treated with conventional maximum doses). Antibiotic concentrations were related to breakpoints obtained from the European Committee on Antimicrobial Susceptibility Testing (EUCAST). The MIC values for meropenem, cefepime and piperacillin were set at 8 mg/l, 8 mg/l, and 16 mg/l, respectively. Sample size N= 299. BMI – body mass index, CRRT - continuous renal replacement therapy, GFR_CG – glomerular filtration rate estimated using Cockcroft-Gault equation , OR = Odds Ratio  ^1^ **Overall model fit: LR χ² (-8) = 52.29, p = 1.48e-08; Nagelkerke R² = 0.363; AUC = 0.848 (95% CI 0.77–0.926); Hosmer–Lemeshow χ² (8) = 15.73, p = 0.0465.** | | | | |

**3 Results stratification according to dose number at time of level collection**

**Table S9 (a-j)** Dose regimen every 8 hours (i.e. 3 doses/24 hours)

9a Results stratification according to dose number at time of level collection: Achieved plasma concentrations in patients receiving an every 8-hour dosing regimen (i.e. 3 doses/24 hours)

| MIC | N | % |
| --- | --- | --- |
| 1-10 | 128 | 69.9% |
| >10 | 32 | 17.5% |
| <1 | 23 | 12.6% |
| This retrospective observational cohort study was conducted at Na Homolce Hospital, Prague, Czech Republic (January 2019–December 2022). We included consecutive critically ill ICU patients (N = 377) treated with meropenem, cefepime, or piperacillin–tazobactam for acute infections. Antibiotic plasma trough concentrations (Cmin) were measured after 24 h of full dosing using LC–MS/MS. Antibiotic concentrations were related to breakpoints obtained from the European Committee on Antimicrobial Susceptibility Testing (EUCAST). The MIC values for meropenem, cefepime and piperacillin were set at 8 mg/l, 8 mg/l, and 16 mg/l, respectively. Patients who were receiving an every-8-hour dosing regimen were included in subanalysis. Data are presented as absolute and relative frequencies. | | |

| MIC | N | % |
| --- | --- | --- |
| <4 | 85 | 46.4% |
| 4-10 | 66 | 36.1% |
| >10 | 32 | 17.5% |
| This retrospective observational cohort study was conducted at Na Homolce Hospital, Prague, Czech Republic (January 2019–December 2022). We included consecutive critically ill ICU patients (N = 377) treated with meropenem, cefepime, or piperacillin–tazobactam for acute infections. Antibiotic plasma trough concentrations (Cmin) were measured after 24 h of full dosing using LC–MS/MS. Antibiotic concentrations were related to breakpoints obtained from the European Committee on Antimicrobial Susceptibility Testing (EUCAST). The MIC values for meropenem, cefepime and piperacillin were set at 8 mg/l, 8 mg/l, and 16 mg/l, respectively. Patients who were receiving an every-8-hour dosing regimen were included in subanalysis. Data are presented as absolute and relative frequencies | | |

9b Results stratification according to dose number at time of level collection: Factors associated with underdosing in patients receiving an every 8-hour dosing regimen (i.e 3 doses/24 hours) (Cmin < 1x MIC), Exploratory analysis

| **Characteristic** | | **N** | **<1**  N = 23 | **>=1**  N = 160 | | Test statistic (df/N) | **p-value^1^** |
| --- | --- | --- | --- | --- | --- | --- | --- |
| Age | | 183 | 53 (39, 67) | 69 (62, 74) | | U=992; N=23/160 | <0.001^1^ |
| Sex | | 183 |  |  | | χ² =0; df=1 | >0.999^2^ |
| F | |  | 6.0 (26.1%) | 42.0 (26.3%) | |  |  |
| M | |  | 17.0 (73.9%) | 118.0 (73.8%) | |  |  |
| Weight | | 183 | 87.5 (72, 95) | 89 (80, 100) | | U=1629.5; N=23/160 | 0.376^1^ |
| Height | | 183 | 180 (165, 180) | 173 (168, 180) | | U=2104.5; N=23/160 | 0.265^1^ |
| BMI | | 183 | 26.3 (22.9, 33.0) | 29.4 (26.1, 33.3) | | U=1503; N=23/160 | 0.157^1^ |
| Vasopressor | | 183 |  |  | |  | 0.125^3^ |
| None | | 17.0 (73.9%) | |  | 73.0 (45.6%) | | |
| low | | 3.0 (13.0%) | |  | 39.0 (24.4%) | | |
| medium | | 2.0 (8.7%) | |  | 27.0 (16.9%) | | |
| high | | 1.0 (4.3%) | |  | 21.0 (13.1%) | | |
| GFR_Jelliffe | | 183 | 64 (52, 121) | 30 (26, 52) | | U=3165; N=23/160 | <0.001^1^ |
| GFR_Jelliffe | | 183 |  |  | |  | <0.001^3^ |
| >120 | |  | 6.0 (26.1%) | 4.0 (2.5%) | |  |  |
| 48–120 | |  | 13.0 (56.5%) | 42.0 (26.3%) | |  |  |
| 12–48 | |  | 4.0 (17.4%) | 71.0 (44.4%) | |  |  |
| <12 | |  | 0.0 (0.0%) | 8.0 (5.0%) | |  |  |
| CRRT | |  | 0.0 (0.0%) | 35.0 (21.9%) | |  |  |
| CRRT | | 183 |  |  | |  | 0.009^3^ |
| No | | 23.0 (100.0%) | |  | 125.0 (78.1%) | | |
| Yes | | 0.0 (0.0%) | |  | 35.0 (21.9%) | | |
| AKI | | 183 | 2.0 (8.7%) | 99.0 (61.9%) | | χ² =23; df=1 | <0.001^2^ |
|  | This retrospective observational cohort study was conducted at Na Homolce Hospital, Prague, Czech Republic (January 2019–December 2022). We included consecutive critically ill ICU patients (N = 377) treated with meropenem, cefepime, or piperacillin–tazobactam for acute infections. Antibiotic plasma trough concentrations (Cmin) were measured after 24 h of full dosing using LC–MS/MS. Antibiotic concentrations were related to breakpoints obtained from the European Committee on Antimicrobial Susceptibility Testing (EUCAST). The MIC values for meropenem, cefepime and piperacillin were set at 8 mg/l, 8 mg/l, and 16 mg/l, respectively. Patients who were receiving an every-8-hour dosing regimen were included in subanalysis. Sample size N = 183. Categorical variables presented as N (%). Continuous variables presented as median (Q1, Q3). BMI - Body mass index; GFR – glomerular filtration rate, CRRT – continuous renal replacement therapy, AKI - acute kidney injury  1 Mann–Whitney U test; ^2^ Pearson’s Chi-squared test; ^3^ Fisher’s exact test | | | | | | |

####

9c Results stratification according to dose number at time of level collection: Factors associated with underdosing in patients receiving an every 8-hour dosing regimen (i.e 3 doses/24 hours) (Cmin < 1x MIC), Univariate logistic regression analysis

| **Characteristic** | **N** | **OR** | **95% CI** | **p-value** |
| --- | --- | --- | --- | --- |
| Age | 183 | 1.07 | 1.04, 1.11 | <0.001 |
| Sex | 183 |  |  |  |
| F |  | — | — |  |
| M |  | 0.99 | 0.34, 2.57 | 0.987 |
| Weight | 183 | 1.01 | 0.99, 1.04 | 0.252 |
| Height | 183 | 0.97 | 0.92, 1.02 | 0.253 |
| BMI | 183 | 1.07 | 0.99, 1.16 | 0.107 |
| Vasopressor | 183 | | | |
| none | — — | | | |
| low | 3.03 | | 0.94, 13.5 | 0.092 |
| medium | 3.14 | | 0.83, 20.7 | 0.142 |
| high | 4.89 | | 0.92, 90.7 | 0.134 |
| GFR_Jelliffe | 183 | 0.97 | 0.95, 0.98 | <0.001 |
| CRRT 183  No — | | | — | |
| Yes 21,279,378 | | | 0.00, | 0.988 |
| AKI | 183 | | | |
| No | — — | | | |
| Yes | 17.0 | | 4.77, 109 | <0.001 |
| This retrospective observational cohort study was conducted at Na Homolce Hospital, Prague, Czech Republic (January 2019–December 2022). We included consecutive critically ill ICU patients (N = 377) treated with meropenem, cefepime, or piperacillin–tazobactam for acute infections. Antibiotic plasma trough concentrations (Cmin) were measured after 24 h of full dosing using LC–MS/MS. Antibiotic concentrations were related to breakpoints obtained from the European Committee on Antimicrobial Susceptibility Testing (EUCAST). The MIC values for meropenem, cefepime and piperacillin were set at 8 mg/l, 8 mg/l, and 16 mg/l, respectively. Patients who were receiving an every-8-hour dosing regimen were included in subanalysis. Sample size N = 183. Univariate logistic regression. Data are presented as OR (odds ratio), 95 % CI (confidence interval) and p-value. BMI - Body mass index; GFR – glomerular filtration rate, CRRT – continuous renal replacement therapy, AKI - acute kidney injury, OR = odds ratio | | | | |

9d Results stratification according to dose number at time of level collection: Factors associated with underdosing in patients receiving an every 8-hour dosing regimen (i.e 3 doses/24 hours) (Cmin < 1x MIC), Multivariate logistic regression analysis^1^

| Characteristic | OR | Conf.low | Conf.high | p-value |
| --- | --- | --- | --- | --- |
| Age | 1.036 | 0.992 | 1.081 | 0.100 |
| Sex M | 0.891 | 0.238 | 2.876 | 0.853 |
| BMI | 1.034 | 0.944 | 1.138 | 0.472 |
| Vasopressor |  |  |  | 0.223 |
| Vasopressor low | 4.100 | 0.973 | 25.468 |  |
| Vasopressor medium | 1.386 | 0.313 | 9.738 |  |
| Vasopressor high | 3.487 | 0.496 | 73.791 |  |
| GFR_Jelliffe | 0.972 | 0.954 | 0.988 | < 0.001 |
| CRRT | 1.036 | 0.992 | 1.081 | 0.100 |
| This retrospective observational cohort study was conducted at Na Homolce Hospital, Prague, Czech Republic (January 2019–December 2022). We included consecutive critically ill ICU patients (N = 377) treated with meropenem, cefepime, or piperacillin–tazobactam for acute infections. Antibiotic plasma trough concentrations (Cmin) were measured after 24 h of full dosing using LC–MS/MS. Antibiotic concentrations were related to breakpoints obtained from the European Committee on Antimicrobial Susceptibility Testing (EUCAST). The MIC values for meropenem, cefepime and piperacillin were set at 8 mg/l, 8 mg/l, and 16 mg/l, respectively. Patients who were receiving an every-8-hour dosing regimen were included in subanalysis. Sample size N = 183. BMI – body mass index, CRRT - continuous renal replacement therapy, GFR – glomerular filtration rate, OR = Odds Ratio  **^1^Overall model fit: LR**  **χ² (-7) = 37.27, p = 4.18e-06; Nagelkerke R² = 0.347; AUC = 0.85 (95% CI 0.764– 0.936); Hosmer–Lemeshow**  **χ² (8) = 5.87, p = 0.662.** | | | | |

9e Results stratification according to dose number at time of level collection: Factors associated with underdosing (Cmin < 4x MIC) in patients receiving an every 8-hour dosing regimen (i.e 3 doses/24 hours) (Cmin < 1x MIC), Exploratory analysis

| **Characteristic** | | **N** | **<4**  N = 85 | **>=4**  N = 98 | | | Test statistic (df/N) | **p-value^1^** |
| --- | --- | --- | --- | --- | --- | --- | --- | --- |
| Age | | 183 | 66 (53, 72) | 70 (62, 77) | | | U=3104.5; N=85/98 | 0.003^1^ |
| Sex | | 183 |  |  | | | χ² =0.06; df=1 | 0.867^1^ |
| F | |  | 23.0 (27.1%) | 25.0 (25.5%) | | |  |  |
| M | |  | 62.0 (72.9%) | 73.0 (74.5%) | | |  |  |
| Weight | | 183 | 88 (80, 100) | 90 (76, 99) | | | U=4195; N=85/98 | 0.934^1^ |
| Height | | 183 | 178 (168, 182) | 172 (168, 178) | | | U=5045.5; N=85/98 | 0.014^1^ |
| BMI | | 183 | 28.0 (25.4, 33.0) | 29.7 (25.9, 33.3) | | | U=3952; N=85/98 | 0.552^1^ |
| Vasopressor | | 183 |  |  | | | χ² =12.6; df=3 | 0.006^2^ |
| none | | 53.0 (62.4%) | |  | 37.0 (37.8%) | | | |
| low | | 16.0 (18.8%) | |  | 26.0 (26.5%) | | | |
| medium | | 11.0 (12.9%) | |  | 18.0 (18.4%) | | | |
| high | | 5.0 (5.9%) | |  | 17.0 (17.3%) | | | |
| GFR_Jelliffe | | 183 | 58 (38, 80) | 30 (20, 33) | | | U=7026; N=85/98 | <0.001^1^ |
| GFR_Jelliffe | | 183 |  |  | | |  | <0.001^3^ |
| >120 | |  | 9.0 (10.6%) | 1.0 (1.0%) | | |  |  |
| 48–120 | |  | 46.0 (54.1%) | 9.0 (9.2%) | | |  |  |
| 12–48 | |  | 23.0 (27.1%) | 52.0 (53.1%) | | |  |  |
| <12 | |  | 1.0 (1.2%) | 7.0 (7.1%) | | |  |  |
| CRRT | |  | 6.0 (7.1%) | 29.0 (29.6%) | | |  |  |
| CRRT | | 183 |  |  | | | χ² =14.9; df=1 | <0.001^2^ |
| No | | 79.0 (92.9%) | |  | | 69.0 (70.4%) | | |
| Yes | | 6.0 (7.1%) | |  | | 29.0 (29.6%) | | |
| AKI | | 183 | 22.0 (25.9%) | 79.0 (80.6%) | | | χ² =55.1; df=1 | <0.001^2^ |
|  | This retrospective observational cohort study was conducted at Na Homolce Hospital, Prague, Czech Republic (January 2019–December 2022). We included consecutive critically ill ICU patients (N = 377) treated with meropenem, cefepime, or piperacillin–tazobactam for acute infections. Antibiotic plasma trough concentrations (Cmin) were measured after 24 h of full dosing using LC–MS/MS. Antibiotic concentrations were related to breakpoints obtained from the European Committee on Antimicrobial Susceptibility Testing (EUCAST). The MIC values for meropenem, cefepime and piperacillin were set at 8 mg/l, 8 mg/l, and 16 mg/l, respectively. Patients who were receiving an every-8-hour dosing regimen were included in subanalysis. Sample size N = 183. Categorical variables presented as N (%). Continuous variables presented as median (Q1, Q3). BMI - Body mass index; GFR – glomerular filtration rate, CRRT – continuous renal replacement therapy, AKI - acute kidney injury  1 Mann–Whitney U test; ^2^Pearson’s Chi-squared test; ^3^Fisher’s exact test | | | | | | | |

9f Results stratification according to dose number at time of level collection: Factors associated with underdosing (Cmin < 4x MIC) in patients receiving an every 8-hour dosing regimen (i.e 3 doses/24 hours) (Cmin < 1x MIC), Univariate logistic regression analysis

| **Characteristic** | **N** | **OR** | **95% CI** | **p-value** |
| --- | --- | --- | --- | --- |
| Age | 183 | 1.04 | 1.02, 1.07 | 0.001 |
| Sex | 183 |  |  |  |
| F |  | — | — |  |
| M |  | 1.08 | 0.56, 2.10 | 0.812 |
| Weight | 183 | 1.0 | 0.98, 1.01 | 0.500 |
| Height | 183 | 0.96 | 0.93, 0.99 | 0.021 |
| BMI | 183 | 1.01 | 0.96, 1.06 | 0.645 |
| Vasopressor | 183 | | | |
| none | — — | | | |
| low | 2.33 | | 1.11, 5.01 | 0.027 |
| medium | 2.34 | | 1.00, 5.68 | 0.052 |
| high | 4.87 | | 1.75, 15.9 | 0.004 |
| GFR_Jelliffe | 183 | 0.93 | 0.91, 0.95 | <0.001 |
| CRRT 183  No — | | | — | |
| Yes 5.53 | | | 2.31, 15.5 | <0.001 |
| AKI | 183 | | | |
| No | — — | | | |
| Yes | 11.9 | | 6.05, 24.5 | <0.001 |
| This retrospective observational cohort study was conducted at Na Homolce Hospital, Prague, Czech Republic (January 2019–December 2022). We included consecutive critically ill ICU patients (N = 377) treated with meropenem, cefepime, or piperacillin–tazobactam for acute infections. Antibiotic plasma trough concentrations (Cmin) were measured after 24 h of full dosing using LC–MS/MS. Antibiotic concentrations were related to breakpoints obtained from the European Committee on Antimicrobial Susceptibility Testing (EUCAST). The MIC values for meropenem, cefepime and piperacillin were set at 8 mg/l, 8 mg/l, and 16 mg/l, respectively. Patients who were receiving an every-8-hour dosing regimen were included in subanalysis. Sample size N = 183. Univariate logistic regression. Data are presented as OR (odds ratio), 95 % CI (confidence interval) and p-value. BMI - Body mass index; GFR – glomerular filtration rate, CRRT – continuous renal replacement therapy, AKI - acute kidney injury, OR – odds ratio | | | | |

9g Results stratification according to dose number at time of level collection: Factors associated with underdosing (Cmin < 4x MIC) in patients receiving an every 8-hour dosing regimen (i.e 3 doses/24 hours) (Cmin < 1x MIC), Multivariate logistic regression analysis^1^

| characteristics | OR | conf.low | conf.high | p.value |
| --- | --- | --- | --- | --- |
| Age | 1.016 | 0.982 | 1.052 | 0.347 |
| Sex M | 1.145 | 0.489 | 2.654 | 0.753 |
| BMI | 1.020 | 0.952 | 1.094 | 0.571 |
| Vasopressor |  |  |  | 0.213 |
| Vasopressor low | 2.426 | 0.902 | 6.869 |  |
| Vasopressor medium | 1.132 | 0.397 | 3.270 |  |
| Vasopressor high | 2.686 | 0.713 | 12.488 |  |
| GFR_Jelliffe | 0.941 | 0.917 | 0.961 | 0.000 |
| CRRT | 2.035 | 0.700 | 6.587 | 0.196 |
| This retrospective observational cohort study was conducted at Na Homolce Hospital, Prague, Czech Republic (January 2019–December 2022). We included consecutive critically ill ICU patients (N = 377) treated with meropenem, cefepime, or piperacillin–tazobactam for acute infections. Antibiotic plasma trough concentrations (Cmin) were measured after 24 h of full dosing using LC–MS/MS. Antibiotic concentrations were related to breakpoints obtained from the European Committee on Antimicrobial Susceptibility Testing (EUCAST). The MIC values for meropenem, cefepime and piperacillin were set at 8 mg/l, 8 mg/l, and 16 mg/l, respectively. Patients who were receiving an every-8-hour dosing regimen were included in subanalysis. Sample size N = 183. BMI – body mass index, CRRT - continuous renal replacement therapy, GFR – glomerular filtration rate, OR = Odds Ratio  **^1^ Overall model fit: LR χ² (-8) = 81.61, p = 2.32e-14; Nagelkerke R² = 0.481; AUC = 0.865 (95% CI 0.813–0.917); Hosmer–Lemeshow χ² (8) = 14.32, p = 0.0737.** | | | | |

9h Results stratification according to dose number at time of level collection: Factors associated with overdosing (Cmin > 10x MIC) in patients receiving an every 8-hour dosing regimen (i.e 3 doses/24 hours) (Cmin < 1x MIC), Exploratory analysis

| **Characteristic** | | **N** | **<=10**  N = 151 | **>10**  N = 32 | | **Test statistic (df/N)** | **p-value^1^** |
| --- | --- | --- | --- | --- | --- | --- | --- |
| Age | | 183 | 67 (56, 73) | 70 (66, 79) | | U=1709.5; N=151/32 | 0.009^1^ |
| Sex | | 183 |  |  | | **χ²**=2.25; df=1 | 0.184^2^ |
| F | |  | 43.0 (28.5%) | 5.0 (15.6%) | |  |  |
| M | |  | 108.0 (71.5%) | 27.0 (84.4%) | |  |  |
| Weight | | 183 | 89 (80, 99) | 89 (74, 104) | | U=2303.5; N=151/32 | 0.6811^1^ |
| Height | | 183 | 175 (168, 180) | 171 (168, 175) | | U=2740.5; N=151/32 | 0.233^1^ |
| BMI | | 183 | 28.7 (25.7, 33.3) | 29.6 (25.8, 33.4) | | U=2279; N=151/32 | 0.616^1^ |
| Vasopressor | | 183 |  |  | |  | 0.697^3^ |
| none | | 76.0 (50.3%) | |  | 14.0 (43.8%) | | |
| low | | 33.0 (21.9%) | |  | 9.0 (28.1%) | | |
| medium | | 25.0 (16.6%) | |  | 4.0 (12.5%) | | |
| high | | 17.0 (11.3%) | |  | 5.0 (15.6%) | | |
| GFR_Jelliffe | | 183 | 39 (30, 62) | 20 (13, 29) | | U=4128; N=151/32 | <0.001^1^ |
| GFR_Jelliffe | | 183 |  |  | |  | <0.001^3^ |
| >120 | |  | 10.0 (6.6%) | 0.0 (0.0%) | |  |  |
| 48–120 | |  | 54.0 (35.8%) | 1.0 (3.1%) | |  |  |
| 12–48 | |  | 54.0 (35.8%) | 21.0 (65.6%) | |  |  |
| <12 | |  | 2.0 (1.3%) | 6.0 (18.8%) | |  |  |
| CRRT | |  | 31.0 (20.5%) | 4.0 (12.5%) | | χ²=1.1; df=1 |  |
| CRRT | | 183 |  |  | |  | 0.457^2^ |
| No | | 120.0 (79.5%) | |  | 28.0 (87.5%) | | |
| Yes | | 31.0 (20.5%) | |  | 4.0 (12.5%) | | |
| AKI | | 183 | 72.0 (47.7%) | 29.0 (90.6%) | | χ²=19.69; df=1 | <0.001^2^ |
|  | This retrospective observational cohort study was conducted at Na Homolce Hospital, Prague, Czech Republic (January 2019–December 2022). We included consecutive critically ill ICU patients (N = 377) treated with meropenem, cefepime, or piperacillin–tazobactam for acute infections. Antibiotic plasma trough concentrations (Cmin) were measured after 24 h of full dosing using LC–MS/MS. Antibiotic concentrations were related to breakpoints obtained from the European Committee on Antimicrobial Susceptibility Testing (EUCAST). The MIC values for meropenem, cefepime and piperacillin were set at 8 mg/l, 8 mg/l, and 16 mg/l, respectively. Patients who were receiving an every-8-hour dosing regimen were included in subanalysis. Sample size N = 183. Categorical variables presented as N (%). Continuous variables presented as median (Q1, Q3). BMI - Body mass index; GFR – glomerular filtration rate, CRRT – continuous renal replacement therapy, AKI - acute kidney injury  1 Mann–Whitney U test; ^2^Pearson’s Chi-squared test; ^2^Fisher’s exact test | | | | | | |

####

9i Results stratification according to dose number at time of level collection: Factors associated with overdosing (Cmin > 10x MIC) in patients receiving an every 8-hour dosing regimen (i.e 3 doses/24 hours) (Cmin < 1x MIC), Univariate logistic regression analysis

| **Characteristic** | **N** | **OR** | **95% CI** | **p-value** |
| --- | --- | --- | --- | --- |
| Age | 183 | 1.05 | 1.02, 1.10 | 0.009 |
| Sex | 183 |  |  |  |
| F |  | — | — |  |
| M |  | 2.15 | 0.84, 6.66 | 0.140 |
| Weight | 183 | 1.00 | 0.98, 1.02 | 0.987 |
| Height | 183 | 0.97 | 0.93, 1.01 | 0.190 |
| BMI | 183 | 1.02 | 0.95, 1.09 | 0.556 |
| Vasopressor | 183 | | | |
| none | — — | | | |
| low | 1.48 | | 0.57, 3.72 | 0.409 |
| medium | 0.87 | | 0.23, 2.69 | 0.818 |
| high | 1.60 | | 0.47, 4.83 | 0.425 |
| GFR_Jelliffe | 183 | 0.90 | 0.86, 0.94 | <0.001 |
| CRRT 183  No — | | | — | |
| Yes 0.55 | | | 0.16, 1.54 | 0.300 |
| AKI | 183 | | | |
| No | — — | | | |
| Yes | 10.6 | | 3.57, 45.6 | <0.001 |
| This retrospective observational cohort study was conducted at Na Homolce Hospital, Prague, Czech Republic (January 2019–December 2022). We included consecutive critically ill ICU patients (N = 377) treated with meropenem, cefepime, or piperacillin–tazobactam for acute infections. Antibiotic plasma trough concentrations (Cmin) were measured after 24 h of full dosing using LC–MS/MS. Antibiotic concentrations were related to breakpoints obtained from the European Committee on Antimicrobial Susceptibility Testing (EUCAST). The MIC values for meropenem, cefepime and piperacillin were set at 8 mg/l, 8 mg/l, and 16 mg/l, respectively. Patients who were receiving an every-8-hour dosing regimen were included in subanalysis. Sample size N = 183. Univariate logistic regression. Data are presented as OR (odds ratio), 95 % CI (confidence interval) and p-value. BMI - Body mass index; GFR – glomerular filtration rate, CRRT – continuous renal replacement therapy, AKI - acute kidney injury, CI = Confidence Interval, OR = Odds Ratio | | | | |

9j Results stratification according to dose number at time of level collection: Factors associated with overdosing (Cmin > 10x MIC) in patients receiving an every 8-hour dosing regimen (i.e 3 doses/24 hours) (Cmin < 1x MIC), Multivariate logistic regression analysis^1^

| characteristics | OR | conf.low | conf.high | p.value |
| --- | --- | --- | --- | --- |
| Age | 1.015 | 0.972 | 1.064 | 0.517 |
| Sex M | 2.364 | 0.742 | 9.060 | 0.151 |
| BMI | 1.066 | 0.976 | 1.174 | 0.159 |
| Vasopressor |  |  |  | 0.681 |
| Vasopressor low | 1.867 | 0.547 | 6.427 |  |
| Vasopressor medium | 0.804 | 0.183 | 3.071 |  |
| Vasopressor high | 1.059 | 0.233 | 4.468 |  |
| GFR_Jelliffe | 0.907 | 0.864 | 0.942 | 0.000 |
| CRRT | 0.436 | 0.109 | 1.468 | 0.186 |
| This retrospective observational cohort study was conducted at Na Homolce Hospital, Prague, Czech Republic (January 2019–December 2022). We included consecutive critically ill ICU patients (N = 377) treated with meropenem, cefepime, or piperacillin–tazobactam for acute infections. Antibiotic plasma trough concentrations (Cmin) were measured after 24 h of full dosing using LC–MS/MS. Antibiotic concentrations were related to breakpoints obtained from the European Committee on Antimicrobial Susceptibility Testing (EUCAST). The MIC values for meropenem, cefepime and piperacillin were set at 8 mg/l, 8 mg/l, and 16 mg/l, respectively. Patients who were receiving an every-8-hour dosing regimen were included in subanalysis. Sample size N = 183. BMI – body mass index, CRRT - continuous renal replacement therapy, GFR – glomerular filtration rate, OR = Odds Ratio  **^1^ Overall model fit: LR χ² (-8) = 53.49, p = 8.68e-09; Nagelkerke R² = 0.419; AUC = 0.87 (95% CI 0.794– 0.947); Hosmer–Lemeshow χ² (8) = 14.63, p = 0.0667** | | | | |

**Table S10** (a-j) Results stratification according to dose number at time of level collection: Dose regimen every 6 hours (i.e. 4 doses/24 hours)

10a Results stratification according to dose number at time of level collection: Achieved plasma concentrations in patients receiving dose regimen every 6 hours (i.e. 4 doses/24 hours)

| MIC | N | % |
| --- | --- | --- |
| 1-10 | 102 | 64.2% |
| >10 | 36 | 22.6% |
| <1 | 21 | 13.2% |
| This retrospective observational cohort study was conducted at Na Homolce Hospital, Prague, Czech Republic (January 2019–December 2022). We included consecutive critically ill ICU patients (N = 377) treated with meropenem, cefepime, or piperacillin–tazobactam for acute infections. Antibiotic plasma trough concentrations (Cmin) were measured after 24 h of full dosing using LC–MS/MS. Antibiotic concentrations were related to breakpoints obtained from the European Committee on Antimicrobial Susceptibility Testing (EUCAST). The MIC values for meropenem, cefepime and piperacillin were set at 8 mg/l, 8 mg/l, and 16 mg/l, respectively. Patients who were receiving an every-6-hour dosing regimen were included in subanalysis. Data are presented as absolute and relative frequencies. | | |

| MIC | N | % |
| --- | --- | --- |
| 4-10 | 64 | 40.3% |
| <4 | 59 | 37.1% |
| >10 | 36 | 22.6% |
| This retrospective observational cohort study was conducted at Na Homolce Hospital, Prague, Czech Republic (January 2019–December 2022). We included consecutive critically ill ICU patients (N = 377) treated with meropenem, cefepime, or piperacillin–tazobactam for acute infections. Antibiotic plasma trough concentrations (Cmin) were measured after 24 h of full dosing using LC–MS/MS. Antibiotic concentrations were related to breakpoints obtained from the European Committee on Antimicrobial Susceptibility Testing (EUCAST). The MIC values for meropenem, cefepime and piperacillin were set at 8 mg/l, 8 mg/l, and 16 mg/l, respectively. Patients who were receiving an every-6-hour dosing regimen were included in subanalysis. Data are presented as absolute and relative frequencies. | | |

10b Results stratification according to dose number at time of level collection: Factors associated with underdosing (Cmin < 1x MIC) in patients receiving dose regimen every 6 hours (i.e. 4 doses/24 hours). Exploratory analysis

| **Characteristic** | | **N** | **<1**  N = 21 | **>=1**  N = 138 | | | Test statistic (df/N) | **p-value^1^** |
| --- | --- | --- | --- | --- | --- | --- | --- | --- |
| Age | | 159 | 65 (59, 68) | 73 (67, 77) | | | U=568.5; N=21/138 | <0.001^1^ |
| Sex | | 159 |  |  | | |  | >0.999^2^ |
| F | |  | 7.0 (33.3%) | 45.0 (32.6%) | | | χ²=0; df=1 |  |
| M | |  | 14.0 (66.7%) | 93.0 (67.4%) | | |  |  |
| Weight | | 159 | 84 (75, 90) | 81 (71, 90) | | | U=1557.5; N=21/138 | 0.582^1^ |
| Height | | 159 | 174 (160, 183) | 171 (165, 179) | | | U=1579.5; N=21/138 | 0.508^1^ |
| BMI | | 159 | 27.8 (24.7, 33.6) | 27.4 (24.5, 30.5) | | | U=1508.5; N=21/138 | 0.764^1^ |
| Vasopressor | | 159 |  |  | | |  | 0.151^3^ |
| none | | 12.0 (57.1%) | |  | 53.0 (38.4%) | | | |
| low | | 7.0 (33.3%) | |  | 39.0 (28.3%) | | | |
| medium | | 2.0 (9.5%) | |  | 30.0 (21.7%) | | | |
| high | | 0.0 (0.0%) | |  | 16.0 (11.6%) | | | |
| GFR_Jelliffe | | 159 | 66 (36, 81) | 30 (19, 34) | | U=2452; N=21/138 | | <0.001^1^ |
| GFR_Jelliffe5 | | 159 |  |  | |  | | <0.001^3^ |
| >120 | |  | 2.0 (9.5%) | 0.0 (0.0%) | |  | |  |
| 48–120 | |  | 12.0 (57.1%) | 15.0 (10.9%) | |  | |  |
| 12–48 | |  | 6.0 (28.6%) | 72.0 (52.2%) | |  | |  |
| <12 | |  | 0.0 (0.0%) | 19.0 (13.8%) | |  | |  |
| CRRT | |  | 1.0 (4.8%) | 32.0 (23.2%) | |  | |  |
| CRRT | | 159 |  |  | |  | | 0.079^3^ |
| No | | 20.0 (95.2%) | |  | 106.0 (76.8%) | | | |
| Yes | | 1.0 (4.8%) | |  | 32.0 (23.2%) | | | |
| AKI | | 159 | 5.0 (23.8%) | 105.0 (76.1%) | | χ²=23.36; df=1 | | <0.001^2^ |
|  | This retrospective observational cohort study was conducted at Na Homolce Hospital, Prague, Czech Republic (January 2019–December 2022). We included consecutive critically ill ICU patients (N = 377) treated with meropenem, cefepime, or piperacillin–tazobactam for acute infections. Antibiotic plasma trough concentrations (Cmin) were measured after 24 h of full dosing using LC–MS/MS. Antibiotic concentrations were related to breakpoints obtained from the European Committee on Antimicrobial Susceptibility Testing (EUCAST). The MIC values for meropenem, cefepime and piperacillin were set at 8 mg/l, 8 mg/l, and 16 mg/l, respectively. Patients who were receiving an every-6-hour dosing regimen were included in subanalysis. Sample size N = 159. Categorical variables presented as N (%). Continuous variables presented as median (Q1, Q3). BMI - Body mass index; GFR – glomerular filtration rate, CRRT – continuous renal replacement therapy, AKI - acute kidney injury  1 Mann–Whitney U test; ^2^Pearson’s Chi-squared test; ^2^Fisher’s exact test | | | | | | | |

10c Results stratification according to dose number at time of level collection: Factors associated with underdosing (Cmin < 1x MIC) in patients receiving dose regimen every 6 hours (i.e. 4 doses/24 hours). Univariate logistic regression analysis

| **Characteristic** | **N** | **OR** | **95% CI** | **p-value** |
| --- | --- | --- | --- | --- |
| Age | 159 | 1.05 | 1.02, 1.09 | 0.004 |
| Sex | 159 |  |  |  |
| F |  | — | — |  |
| M |  | 1.03 | 0.37, 2.67 | 0.947 |
| Weight | 159 | 0.98 | 0.96, 1.01 | 0.274 |
| Height | 159 | 0.97 | 0.93, 1.02 | 0.295 |
| BMI | 159 | 0.98 | 0.89, 1.08 | 0.628 |
| Vasopressor | 159 | | | |
| none | — — | | | |
| low | 1.26 | | 0.46, 3.66 | 0.655 |
| medium | 3.40 | | 0.85, 22.8 | 0.125 |
| high | 26,184,632 | | 0.00, | 0.992 |
| GFR_Jelliffe | 159 | 0.93 | 0.90, 0.96 | <0.001 |
| CRRT 159  No — | | | — | |
| Yes 6.04 | | | 1.18, 110 | 0.085 |
| AKI | 159 | | | |
| No | — — | | | |
| Yes | 10.2 | | 3.68, 33.1 | <0.001 |
| This retrospective observational cohort study was conducted at Na Homolce Hospital, Prague, Czech Republic (January 2019–December 2022). We included consecutive critically ill ICU patients (N = 377) treated with meropenem, cefepime, or piperacillin–tazobactam for acute infections. Antibiotic plasma trough concentrations (Cmin) were measured after 24 h of full dosing using LC–MS/MS. Antibiotic concentrations were related to breakpoints obtained from the European Committee on Antimicrobial Susceptibility Testing (EUCAST). The MIC values for meropenem, cefepime and piperacillin were set at 8 mg/l, 8 mg/l, and 16 mg/l, respectively. Patients who were receiving an every-6-hour dosing regimen were included in subanalysis. Sample size N = 159. Univariate logistic regression. Data are presented as OR (odds ratio), 95 % CI (confidence interval) and p-value. BMI - Body mass index; GFR – glomerular filtration rate, CRRT – continuous renal replacement therapy, AKI - acute kidney injury | | | | |

10d Results stratification according to dose number at time of level collection: Factors associated with underdosing (Cmin < 1x MIC) in patients receiving dose regimen every 6 hours (i.e. 4 doses/24 hours). Multivariate logistic regression analysis^1^

| characteristics | OR | conf.low | conf.high | p.value |
| --- | --- | --- | --- | --- |
| Age | 1.000 | 0.940 | 1.067 | 0.991 |
| Sex M | 1.138 | 0.308 | 3.873 | 0.838 |
| BMI | 0.989 | 0.864 | 1.132 | 0.876 |
| Vasopressor |  |  |  | 0.683 |
| Vasopressor low | 0.781 | 0.205 | 3.013 |  |
| Vasopressor medium | 0.676 | 0.109 | 5.709 |  |
| Vasopressor high | 4073150.477 | 0.000 | NA |  |
| GFR | 0.938 | 0.902 | 0.969 | 0.000 |
| CRRT | 2.630 | 0.358 | 54.712 | 0.373 |
| This retrospective observational cohort study was conducted at Na Homolce Hospital, Prague, Czech Republic (January 2019–December 2022). We included consecutive critically ill ICU patients (N = 377) treated with meropenem, cefepime, or piperacillin–tazobactam for acute infections. Antibiotic plasma trough concentrations (Cmin) were measured after 24 h of full dosing using LC–MS/MS. Antibiotic concentrations were related to breakpoints obtained from the European Committee on Antimicrobial Susceptibility Testing (EUCAST). The MIC values for meropenem, cefepime and piperacillin were set at 8 mg/l, 8 mg/l, and 16 mg/l, respectively. Patients who were receiving an every-6-hour dosing regimen were included in subanalysis. Sample size N = 159. BMI – body mass index, CRRT - continuous renal replacement therapy, GFR – glomerular filtration rate, OR = Odds Ratio  **^1^Overall model fit: LR χ² (-8) = 42.76, p = 9.73e-07; Nagelkerke R² = 0.435; AUC = 0.875 (95% CI 0.792–0.958); Hosmer–Lemeshow χ² (8) = 8.45, p = 0.391.** | | | | |

10e Results stratification according to dose number at time of level collection: Factors associated with underdosing (Cmin < 4x MIC) in patients receiving dose regimen every 6 hours (i.e. 4 doses/24 hours). Exploratory analysis

| **Characteristic** | | **N** | **<4**  N = 59 | **>=4**  N = 100 | | Test statistic (df/N) | **p-value** |
| --- | --- | --- | --- | --- | --- | --- | --- |
| Age | | 159 | 67 (62, 74) | 73 (70, 77) | | U=1815; N=59/100 | <0.001^1^ |
| Sex | | 159 |  |  | |  | 0.223^2^ |
| F | |  | 23.0 (39.0%) | 29.0 (29.0%) | | χ²=1.68; df=1 |  |
| M | |  | 36.0 (61.0%) | 71.0 (71.0%) | |  |  |
| Weight | | 159 | 80 (72, 89) | 83 (72, 90) | | U=2705; N=59/100 | 0.383^1^ |
| Height | | 159 | 174 (160, 181) | 171 (167, 179) | | U=2978; N=59/100 | 0.929^1^ |
| BMI | | 159 | 26.4 (23.7, 31.3) | 27.8 (24.7, 30.5) | | U=2704; N=59/100 | 0.381^1^ |
| Vasopressor | | 159 |  |  | | χ²=6.27; df=3 | 0.092^2^ |
| none | | 29.0 (49.2%) | |  | 36.0 (36.0%) | | |
| low | | 18.0 (30.5%) | |  | 28.0 (28.0%) | | |
| medium | | 10.0 (16.9%) | |  | 22.0 (22.0%) | | |
| high | | 2.0 (3.4%) | |  | 14.0 (14.0%) | | |
| GFR_Jelliffe | | 159 | 42 (30, 59) | 24 (16, 30) | | U=4634; N=59/100 | <0.001^1^ |
| GFR_Jelliffe | | 159 |  |  | |  | <0.001^3^ |
| >120 | |  | 2.0 (3.4%) | 0.0 (0.0%) | |  |  |
| 48–120 | |  | 21.0 (35.6%) | 6.0 (6.0%) | |  |  |
| 12–48 | |  | 22.0 (37.3%) | 56.0 (56.0%) | |  |  |
| <12 | |  | 2.0 (3.4%) | 17.0 (17.0%) | |  |  |
| CRRT | |  | 12.0 (20.3%) | 21.0 (21.0%) | |  |  |
| CRRT | | 159 |  |  | | χ²=0.01; df=1 | >0.999^2^ |
| No | | 47.0 (79.7%) | |  | 79.0 (79.0%) | | |
| Yes | | 12.0 (20.3%) | |  | 21.0 (21.0%) | | |
| AKI | | 159 | 28.0 (47.5%) | 82.0 (82.0%) | | χ²=20.77; df=1 | <0.001^2^ |
|  | This retrospective observational cohort study was conducted at Na Homolce Hospital, Prague, Czech Republic (January 2019–December 2022). We included consecutive critically ill ICU patients (N = 377) treated with meropenem, cefepime, or piperacillin–tazobactam for acute infections. Antibiotic plasma trough concentrations (Cmin) were measured after 24 h of full dosing using LC–MS/MS. Antibiotic concentrations were related to breakpoints obtained from the European Committee on Antimicrobial Susceptibility Testing (EUCAST). The MIC values for meropenem, cefepime and piperacillin were set at 8 mg/l, 8 mg/l, and 16 mg/l, respectively. Patients who were receiving an every-6-hour dosing regimen were included in subanalysis. Sample size N = 159. Categorical variables presented as N (%). Continuous variables presented as median (Q1, Q3). BMI - Body mass index; GFR – glomerular filtration rate, CRRT – continuous renal replacement therapy, AKI - acute kidney injury  1 Mann–Whitney U test; ^2^ Pearson’s Chi-squared test; ^3^ Fisher’s exact test | | | | | | |

10f Results stratification according to dose number at time of level collection: Factors associated with underdosing (Cmin < 4x MIC) in patients receiving dose regimen every 6 hours (i.e. 4 doses/24 hours). Univariate logistic regression analysis

| **Characteristic** | **N** | **OR** | **95% CI** | **p-value** |
| --- | --- | --- | --- | --- |
| Age | 159 | 1.06 | 1.02, 1.09 | 0.002 |
| Sex | 159 |  |  |  |
| F |  | — | — |  |
| M |  | 1.56 | 0.79, 3.09 | 0.196 |
| Weight | 159 | 1.00 | 0.98, 1.03 | 0.700 |
| Height | 159 | 1.00 | 0.97, 1.03 | 0.957 |
| BMI | 159 | 1.01 | 0.94, 1.09 | 0.739 |
| Vasopressor | 159 | | | |
| none | — — | | | |
| low | 1.25 | | 0.58, 2.72 | 0.565 |
| medium | 1.77 | | 0.74, 4.46 | 0.209 |
| high | 5.64 | | 1.42, 37.8 | 0.030 |
| GFR_Jelliffe | 159 | 0.93 | 0.91, 0.96 | <0.001 |
| CRRT 159  No — | | | — | |
| Yes 1.04 | | | 0.48, 2.36 | 0.921 |
| AKI | 159 | | | |
| No | — — | | | |
| Yes | 5.04 | | 2.48, 10.6 | <0.001 |
| This retrospective observational cohort study was conducted at Na Homolce Hospital, Prague, Czech Republic (January 2019–December 2022). We included consecutive critically ill ICU patients (N = 377) treated with meropenem, cefepime, or piperacillin–tazobactam for acute infections. Antibiotic plasma trough concentrations (Cmin) were measured after 24 h of full dosing using LC–MS/MS. Antibiotic concentrations were related to breakpoints obtained from the European Committee on Antimicrobial Susceptibility Testing (EUCAST). The MIC values for meropenem, cefepime and piperacillin were set at 8 mg/l, 8 mg/l, and 16 mg/l, respectively. Patients who were receiving an every-6-hour dosing regimen were included in subanalysis. Sample size N = 159. Univariate logistic regression. Data are presented as OR (odds ratio), 95 % CI (confidence interval) and p-value. BMI - Body mass index; GFR – glomerular filtration rate, CRRT – continuous renal replacement therapy, AKI - acute kidney injury | | | | |

10g Results stratification according to dose number at time of level collection: Factors associated with underdosing (Cmin < 4x MIC) in patients receiving dose regimen every 6 hours (i.e. 4 doses/24 hours). Multivariate logistic regression analysis**^1^**

| characteristics | OR | conf.low | conf.high | p.value |
| --- | --- | --- | --- | --- |
| Age | 1.017 | 0.974 | 1.063 | 0.432 |
| Sex M | 2.418 | 1.045 | 5.755 | 0.039 |
| BMI | 1.074 | 0.984 | 1.177 | 0.112 |
| Vasopressor |  |  |  | 0.543 |
| Vasopressor low | 1.390 | 0.524 | 3.766 |  |
| Vasopressor medium | 0.974 | 0.307 | 3.160 |  |
| Vasopressor high | 3.057 | 0.579 | 26.187 |  |
| GFR_Jelliffe | 0.931 | 0.901 | 0.958 | 0.000 |
| CRRT | 0.619 | 0.227 | 1.685 | 0.345 |
| This retrospective observational cohort study was conducted at Na Homolce Hospital, Prague, Czech Republic (January 2019–December 2022). We included consecutive critically ill ICU patients (N = 377) treated with meropenem, cefepime, or piperacillin–tazobactam for acute infections. Antibiotic plasma trough concentrations (Cmin) were measured after 24 h of full dosing using LC–MS/MS. Antibiotic concentrations were related to breakpoints obtained from the European Committee on Antimicrobial Susceptibility Testing (EUCAST). The MIC values for meropenem, cefepime and piperacillin were set at 8 mg/l, 8 mg/l, and 16 mg/l, respectively. Patients who were receiving an every-6-hour dosing regimen were included in subanalysis. Sample size N = 159. BMI – body mass index, CRRT - continuous renal replacement therapy, GFR – glomerular filtration rate, OR = Odds Ratio  **1 Overall model fit: LR χ² (-8) = 55.05, p = 4.32e-09; Nagelkerke R² = 0.399; AUC = 0.828 (95% CI 0.761–0.894); Hosmer–Lemeshow χ² (8) = 4.01, p = 0.857.** | | | | |

10h Results stratification according to dose number at time of level collection: Factors associated with overdosing (Cmin > 10x MIC) in patients receiving dose regimen every 6 hours (i.e. 4 doses/24 hours). Exploratory analysis.

| **Characteristic** | | **N** | **<=10**  N = 123 | **>10**  N = 36 | | Test statistic (df/N) | | **p-value** |
| --- | --- | --- | --- | --- | --- | --- | --- | --- |
| Age | | 159 | 71 (65, 75) | 73 (71, 79) | | U=1684.5; N=123/36 | | 0.029^1^ |
| Sex | | 159 |  |  | | χ²=0.1; df=1 | | 0.841^2^ |
| F | |  | 41.0 (33.3%) | 11.0 (30.6%) | |  | |  |
| M | |  | 82.0 (66.7%) | 25.0 (69.4%) | |  | |  |
| Weight | | 159 | 80 (72, 90) | 85 (75, 91) | | U=2015; N=123/36 | | 0.414^1^ |
| Height | | 159 | 172 (164, 180) | 171 (168, 177) | | U=2228; N=123/36 | | 0.956^1^ |
| BMI | | 159 | 27.2 (24.5, 31.0) | 28.7 (24.6, 31.1) | | U=1998; N=123/36 | | 0.375^1^ |
| Vasopressor | | 159 |  |  | |  | | 0.758^3^ |
| none | | 51.0 (41.5%) | |  | 14.0 (38.9%) | | | |
| low | | 37.0 (30.1%) | |  | 9.0 (25.0%) | | | |
| medium | | 24.0 (19.5%) | |  | 8.0 (22.2%) | | | |
| high | | 11.0 (8.9%) | |  | 5.0 (13.9%) | | | |
| GFR_Jelliffe | | 159 | 30 (21, 47) | 17 (11, 30) | | U=3378; N=123/36 | | <0.001^1^ |
| GFR_Jelliffe | | 159 |  |  | |  | | <0.001^3^ |
| >120 | |  | 2.0 (1.6%) | 0.0 (0.0%) | |  | |  |
| 48–120 | |  | 27.0 (22.0%) | 0.0 (0.0%) | |  | |  |
| 12–48 | |  | 61.0 (49.6%) | 17.0 (47.2%) | |  | |  |
| <12 | |  | 8.0 (6.5%) | 11.0 (30.6%) | |  | |  |
| CRRT | |  | 25.0 (20.3%) | 8.0 (22.2%) | |  | |  |
| CRRT | | 159 |  |  | | χ²=0.06; df=1 | | 0.817^2^ |
| No | | 98.0 (79.7%) | |  | 28.0 (77.8%) | | | |
| Yes | | 25.0 (20.3%) | |  | 8.0 (22.2%) | | | |
| AKI | | 159 | 75.0 (61.0%) | 35.0 (97.2%) | | | χ²=17.16; df=1 | <0.001^2^ |
|  | This retrospective observational cohort study was conducted at Na Homolce Hospital, Prague, Czech Republic (January 2019–December 2022). We included consecutive critically ill ICU patients (N = 377) treated with meropenem, cefepime, or piperacillin–tazobactam for acute infections. Antibiotic plasma trough concentrations (Cmin) were measured after 24 h of full dosing using LC–MS/MS. Antibiotic concentrations were related to breakpoints obtained from the European Committee on Antimicrobial Susceptibility Testing (EUCAST). The MIC values for meropenem, cefepime and piperacillin were set at 8 mg/l, 8 mg/l, and 16 mg/l, respectively. Patients who were receiving an every-6-hour dosing regimen were included in subanalysis. Sample size N = 159. Categorical variables presented as N (%). Continuous variables presented as median (Q1, Q3). BMI - Body mass index; GFR – glomerular filtration rate, CRRT – continuous renal replacement therapy, AKI - acute kidney injury  1 Mann–Whitney U test; ^2^Pearson’s Chi-squared test; ^3^Fisher’s exact test | | | | | | | |

10i Results stratification according to dose number at time of level collection: Factors associated with overdosing (Cmin > 10x MIC) in patients receiving dose regimen every 6 hours (i.e. 4 doses/24 hours). Univariate logistic regression analysis.

| **Characteristic** | **N** | **OR** | **95% CI** | **p-value** |
| --- | --- | --- | --- | --- |
| Age | 159 | 1.04 | 1.00, 1.09 | 0.048 |
| Sex | 159 |  |  |  |
| F |  | — | — |  |
| M |  | 1.14 | 0.52, 2.61 | 0.755 |
| Weight | 159 | 1.01 | 0.98, 1.03 | 0.458 |
| Height | 159 | 1.00 | 0.96, 1.04 | 0.936 |
| BMI | 159 | 1.02 | 0.94, 1.11 | 0.567 |
| Vasopressor | 159 | | | |
| none | — — | | | |
| low | 0.89 | | 0.34, 2.24 | 0.801 |
| medium | 1.21 | | 0.43, 3.24 | 0.702 |
| high | 1.66 | | 0.46, 5.41 | 0.414 |
| GFR_Jelliffe | 159 | 0.93 | 0.89, 0.96 | <0.001 |
| CRRT 159  No — | | | — | |
| Yes 1.12 | | | 0.43, 2.67 | 0.805 |
| AKI | 159 | | | |
| No | — — | | | |
| Yes | 22.4 | | 4.59, 405 | 0.003 |
| This retrospective observational cohort study was conducted at Na Homolce Hospital, Prague, Czech Republic (January 2019–December 2022). We included consecutive critically ill ICU patients (N = 377) treated with meropenem, cefepime, or piperacillin–tazobactam for acute infections. Antibiotic plasma trough concentrations (Cmin) were measured after 24 h of full dosing using LC–MS/MS. Antibiotic concentrations were related to breakpoints obtained from the European Committee on Antimicrobial Susceptibility Testing (EUCAST). The MIC values for meropenem, cefepime and piperacillin were set at 8 mg/l, 8 mg/l, and 16 mg/l, respectively. Patients who were receiving an every-6-hour dosing regimen were included in subanalysis. Sample size N = 159. Univariate logistic regression. Data are presented as OR (odds ratio), 95 % CI (confidence interval) and p-value. BMI - Body mass index; GFR – glomerular filtration rate, CRRT – continuous renal replacement therapy, AKI - acute kidney injury | | | | |

10j Results stratification according to dose number at time of level collection: Factors associated with overdosing (Cmin > 10x MIC) in patients receiving dose regimen every 6 hours (i.e. 4 doses/24 hours). Multivariate logistic regression analysis^1^

| characteristics | OR | conf.low | conf.high | p.value |
| --- | --- | --- | --- | --- |
| Age | 0.992 | 0.943 | 1.046 | 0.753 |
| Sex M | 1.893 | 0.757 | 5.101 | 0.176 |
| BMI | 1.078 | 0.986 | 1.182 | 0.100 |
| Vasopressor |  |  |  | 0.867 |
| Vasopressor low | 0.888 | 0.291 | 2.652 |  |
| Vasopressor medium | 0.637 | 0.187 | 2.074 |  |
| Vasopressor high | 0.657 | 0.149 | 2.625 |  |
| GFR_Jelliffe | 0.899 | 0.845 | 0.944 | <0.001 |
| CRRT | 1.899 | 0.579 | 6.384 | 0.287 |
| This retrospective observational cohort study was conducted at Na Homolce Hospital, Prague, Czech Republic (January 2019–December 2022). We included consecutive critically ill ICU patients (N = 377) treated with meropenem, cefepime, or piperacillin–tazobactam for acute infections. Antibiotic plasma trough concentrations (Cmin) were measured after 24 h of full dosing using LC–MS/MS. Antibiotic concentrations were related to breakpoints obtained from the European Committee on Antimicrobial Susceptibility Testing (EUCAST). The MIC values for meropenem, cefepime and piperacillin were set at 8 mg/l, 8 mg/l, and 16 mg/l, respectively. Patients who were receiving an every-6-hour dosing regimen were included in subanalysis. Sample size N = 159. BMI – body mass index, CRRT - continuous renal replacement therapy, GFR – glomerular filtration rate, OR = Odds Ratio  **^1^ Overall model fit: LR χ² (-8) = 33.34, p = 5.36e-05; Nagelkerke R² = 0.288; AUC = 0.8 (95% CI 0.721– 0.879); Hosmer–Lemeshow χ² (8) = 5.15, p = 0.742.** | | | | |

**Table S11 (a-j)** Dose regimen every 4 hours (i.e. 6 doses/24 hours)

11a Results stratification according to dose number at time of level collection: Achieved plasma concentrations in patients receiving an every 4-hour dosing regimen (i.e. 6 doses/24 hours)

| MIC | N | % |
| --- | --- | --- |
| 1-10 | 23 | 65.7% |
| <1 | 6 | 17.1% |
| >10 | 6 | 17.1% |
| This retrospective observational cohort study was conducted at Na Homolce Hospital, Prague, Czech Republic (January 2019–December 2022). We included consecutive critically ill ICU patients (N = 377) treated with meropenem, cefepime, or piperacillin–tazobactam for acute infections. Antibiotic plasma trough concentrations (Cmin) were measured after 24 h of full dosing using LC–MS/MS. Antibiotic concentrations were related to breakpoints obtained from the European Committee on Antimicrobial Susceptibility Testing (EUCAST). The MIC values for meropenem, cefepime and piperacillin were set at 8 mg/l, 8 mg/l, and 16 mg/l, respectively. Patients who were receiving an every-4-hour dosing regimen were included in subanalysis. Data are presented as absolute and relative frequencies. | | |

| MIC | N | % |
| --- | --- | --- |
| <4 | 17 | 48.6% |
| 4-10 | 12 | 34.3% |
| >10 | 6 | 17.1% |
| This retrospective observational cohort study was conducted at Na Homolce Hospital, Prague, Czech Republic (January 2019–December 2022). We included consecutive critically ill ICU patients (N = 377) treated with meropenem, cefepime, or piperacillin–tazobactam for acute infections. Antibiotic plasma trough concentrations (Cmin) were measured after 24 h of full dosing using LC–MS/MS. Antibiotic concentrations were related to breakpoints obtained from the European Committee on Antimicrobial Susceptibility Testing (EUCAST). The MIC values for meropenem, cefepime and piperacillin were set at 8 mg/l, 8 mg/l, and 16 mg/l, respectively. Patients who were receiving an every-4-hour dosing regimen were included in subanalysis. Data are presented as absolute and relative frequencies. | | |

11b Results stratification according to dose number at time of level collection: Factors associated with underdosing (Cmin < 1x MIC) in patients receiving an every 4-hour dosing regimen (i.e. 6 doses/24 hours). Exploratory analysis

| **Characteristic** | | **N** | **<1**  N = 6 | **>=1**  N = 29 | | Test statistic (df/N) | **p-value** |
| --- | --- | --- | --- | --- | --- | --- | --- |
| Age | | 35 | 61 (55, 62) | 67 (55, 72) | | U=56; N=6/29 | 0.181^1^ |
| Sex | | 35 |  |  | |  | 0.311^2^ |
| F | |  | 0.0 (0.0%) | 7.0 (24.1%) | |  |  |
| M | |  | 6.0 (100.0%) | 22.0 (75.9%) | |  |  |
| Weight | | 35 | 97.5 (88, 120) | 102 (94, 120) | | U=74.5; N=6/29 | 0.598^1^ |
| Height | | 35 | 190 (180, 205) | 178 (170, 186) | | U=148.5; N=6/29 | 0.007^1^ |
| BMI | | 35 | 28.1 (26.2, 28.6) | 33.6 (29.7, 37.9) | | U=22.5; N=6/29 | 0.005^1^ |
| Vasopressor | | 35 |  |  | |  | 0.251^3^ |
| none | | 5.0 (83.3%) | |  | 12.0 (41.4%) | | |
| low | | 0.0 (0.0%) | |  | 10.0 (34.5%) | | |
| medium | | 1.0 (16.7%) | |  | 4.0 (13.8%) | | |
| high | | 0.0 (0.0%) | |  | 3.0 (10.3%) | | |
| GFR_Jelliffe | | 35 | 89 (81, 94) | 45 (33, 83) | | U=116.5; N=6/29 | 0.204^1^ |
| GFR_Jelliffe | | 35 |  |  | |  | 0.072^3^ |
| >120 | |  | 0.0 (0.0%) | 3.0 (10.3%) | |  |  |
| 48–120 | |  | 5.0 (83.3%) | 10.0 (34.5%) | |  |  |
| 12–48 | |  | 0.0 (0.0%) | 13.0 (44.8%) | |  |  |
| <12 | |  | 0.0 (0.0%) | 0.0 (0.0%) | |  |  |
| CRRT | |  | 1.0 (16.7%) | 3.0 (10.3%) | |  |  |
| CRRT | | 35 |  |  | |  | 0.546^3^ |
| No | | 5.0 (83.3%) | |  | 26.0 (89.7%) | | |
| Yes | | 1.0 (16.7%) | |  | 3.0 (10.3%) | | |
| AKI | | 35 | 1.0 (16.7%) | 15.0 (51.7%) | |  | 0.186^3^ |
|  | This retrospective observational cohort study was conducted at Na Homolce Hospital, Prague, Czech Republic (January 2019–December 2022). We included consecutive critically ill ICU patients (N = 377) treated with meropenem, cefepime, or piperacillin–tazobactam for acute infections. Antibiotic plasma trough concentrations (Cmin) were measured after 24 h of full dosing using LC–MS/MS. Antibiotic concentrations were related to breakpoints obtained from the European Committee on Antimicrobial Susceptibility Testing (EUCAST). The MIC values for meropenem, cefepime and piperacillin were set at 8 mg/l, 8 mg/l, and 16 mg/l, respectively. Patients who were receiving an every-4-hour dosing regimen were included in subanalysis. Sample size N = 35. Categorical variables presented as N (%). Continuous variables presented as median (Q1, Q3). BMI - Body mass index; GFR – glomerular filtration rate, CRRT – continuous renal replacement therapy, AKI - acute kidney injury  1 Mann–Whitney U test; ^2^Pearson’s Chi-squared test; ^3^Fisher’s exact test | | | | | | |

11c Results stratification according to dose number at time of level collection: Factors associated with underdosing (Cmin < 1x MIC) in patients receiving an every 4-hour dosing regimen (i.e. 6 doses/24 hours). Univariate logistic regression analysis

| **Characteristic** | **N** | **OR** | **95% CI** | **p-value** |
| --- | --- | --- | --- | --- |
| Age | 35 | 1.03 | 0.96, 1.10 | 0.371 |
| Sex | 35 |  |  |  |
| F |  | — | — |  |
| M |  | 0.00 |  | 0.994 |
| Weight | 35 | 1.01 | 0.97, 1.06 | 0.584 |
| Height | 35 | 0.86 | 0.73, 0.95 | 0.019 |
| BMI | 35 | 1.41 | 1.09, 2.08 | 0.031 |
| Vasopressor | 35 | | | |
| none | — — | | | |
| low | 130,985,840 | | 0.00, | 0.996 |
| medium | 1.67 | | 0.18, 37.1 | 0.680 |
| high | 130,985,840 | | 0.00, | 0.998 |
| GFR_Jelliffe | 35 | 0.99 | 0.97, 1.01 | 0.287 |
| CRRT 35  No — | | | — | |
| Yes 0.58 | | | 0.06, 13.0 | 0.661 |
| AKI | 35 | | | |
| No | — — | | | |
| Yes | 5.36 | | 0.74, 110 | 0.147 |
| This retrospective observational cohort study was conducted at Na Homolce Hospital, Prague, Czech Republic (January 2019–December 2022). We included consecutive critically ill ICU patients (N = 377) treated with meropenem, cefepime, or piperacillin–tazobactam for acute infections. Antibiotic plasma trough concentrations (Cmin) were measured after 24 h of full dosing using LC–MS/MS. Antibiotic concentrations were related to breakpoints obtained from the European Committee on Antimicrobial Susceptibility Testing (EUCAST). The MIC values for meropenem, cefepime and piperacillin were set at 8 mg/l, 8 mg/l, and 16 mg/l, respectively. Patients who were receiving an every-4-hour dosing regimen were included in subanalysis. Sample size N = 35. Univariate logistic regression analysis. Data are presented as OR (odds ratio), 95 % CI (confidence interval) and p-value. BMI - Body mass index; GFR – glomerular filtration rate, CRRT – continuous renal replacement therapy, AKI - acute kidney injury | | | | |

11d Results stratification according to dose number at time of level collection: Factors associated with underdosing (Cmin < 1x MIC) in patients receiving an every 4-hour dosing regimen (i.e. 6 doses/24 hours). Multivariate logistic regression analysis – **insufficient data**

11e Results stratification according to dose number at time of level collection: Factors associated with underdosing (Cmin < 4x MIC) in patients receiving an every 4-hour dosing regimen (i.e. 6 doses/24 hours). Exploratory analysis.

| **Characteristic** | | **N** | **<4**  N = 17 | **>=4**  N = 18 | | Test statistic (df/N) | **p-value** |
| --- | --- | --- | --- | --- | --- | --- | --- |
| Age | | 35 | 61 (48, 63) | 71 (63, 74) | | U=61; N=17/18 | 0.002^1^ |
| Sex | | 35 |  |  | |  | 0.999^2^ |
| F | |  | 3.0 (17.6%) | 4.0 (22.2%) | |  |  |
| M | |  | 14.0 (82.4%) | 14.0 (77.8%) | |  |  |
| Weight | | 35 | 104 (90, 120) | 101 (92, 120) | | U=165; N=17/18 | 0.703^1^ |
| Height | | 35 | 180 (179, 188) | 173 (170, 187) | | U=214; N=17/18 | 0.045^1^ |
| BMI | | 35 | 29.7 (28.1, 37.0) | 32.9 (29.6, 34.9) | | U=128; N=17/18 | 0.419^1^ |
| Vasopressor | | 35 |  |  | |  | 0.197^3^ |
| none | | 10.0 (58.8%) | |  | 7.0 (38.9%) | | |
| low | | 2.0 (11.8%) | |  | 8.0 (44.4%) | | |
| medium | | 3.0 (17.6%) | |  | 2.0 (11.1%) | | |
| high | | 2.0 (11.8%) | |  | 1.0 (5.6%) | | |
| GFR_Jelliffe | | 35 | 89 (63, 101) | 36 (30, 46) | | U=253.5; N=17/18 | <0.001^1^ |
| GFR_Jelliffe | | 35 |  |  | |  | 0.002^3^ |
| >120 | |  | 2.0 (11.8%) | 1.0 (5.6%) | |  |  |
| 48–120 | |  | 12.0 (70.6%) | 3.0 (16.7%) | |  |  |
| 12–48 | |  | 2.0 (11.8%) | 11.0 (61.1%) | |  |  |
| <12 | |  | 0.0 (0.0%) | 0.0 (0.0%) | |  |  |
| CRRT | |  | 1.0 (5.9%) | 3.0 (16.7%) | |  |  |
| CRRT | | 35 |  |  | |  | 0.603^3^ |
| No | | 16.0 (94.1%) | |  | 15.0 (83.3%) | | |
| Yes | | 1.0 (5.9%) | |  | 3.0 (16.7%) | | |
| AKI | | 35 | 4.0 (23.5%) | 12.0 (66.7%) | | χ² =6.56; df=1 | 0.010^2^ |
|  | This retrospective observational cohort study was conducted at Na Homolce Hospital, Prague, Czech Republic (January 2019–December 2022). We included consecutive critically ill ICU patients (N = 377) treated with meropenem, cefepime, or piperacillin–tazobactam for acute infections. Antibiotic plasma trough concentrations (Cmin) were measured after 24 h of full dosing using LC–MS/MS. Antibiotic concentrations were related to breakpoints obtained from the European Committee on Antimicrobial Susceptibility Testing (EUCAST). The MIC values for meropenem, cefepime and piperacillin were set at 8 mg/l, 8 mg/l, and 16 mg/l, respectively. Patients who were receiving an every-4-hour dosing regimen were included in subanalysis. Sample size N = 35. Categorical variables presented as N (%). Continuous variables presented as median (Q1, Q3). BMI - Body mass index; GFR – glomerular filtration rate, CRRT – continuous renal replacement therapy, AKI - acute kidney injury  1 Mann–Whitney U test; ^2^ Pearson’s Chi-squared test; ^3^ Fisher’s exact test | | | | | | |

11f Results stratification according to dose number at time of level collection: Factors associated with underdosing (Cmin < 4x MIC) in patients receiving an every 4-hour dosing regimen (i.e. 6 doses/24 hours). Univariate logistic analysis.

| **Characteristic** | **N** | **OR** | **95% CI** | **p-value** |
| --- | --- | --- | --- | --- |
| Age | 35 | 1.10 | 1.03, 1.20 | 0.012 |
| Sex | 35 |  |  |  |
| F |  | — | — |  |
| M |  | 0.75 | 0.13, 4.02 | 0.736 |
| Weight | 35 | 1.00 | 0.97, 1.03 | 0.881 |
| Height | 35 | 0.95 | 0.88, 1.00 | 0.092 |
| BMI | 35 | 1.04 | 0.95, 1.17 | 0.432 |
| Vasopressor | 35 | | | |
| none | — — | | | |
| low | 5.71 | | 1.04, 46.2 | 0.061 |
| medium | 0.95 | | 0.10, 7.31 | 0.962 |
| high | 0.71 | | 0.03, 8.97 | 0.799 |
| GFR_Jelliffe | 35 | 0.95 | 0.92, 0.98 | 0.003 |
| CRRT 35  No — | | | — | |
| Yes 3.20 | | | 0.36, 68.7 | 0.336 |
| AKI | 35 | | | |
| No | — — | | | |
| Yes | 6.50 | | 1.57, 32.1 | 0.014 |
| This retrospective observational cohort study was conducted at Na Homolce Hospital, Prague, Czech Republic (January 2019–December 2022). We included consecutive critically ill ICU patients (N = 377) treated with meropenem, cefepime, or piperacillin–tazobactam for acute infections. Antibiotic plasma trough concentrations (Cmin) were measured after 24 h of full dosing using LC–MS/MS. Antibiotic concentrations were related to breakpoints obtained from the European Committee on Antimicrobial Susceptibility Testing (EUCAST). The MIC values for meropenem, cefepime and piperacillin were set at 8 mg/l, 8 mg/l, and 16 mg/l, respectively. Patients who were receiving an every-4-hour dosing regimen were included in subanalysis. Sample size N = 35. Univariate logistic regression. Data are presented as OR (odds ratio), 95 % CI (confidence interval) and p-value. BMI - Body mass index; GFR – glomerular filtration rate, CRRT – continuous renal replacement therapy, AKI - acute kidney injury | | | | |

11g Results stratification according to dose number at time of level collection: Factors associated with underdosing (Cmin < 4x MIC) in patients receiving an every 4-hour dosing regimen (i.e. 6 doses/24 hours). Multivariate logistic analysis^1^

| characteristics | OR | conf.low | conf.high | p.value |
| --- | --- | --- | --- | --- |
| Age | 1.064 | 0.954 | 1.199 | 0.266 |
| Sex M | 1.941 | 0.139 | 26.682 | 0.604 |
| BMI | 1.104 | 0.950 | 1.374 | 0.229 |
| Vasopressor |  |  |  | 0.301 |
| Vasopressor low | 8.371 | 0.750 | 183.875 |  |
| Vasopressor medium | 1.111 | 0.073 | 16.728 |  |
| Vasopressor high | 1.565 | 0.012 | 192.431 |  |
| GFR_Jelliffe | 0.961 | 0.915 | 0.998 | 0.041 |
| CRRT | 0.446 | 0.012 | 18.780 | 0.647 |
| This retrospective observational cohort study was conducted at Na Homolce Hospital, Prague, Czech Republic (January 2019–December 2022). We included consecutive critically ill ICU patients (N = 377) treated with meropenem, cefepime, or piperacillin–tazobactam for acute infections. Antibiotic plasma trough concentrations (Cmin) were measured after 24 h of full dosing using LC–MS/MS. Antibiotic concentrations were related to breakpoints obtained from the European Committee on Antimicrobial Susceptibility Testing (EUCAST). The MIC values for meropenem, cefepime and piperacillin were set at 8 mg/l, 8 mg/l, and 16 mg/l, respectively. Patients who were receiving an every-4-hour dosing regimen were included in subanalysis. Sample size N = 35. BMI – body mass index, CRRT - continuous renal replacement therapy, GFR – glomerular filtration rate, OR = Odds Ratio  **^1^ Overall model fit: LR χ² (-8) = 20.49, p = 0.00862; Nagelkerke R² = 0.591; AUC = 0.915 (95% CI 0.813–1); Hosmer–Lemeshow χ² (8) = 22.67, p = 0.00381.** | | | | |

11h Results stratification according to dose number at time of level collection: Factors associated with underdosing (Cmin > 10x MIC) in patients receiving an every 4-hour dosing regimen (i.e. 6 doses/24 hours). Exploratory analysis.

| **Characteristic** | | **N** | **<=10**  N = 29 | **>10**  N = 6 | | Test statistic (df/N) | **p-value** |
| --- | --- | --- | --- | --- | --- | --- | --- |
| Age | | 35 | 63 (55, 72) | 70 (67, 70) | | U=77; N=29/6 | 0.677^1^ |
| Sex | | 35 |  |  | |  | >0.999^3^ |
| F | |  | 6.0 (20.7%) | 1.0 (16.7%) | |  |  |
| M | |  | 23.0 (79.3%) | 5.0 (83.3%) | |  |  |
| Weight | | 35 | 102 (90, 120) | 107 (97, 120) | | U=68.5; N=29/6 | 0.4291 |
| Height | | 35 | 180 (170, 188) | 177 (171, 178) | | U=106; N=29/6 | 0.417^1^ |
| BMI | | 35 | 31.9 (28.4, 34.3) | 36.4 (30.4, 37.9) | | U=50; N=29/6 | 0.110^1^ |
| Vasopressor | | 35 |  |  | |  | 0.328^3^ |
| none | | 15.0 (51.7%) | |  | 2.0 (33.3%) | | |
| low | | 7.0 (24.1%) | |  | 3.0 (50.0%) | | |
| medium | | 5.0 (17.2%) | |  | 0.0 (0.0%) | | |
| high | | 2.0 (6.9%) | |  | 1.0 (16.7%) | | |
| GFR_Jelliffe | | 35 | 61 (43, 91) | 32 (30, 35) | | U=125; N=29/6 | 0.100^1^ |
| GFR_Jelliffe5 | | 35 |  |  | |  | 0.037^3^ |
| >120 | |  | 2.0 (6.9%) | 1.0 (16.7%) | |  |  |
| 48–120 | |  | 15.0 (51.7%) | 0.0 (0.0%) | |  |  |
| 12–48 | |  | 10.0 (34.5%) | 3.0 (50.0%) | |  |  |
| <12 | |  | 0.0 (0.0%) | 0.0 (0.0%) | |  |  |
| CRRT | |  | 2.0 (6.9%) | 2.0 (33.3%) | |  |  |
| CRRT | | 35 |  |  | |  | 0.128^3^ |
| No | | 27.0 (93.1%) | |  | 4.0 (66.7%) | | |
| Yes | | 2.0 (6.9%) | |  | 2.0 (33.3%) | | |
| AKI | | 35 | 11.0 (37.9%) | 5.0 (83.3%) | |  | 0.073^3^ |
|  | This retrospective observational cohort study was conducted at Na Homolce Hospital, Prague, Czech Republic (January 2019–December 2022). We included consecutive critically ill ICU patients (N = 377) treated with meropenem, cefepime, or piperacillin–tazobactam for acute infections. Antibiotic plasma trough concentrations (Cmin) were measured after 24 h of full dosing using LC–MS/MS. Antibiotic concentrations were related to breakpoints obtained from the European Committee on Antimicrobial Susceptibility Testing (EUCAST). The MIC values for meropenem, cefepime and piperacillin were set at 8 mg/l, 8 mg/l, and 16 mg/l, respectively. Patients who were receiving an every-4-hour dosing regimen were included in subanalysis. Sample size N = 35. Categorical variables presented as N (%). Continuous variables presented as median (Q1, Q3). BMI - Body mass index; GFR – glomerular filtration rate, CRRT – continuous renal replacement therapy, AKI - acute kidney injury  1 Mann–Whitney U test; ^2^ Pearson’s Chi-squared test; ^3^ Fisher’s exact test | | | | | | |

11i Results stratification according to dose number at time of level collection: Factors associated with underdosing (Cmin > 10x MIC) in patients receiving an every 4-hour dosing regimen (i.e. 6 doses/24 hours). Univariate logistic regression analysis.

| **Characteristic** | **N** | **OR** | **95% CI** | **p-value** |
| --- | --- | --- | --- | --- |
| Age | 35 | 1.03 | 0.96, 1.12 | 0.496 |
| Sex | 35 |  |  |  |
| F |  | — | — |  |
| M |  | 1.30 | 0.16, 27.5 | 0.823 |
| Weight | 35 | 1.01 | 0.97, 1.05 | 0.605 |
| Height | 35 | 0.97 | 0.89, 1.04 | 0.427 |
| BMI | 35 | 1.05 | 0.94, 1.18 | 0.352 |
| Vasopressor | 35 | | | |
| none | — — | | | |
| low | 3.21 | | 0.44, 29.0 | 0.253 |
| medium | 0.00 | |  | 0.995 |
| high | 3.75 | | 0.14, 65.5 | 0.358 |
| GFR_Jelliffe | 35 | 0.98 | 0.94, 1.01 | 0.216 |
| CRRT 35  No — | | | — | |
| Yes 6.75 | | | 0.66, 72.0 | 0.092 |
| AKI | 35 | | | |
| No | — — | | | |
| Yes | 8.18 | | 1.13, 168 | 0.070 |
| This retrospective observational cohort study was conducted at Na Homolce Hospital, Prague, Czech Republic (January 2019–December 2022). We included consecutive critically ill ICU patients (N = 377) treated with meropenem, cefepime, or piperacillin–tazobactam for acute infections. Antibiotic plasma trough concentrations (Cmin) were measured after 24 h of full dosing using LC–MS/MS. Antibiotic concentrations were related to breakpoints obtained from the European Committee on Antimicrobial Susceptibility Testing (EUCAST). The MIC values for meropenem, cefepime and piperacillin were set at 8 mg/l, 8 mg/l, and 16 mg/l, respectively. Patients who were receiving an every-4-hour dosing regimen were included in subanalysis. Sample size N = 35. Univariate logistic regression. Data are presented as OR (odds ratio), 95 % CI (confidence interval) and p-value. BMI - Body mass index; GFR – glomerular filtration rate, CRRT – continuous renal replacement therapy, AKI - acute kidney injury | | | | |

11j Results stratification according to dose number at time of level collection: Factors associated with underdosing (Cmin > 10x MIC) in patients receiving an every 4-hour dosing regimen (i.e. 6 doses/24 hours). Multivariate logistic regression analysis^1^.

| characteristics | OR | conf.low | conf.high | p.value |
| --- | --- | --- | --- | --- |
| Age | 1.070 | 0.930 | 1.250 | 0.329 |
| Sex M | 1.390 | 0.100 | 86.30 | 0.828 |
| BMI | 1.070 | 0.930 | 1.270 | 0.289 |
| Vasopressor |  |  |  | 0.251 |
| Vasopressor low | 1.550 | 0.050 | 31.3 |  |
| Vasopressor medium | 0.00 |  |  |  |
| Vasopressor high | 11.700 | 0.240 | 669.0 |  |
| GFR_Jelliffe | 1.010 | 0.940 | 1.060 | 0.838 |
| CRRT | 23.30 | 0.470 | 5:353 | 0.119 |
| This retrospective observational cohort study was conducted at Na Homolce Hospital, Prague, Czech Republic (January 2019–December 2022). We included consecutive critically ill ICU patients (N = 377) treated with meropenem, cefepime, or piperacillin–tazobactam for acute infections. Antibiotic plasma trough concentrations (Cmin) were measured after 24 h of full dosing using LC–MS/MS. Antibiotic concentrations were related to breakpoints obtained from the European Committee on Antimicrobial Susceptibility Testing (EUCAST). The MIC values for meropenem, cefepime and piperacillin were set at 8 mg/l, 8 mg/l, and 16 mg/l, respectively. Patients who were receiving an every-4-hour dosing regimen were included in subanalysis. Sample size N = 35. BMI – body mass index, CRRT - continuous renal replacement therapy, GFR – glomerular filtration rate, OR = Odds Ratio  **^1^ Overall model fit: LR χ² (-8) = 9.29, p = 0.319; Nagelkerke R² = 0.388; AUC = 0.822 (95% CI 0.599–1); Hosmer–Lemeshow χ² (8) = 8.73, p = 0.366.** | | | | |
